# Modelling human hard palate shape with Bézier curves

**DOI:** 10.1371/journal.pone.0191557

**Published:** 2018-02-15

**Authors:** Rick Janssen, Scott R. Moisik, Dan Dediu

**Affiliations:** 1 Language and Genetics Department, Max Planck Institute for Psycholinguistics, Nijmegen, The Netherlands; 2 Linguistics and Multilingual Studies, Nanyang Technological University, Singapore, Singapore; 3 Donders Institute for Brain, Cognition and Behaviour, Nijmegen, The Netherlands; University of California Irvine, UNITED STATES

## Abstract

People vary at most levels, from the molecular to the cognitive, and the shape of the hard palate (the bony roof of the mouth) is no exception. The patterns of variation in the hard palate are important for the forensic sciences and (palaeo)anthropology, and might also play a role in speech production, both in pathological cases and normal variation. Here we describe a method based on Bézier curves, whose main aim is to generate possible shapes of the hard palate in humans for use in computer simulations of speech production and language evolution. Moreover, our method can also capture existing patterns of variation using few and easy-to-interpret parameters, and fits actual data obtained from MRI traces very well with as little as two or three free parameters. When compared to the widely-used Principal Component Analysis (PCA), our method fits actual data slightly worse for the same number of degrees of freedom. However, it is much better at generating new shapes without requiring a calibration sample, its parameters have clearer interpretations, and their ranges are grounded in geometrical considerations.

## Introduction

Human individuals vary in almost every respect, ranging from genetic to anatomical and cognitive, mostly in quantitative terms (see, for example, [1–4]). Concerning genetics, more than 80% of the variation is found among individuals within a population, about 10% between populations within a large geographical region, and just approximately 10% between continents; even so, this variation is continuous, clinal and decreases with increasing distance from Africa, due to a serial founder tracing our recent origins and expansion effect [1, 5, 6]. Such patterns are also visible in the distribution of phenotypic diversity, such as skull measurements [7, 8].

The *vocal tract* (VT) consists grossly of the larynx, the pharynx, the oral and nasal cavities, and is essential for the production of speech [9]. There are few systematic investigations of the patterns of inter-individual and inter-population variation (especially normal variation) in the structure of the various components of the vocal tract (e.g., [10–19]), but the available information suggests that this is probably under-appreciated. Moreover, there are indications that some of this variation has a genetic component [20–23], but that environmental factors, such as the mechanical properties of food, also play a role [24, 25]. Recent investigations have also indicated that anatomical variation in the VT might result in *articulatory accommodation* with or without audible effects on speech production [26–34].

The *hard palate* (HP; the bony roof of the mouth) is a very important component of the VT and it plays a crucial role in the production of many speech sounds. The HP is a horizontal bony structure that separates the oral and nasal cavities, and is formed by the *palatine process of the maxilla* (approximately the anterior three-quarters) and the *horizontal part of the palatine bone*. The ontogeny of the HP is a complex process integrated in the wider oro-facial development, under the control of complex gene networks and environmental factors. This is shown by evo-devo studies and pathologies with a genetic component, one of the most relevant being *cleft palate* (with or without lip clefting) occurring in isolation or part of various syndromes (see, for example, [21, 35–38] and the OMIM [39] entries for “cleft palate” and “palate shape”).

There are indications that various parameters describing HP shape (such as height, width and arch length) vary between individuals [16, 40, 41] and possibly also between populations [42–46]. Moreover, variation between individuals in the shape of the HP might affect articulatory variability [27, 28], the articulation of /∫/ in German [47] of various consonants in Japanese [29], and of the “bunched” versus “retroflex” North American English /r/ [48], among others (see [16] for more examples). Therefore, understanding the patterns of variation of the HP in humans is important for forensics, physical anthropology, speech pathology, but might be relevant for “normal” phonetics and phonology as well.

There are several methods that can be used to fit and summarize the shape of the HP, including *Principal Component Analysis* (PCA; [16]), *classical morphometrics* (CM; e.g., [42, 45]) and, more recently, *geometric morphometrics* (GM; e.g., [49, 50]). PCA and CM are widely used, and vast amounts of data have been collected and described using CM. GM is arguably the only method that truly separates shape and size [51], and is becoming very popular for describing and analyzing biological shape variation.

We describe here a method that models the 2D midsagittal profile of the human HP using *Bézier curves*, designed with the following ordered goals in mind:

1. first, we wanted a parsimonious model of the human HP midsagittal shape with as *few* parameters as possible,
2. second, these parameters should be *meaningful*, in the sense that they should have intuitive interpretations and their ranges should be motivated;
3. third, the method must be able to *generate* curves that, for all (or the vast majority) of the legal parameter values, could be plausible human HP midsagittal shapes;
4. last, it should also be able to *fit and summarize* real HP midsagittal shapes, allowing statistical analyses of the existing inter-individual variation.

Goals (1) and (4) are shared with PCA and GM, goals (2) and (4) with CM, but goal (3) is specific to our method and cannot be fulfilled by PCA, CM or GM without a “calibration” sample and a set of non-obvious constraints and dependencies between the free parameters. Bézier curves are widely used for computer graphics, animations, user interfaces, and even to describe fonts, as they achieve high flexibility with a small number of degrees of freedom. Our choice was based on the four goals enumerated above and on our previous experience with Bézier curves in a computer science context.

While our method captures variation in the midsagittal profile of the human HP, a complete model must also include variation in the coronal shape. In our application of the Bézier model described here to the biomechanical modeling system ArtiSynth [52] (www.artisynth.org) we do model coronal shape using parabolas, but other approaches are also possible.

## Materials and methods

Author contributions: the manual tracing was done using a custom MATLAB [53] script by SRM; the Bézier curve model was designed and implemented in Python 2 [54] by RJ; the statistical analyses and plots reported here were conducted in R [55] by DD.

The Python 2 [54] source code for fitting a Bézier curve to existing data and for generating Bézier curves for given parameter values, and the R [55] code used for the analyses presented here, as well as the anonymized hard palate traces, are freely available as Supplementary Information and on our GitHub repository (https://github.com/ddediu/bezier-hard-palate) under a GPL v2 license (https://www.gnu.org/licenses/old-licenses/gpl-2.0.en.html).

### Bézier curve parametrization

A general Bézier curve of degree *n*, *C*_*n*_, is a parametric curve defined by *n* + 1 control points *β*_0_, *β*_1_, … *β*_*n*_ (in the 2D case each such point has two coordinates, for example, *β*_0_ = (*x*_0_, *y*_0_)) such that the curve always passes through the first (*β*_0_) and the last (*β*_*n*_) points and is tangent there to the *β*_0_ *β*_1_ and *β*_*n* − 1_ *β*_*n*_ lines:

$$\begin{array}{c} C_{n}\left(t\right)=\sum_{i=0}^{n}\beta_{i}B_{i,n}\left(t\right) \end{array}$$ (1)

where *C*_*n*_ is the Bézier curve parametrized by *t* ∈ [0, 1] which varies along the curve, and *B*_*i*,*n*_ are the so-called Bernstein polynomials:

$$\begin{array}{c} B_{i,n}\left(t\right)=\left(\begin{array}{c} n\\ i \end{array}\right)t^{i}{\left(1-t\right)}^{n-i} \end{array}$$ (2)

(see [56, 57] for details).

For any given number (denoted *τ*) of points described by the parameter *t* between 0 and 1 as above (i.e, 0 ≤ *t* ≤ 1 and $t\propto \frac{1}{\tau }$)), we can recursively evaluate the curve in *n* steps, following De Casteljau’s algorithm [58]. If we denote the curve’s *i*^th^ top-level control point as $\beta_{i}^{\left(0\right)}$, a first-level recursion on that point as $\beta_{i}^{\left(1\right)}$, second level recursion as $\beta_{i}^{\left(2\right)}$ etc., we evaluate the curve as in Eq 3 (where 0 ≤ *s* ≤ *n* and 0 ≤ *i* ≤ *n* − *s*).

$$\begin{array}{c} \beta_{i}^{\left(s\right)}=\left(1-t\right)\beta_{i}^{\left(s-1\right)}+t\beta_{\left(i+1\right)}^{\left(s-1\right)} \end{array}$$ (3)

**Fig 1:**
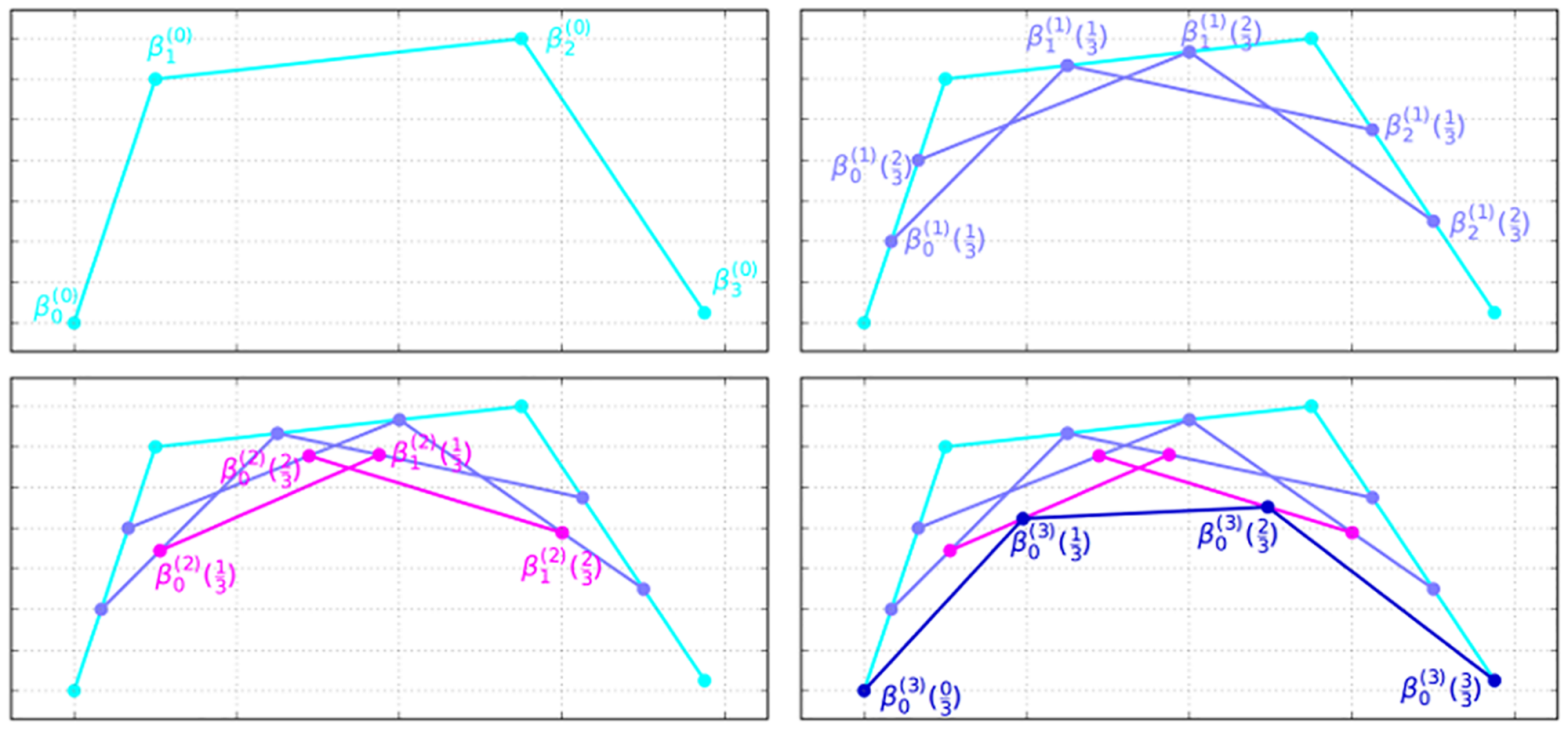
Bézier curve formation following de Casteljau’s algorithm. Shown are the (top-level) control points $\beta_{i}^{\left(0\right)}$ and the recursive control points $\beta_{i}^{\left(1\right)}$, $\beta_{i}^{\left(2\right)}$, and $\beta_{i}^{\left(3\right)}$. Number of sampling points is set at *τ* = 3. For clarity, intervals $t=\frac{0}{3}$ and $t=\frac{3}{3}$ are not shown, except for *β*^(3)^ in panel **d**. The four figure panels show the sequential application of the algorithm: top-left **(a)**: $\beta^{\left(0\right)}=\langle \beta_{0}^{\left(0\right)},\beta_{1}^{\left(0\right)},\beta_{2}^{\left(0\right)},\beta_{3}^{\left(0\right)}\rangle$; top-right **(b)**: $\beta_{n}^{\left(1\right)}=\left(1-t\right)\beta_{n}^{\left(0\right)}+t\beta_{n+1}^{\left(0\right)}$; bottom-left **(c)**: $\beta_{n}^{\left(2\right)}=\left(1-t\right)\beta_{n}^{\left(1\right)}+t\beta_{n+1}^{\left(1\right)}={\left(1-t\right)}^{2}\beta_{n}^{\left(0\right)}+2\left(1-t\right)t\beta_{n+1}^{\left(0\right)}+t^{2}\beta_{n+2}^{\left(0\right)}$; and bottom-right **(d)**: $\beta_{n}^{\left(3\right)}=\left(1-t\right)\beta_{n}^{\left(2\right)}+t\beta_{n+1}^{\left(2\right)}={\left(1-t\right)}^{3}\beta_{n}^{\left(0\right)}+3{\left(1-t\right)}^{2}t\beta_{n+1}^{\left(0\right)}+3\left(1-t\right)t^{2}\beta_{n+2}^{\left(0\right)}+t^{3}\beta_{n+3}^{\left(0\right)}$.

For example, if we want to calculate a quadratic Bézier curve, we might recursively derive it as in Eq 4 (Fig 1).

$$\begin{array}{c} \begin{array}{cc} \beta^{\left(0\right)} & =\langle \beta_{0}^{\left(0\right)},\beta_{1}^{\left(0\right)},\beta_{2}^{\left(0\right)},\beta_{3}^{\left(0\right)}\rangle \\ \beta_{i}^{\left(1\right)} & =\left(1-t\right)\beta_{i}^{\left(0\right)}+t\beta_{i+1}^{\left(0\right)}\\ \beta_{i}^{\left(2\right)} & =\left(1-t\right)\beta_{i}^{\left(1\right)}+t\beta_{i+1}^{\left(1\right)}\\ & ={\left(1-t\right)}^{2}\beta_{i}^{\left(0\right)}+2\left(1-t\right)t\beta_{i+1}^{\left(0\right)}+t^{2}\beta_{i+2}^{\left(0\right)}\\ \beta_{i}^{\left(3\right)} & =\left(1-t\right)\beta_{i}^{\left(2\right)}+t\beta_{i+1}^{\left(2\right)}\\ & ={\left(1-t\right)}^{2}\beta_{i}^{\left(1\right)}+2\left(1-t\right)t\beta_{i+1}^{\left(1\right)}+t^{2}\beta_{i+2}^{\left(1\right)}\\ & ={\left(1-t\right)}^{3}\beta_{i}^{\left(0\right)}+3{\left(1-t\right)}^{2}t\beta_{i+1}^{\left(0\right)}+3\left(1-t\right)t^{2}\beta_{i+2}^{\left(0\right)}+t^{3}\beta_{i+3}^{\left(0\right)} \end{array} \end{array}$$ (4)

**Fig 2:**
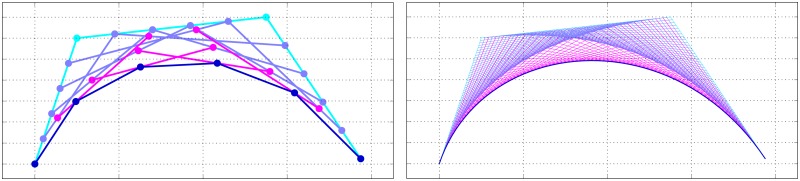
Cubic Bezier curves with lower and higher spatial sampling intervals. The left panel **(a)** shows $\tau =\frac{1}{5}$, while the right panel **(b)** shows $\tau =\frac{1}{50}$ (control points are not shown).

**Fig 3:**
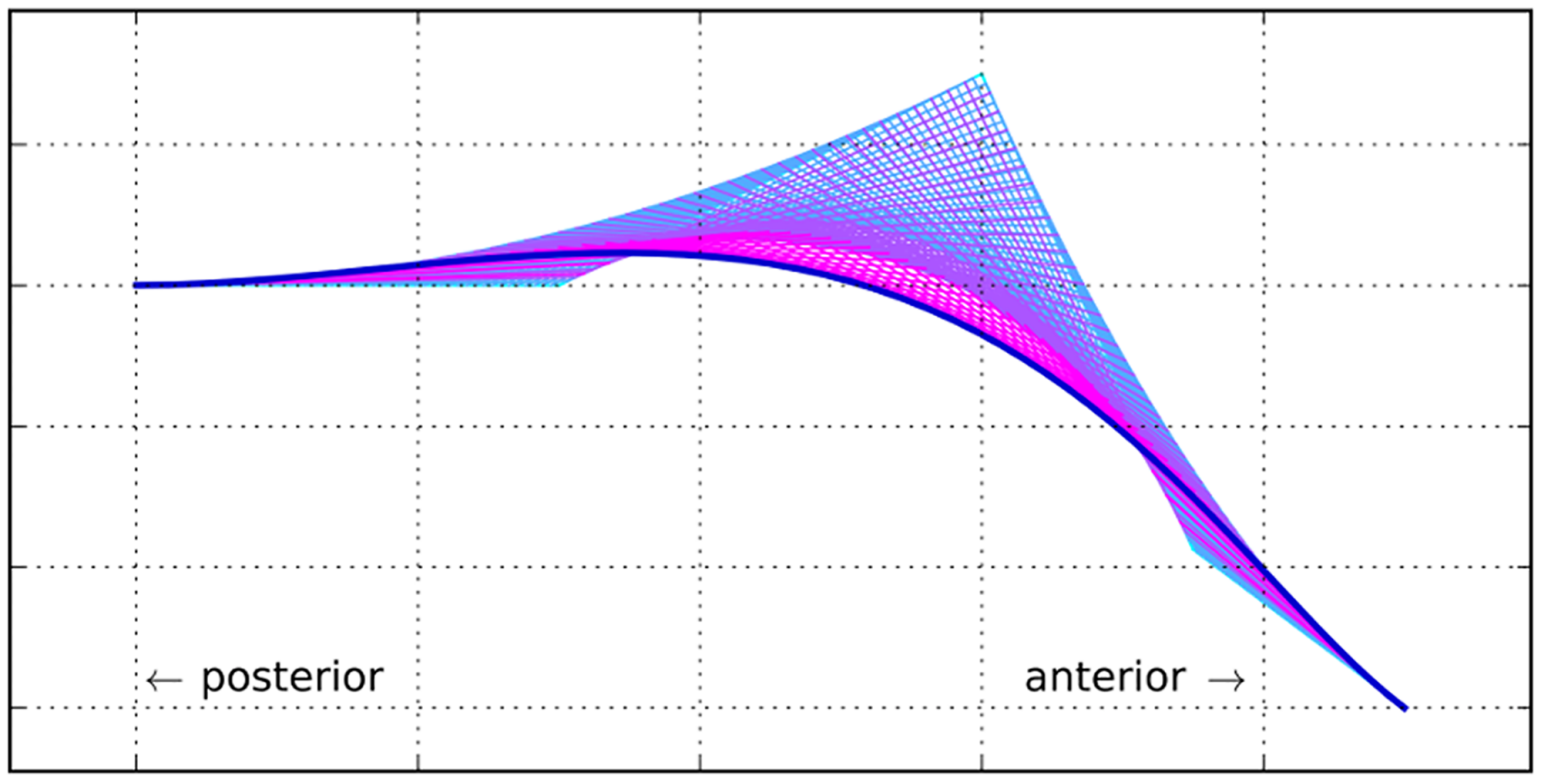
The hard palate Bezier curve is a 4^th^-order curve (shown with *τ* = 50).

By sampling *t* at a higher interval (Fig 2) we can increase spatial resolution. By increasing the number of control points, we can create higher-order curves. The curve we use to model the hard palate is a 4^th^-order curve (Fig 3), defined by the control points shown in Eq 5, where *fixed* control points are denoted as 〈*x*, *y*〉 and *variable* control points as $\beta_{i}^{\left(0\right)}$.

$$\begin{array}{c} \beta^{\left(0\right)}=\left\langle \left\langle -0.2,0.6\right\rangle ,\left\langle 0.1,0.6\right\rangle ,\beta_{2}^{\left(0\right)},\beta_{3}^{\left(0\right)},\left\langle 0.7,0.3\right\rangle \right\rangle \end{array}$$ (5)

**Fig 4:**
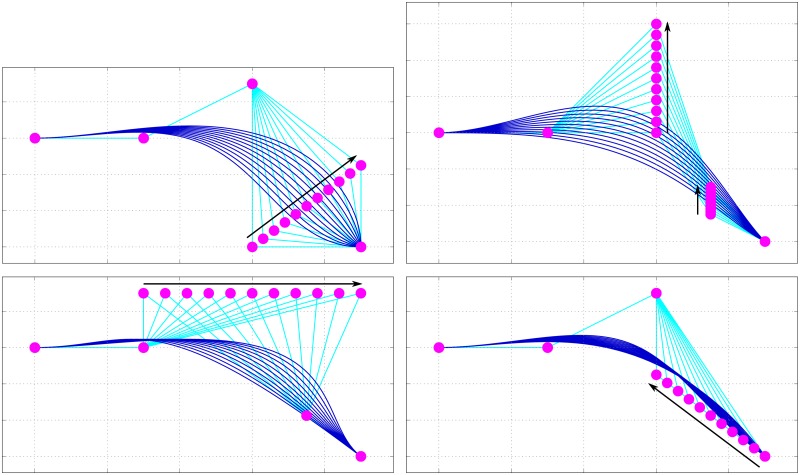
The effect of changing a *single* parameter on the Bézier hard palate model. Each panel shows the change in the curve resulting from incrementing a single parameter in the range 0.0, 0.1, …, 1.0 (in the direction of the arrow). In each case, the three parameters not subject to adjustment were all set to 0.5. The panels are: top-left **(a)**: Incrementing *Alveolar angle*; top-right **(b)**: Incrementing *Palatal concavity*; bottom-left **(c)**: Incrementing *Palatal fronting*; bottom-right **(d)**: Incrementing *Alveolar weight*.

**Fig 5:**
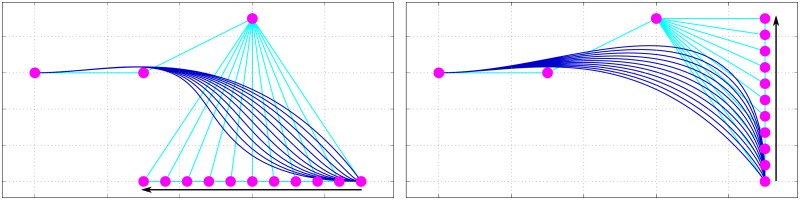
The effect on the curve when changing *Alveolar weight*, with *Alveolar angle* set to its extremes (compare to Fig 4 with *Alveolar angle* set to 0.5, and with *Alveolar weight* fixed to 0.5, respectively, while changing *Alveolar angle*). *Palatal concavity* and *Palatal fronting* are set to 0.5. The panels are: left **(a)**: Palatalangle=0, and right **(b)**: Palatalangle=1.

Using four parameters *Palatal fronting*, *Palatal concavity*, *Alveolar angle* and *Alveolar weight*, each with a corresponding value 0 ≤ *p* ≤ 1, we change the position of the two variable control points $\beta_{2}^{\left(0\right)}$ and $\beta_{3}^{\left(0\right)}$ (Eq 5), thereby changing the appearance of the curve in a continuous manner, and with various interactions (Figs 4 and 5). It is important to note that the variable control points are completely defined in the context of the fixed control points and the four parameters we just introduced.

**Alveolar angle** (*angle*, *“a”*) controls the angle of inclination of the alveolar ridge (shelf-like prominence of the alveolar margin) from 180° (approximating a sigmoidal profile) to 90° (approximating a parabolic profile) by adjusting the horizontal as well as vertical positions of $\beta_{3}^{\left(0\right)}\left(x\right)$, following the parameter value *p*_a_ (Eqs 6, 7 and Fig 4 panel **a**). The effects of *angle* have to be considered in conjunction with that of *weight*.
  $$\begin{array}{c} \beta_{3}^{\left(0\right)}\left(x\right)=\left(1-p_{\text{a}}\right)(\beta_{4}^{\left(0\right)}\left(x\right)-\beta_{1}^{\left(0\right)}\left(x\right)) \end{array}$$ (6)
  $$\begin{array}{c} \beta_{3}^{\left(0\right)}\left(y\right)=p_{\text{a}}(\beta_{4}^{\left(0\right)}\left(x\right)-\beta_{2}^{\left(0\right)}\left(x\right)) \end{array}$$ (7)
**Palatal concavity** (*concavity*, *“c”*) increases the vertical displacement between the junction of the hard and soft palates and palatal roof, by adjusting the vertical positions of $\beta_{2}^{\left(0\right)}$ and (implicitly, through *angle* and *weight*) $\beta_{3}^{\left(0\right)}$, following the parameter value *p*_c_ (Eq 8), where effectively $\beta_{1}^{\left(0\right)}\left(y\right)\leq \beta_{2}^{\left(0\right)}\left(y\right)\leq (\beta_{1}^{\left(0\right)}\left(y\right)-\beta_{4}^{\left(0\right)}\left(y\right))+\beta_{1}^{\left(0\right)}\left(y\right)$ and (in conjunction with *angle* and *weight*) $\beta_{4}^{\left(0\right)}\left(y\right)\leq \beta_{3}^{\left(0\right)}\left(y\right)\leq \beta_{2}^{\left(0\right)}\left(y\right)$. In more concrete terms, larger values of *p*_c_ increase the doming of the hard palate. A value of *p*_c_ = 0 means the palate can only monotonically decline moving from the velum towards the incisors (Fig 4 panel **b**).
  $$\begin{array}{c} \beta_{2}^{\left(0\right)}\left(y\right)=p_{\text{c}}(\beta_{1}^{\left(0\right)}\left(y\right)-\beta_{4}^{\left(0\right)}\left(y\right))+\beta_{1}^{\left(0\right)}\left(y\right) \end{array}$$ (8)
**Palatal fronting** (briefly *fronting*, and denoted as *“f”*) shifts the palatal roof more anteriorly for higher values, by adjusting the horizontal position of $\beta_{2}^{\left(0\right)}$, following the parameter value *p*_f_ (Eq 9), where effectively $\beta_{1}^{\left(0\right)}\left(x\right)\leq \beta_{2}^{\left(0\right)}\left(x\right)\leq \beta_{4}^{\left(0\right)}\left(x\right)$. Depending on the other parameter values, this generally results in steeper inflections of the palate for higher values of *p*_f_ (Fig 4 panel **c**).
  $$\begin{array}{c} \beta_{2}^{\left(0\right)}\left(x\right)=p_{\text{f}}(\beta_{4}^{\left(0\right)}\left(x\right)-\beta_{1}^{\left(0\right)}\left(x\right))+\beta_{1}^{\left(0\right)}\left(x\right) \end{array}$$ (9)
**Alveolar weight** (*weight*, *“w”*) modifies the ‘magnitude’ of *angle*, following the parameter value *p*_w_ (Eqs 10, 11 and Fig 4 panel **a**). Together with *angle*, it effectively holds that $\beta_{1}^{\left(0\right)}\left(x\right)\leq \beta_{3}^{\left(0\right)}\left(x\right)\leq \beta_{4}^{\left(0\right)}\left(x\right)$ and $\beta_{4}^{\left(0\right)}\left(y\right)\leq \beta_{3}^{\left(0\right)}\left(y\right)\leq \beta_{2}^{\left(0\right)}\left(y\right)$. For example, with a sigmoidal profile (*p*_a_ < 0.5) the onset of the upward inflection coming from the incisors gets shifted more posteriorly for higher values of *p*_w_ (Fig 5 panel **a**). With a parabolic profile (*p*_a_ > 0.5) the vertical onset (coming from the incisors) gets amplified and in effect becomes steeper (Fig 5 panel **b**). If *p*_w_ = 0, *angle* is neutralized.
  $$\begin{array}{c} \beta_{3}^{\left(0\right)}\left(x\right)=\beta_{4}^{\left(0\right)}\left(x\right)-p_{\text{w}}\beta_{3}^{\left(0\right)}\left(x\right) \end{array}$$ (10)
  $$\begin{array}{c} \beta_{3}^{\left(0\right)}\left(y\right)=\beta_{4}^{\left(0\right)}\left(y\right)-p_{\text{w}}\beta_{3}^{\left(0\right)}\left(y\right) \end{array}$$ (11)

Since the position the variable control points may depend on multiple parameters, the order in which the effects of these parameters is computed is important. More specifically, it holds that {*p*_f_, *p*_c_}≺*p*_a_ ≺ *p*_w_.

The actual interactive Python script is given in S2 Script.

### Systematically generating Bézier curves

In order to explore the variety of curves generated by our approach, we have systematically produced all the Bézier curves corresponding to a fine discretization of the parameter space.

For each of the four parameters, “angle”, “concavity”, “fronting” and “weight”, we considered 51 equally spaced values between 0.0 and 1.0 (0.00, 0.02, 0.04, 0.06, … 0.96, 0.98, 1.00), resulting in 51^4^ = 6,765,201 unique combinations of parameter values. For each combination of parameter values, we generated the corresponding Bézier curve passing through the fixed leftmost and rightmost points (0, 0) and (1, *h* = 0.311 = arctan(0.322)), respectively (the 0.322 radians angle is due to the internal representation of the Bézier curves by the algorithm and is arbitrary). We then discretized this curve at 100 equidistant positions on the *x*-axis between 0.0 and 1.0, resulting in 100 points *β*_0_ = (0, 0), *β*_1_ = (*x*_1_, *y*_1_), … *β*_100_ = (1.0, 0.311). The minimum and maximum *y*-coordinates of these points are used to define the $\frac{x}{y}$ ratio *r* = (max_*i* = 1‥100_(*y*_*i*_) − min_*i* = 1‥100_(*y*_*i*_))^−1^ used to rescale the *y*-coordinate values. These 6,765,201 discretized and normalized Bézier curves can be found in Zenodo at https://doi.org/10.5281/zenodo.1154779.

To interactively explore the structure of these systematically generated Bézier curves, we wrote an R [55] script designed for Rstudio [59] using the library manipulate [60], which allows the real-time manipulation of the values of the four parameters and displays the corresponding Bézier curve (for details see S1 Script).

### Participants and hard palate tracing

Our data is composed of two datasets. The first comprises 22 MRI scans reported in [31] from native speakers of American English for which the gender and age are given in the paper, together with sufficiently high resolution midsagittal (and smaller coronal) MRI images acquired during the production of American English /r/. The second contains 85 MRI structural scans from the *ArtiVarK* sample, covered by amendment 45659.091.14 (1 June, 2015) “ArtiVarK: articulatory variation in speech and language” to the ethics approval “Imaging Human Cognition”, Donders Center for Brain, Cognition and Behaviour, Nijmegen, approved by CMO Regio Arnhem-Nijmegen, The Netherlands. The MRI scans were acquired at the Donders Institute for Brain, Cognition and Behaviour, Nijmegen, The Netherlands, using a 1.5T MAGNETOM^®^Avanto Siemens (http://www.healthcare.siemens.com/magnetic-resonance-imaging/0-35-to-1-5t-mri-scanner/magnetom-avanto); these are high-resolution structural T1 scans (T1 MPR NS PH8, TE = 2.98ms, TR = 2250ms, flip angle 15°, slice thickness 1mm, pixel spacing 1mm x 1mm, FOV 256 x 256), but we used here a JPEG image of the midsagittal slice to ensure comparability with the other 22 scans.

**Fig 6:**
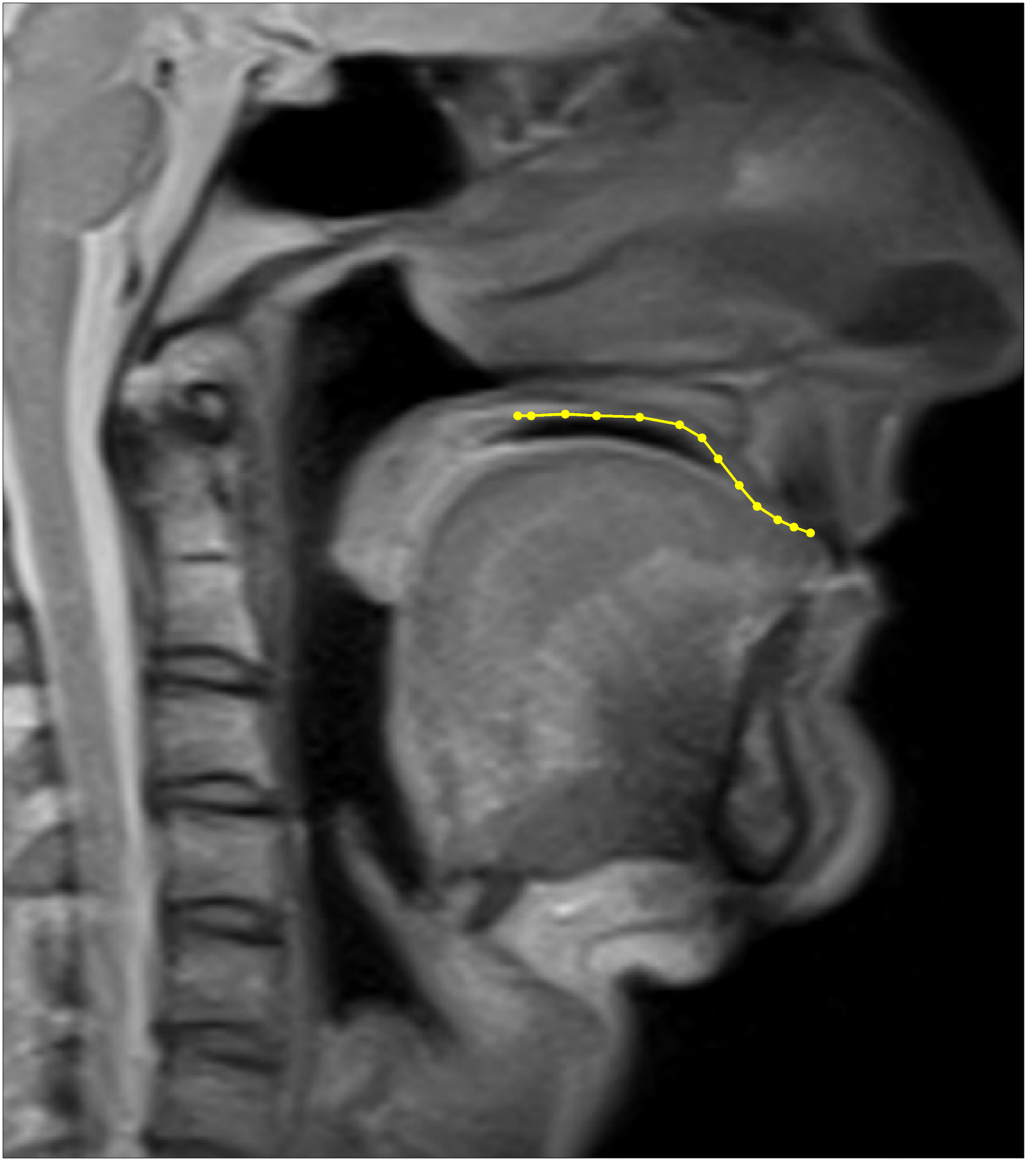
Mid-sagittal MRI scan with tracing. An example of a midsagittal MRI scan (41 years old male) also showing one manual tracing (in yellow; the circles represent the actually sampled points and the lines connect consecutive points).

For each of the 107 MRI scans we worked with one midsagittal slice image that captured the full hard palate (an example is given in Fig 6). Slices were oriented so that the teeth appear on the right of the image while the pharynx/posterior portion of the hard palate appears on the left. In each such image, the hard palate was then manually traced by SRM using a custom-written MATLAB^®^ [53] script, resulting in a sequence of 2D points (ranging between 8 and 25 across tracings) such that the contour connecting them best approximates (as judged visually) the shape of the hard palate shown in the slice. Given the mixed nature of the data (with variable structural visibility), a strictly consistent definition of the hard palate was not possible. We defined the midsagittal contour of the hard palate as beginning posteriorily underneath the posterior nasal spine and/or junction point of the posterior border of the vomer bone with the palatine bones (depending on visibility). The anterior point of the hard palate was defined as the gingival margin of the central maxillary incisors. A source of potential difficulties (also visibile in Fig 6) is that sometimes the tongue was in contact with the palate, making it difficult to unambiguously identify the palate contour. To control for any possible error arising from tracing inconsistency because of the mixed data, SRM performed three repetitions of the tracing process. This resulted in a total of 107 × 3 = 321 tracings.

Each tracing is uniquely denoted using a “T” for the data from Tiede and colleagues [31] and an “A” for the *ArtiVarK* participants, followed by the numeric participant id, and, if needed, the tracing (1 to 3) preceded by a dot “.”; for example “A01.1” represents the first tracing for participant ID 01 from the *ArtiVarK* dataset.

### Normalizing and resampling the tracings

The tracings are in the slice image’s own coordinate system and, in order to ensure comparability, we normalized them as follows: (i) we first rotated around the tracing’s midpoint such that the right-most point (i.e., the beginning of the alveolar ridge) has the same height as the left-most point, followed by (ii) a translation so that the left-most point has an *x*-coordinate of 0 and the lowest point of the tracing a *y*-coordinate of 0, ending with (iii) the independent scaling on the two axes such that the horizontal and vertical lengths of the tracing are 1.0 (i.e., the *x*-coordinate of the right-most point is 1 and the *y*-coordinates of the lowest and highest points and 0.0 and 1.0 respectively). These tracings can be found in Zenodo at https://doi.org/10.5281/zenodo.1154779, where we give the coordinates of the leftmost and rightmost points of the tracing (in the original image coordinates in pixels), the rotation (in radians), and the normalized *x* and *y* coordinates of their points.

However, for Bézier curve generation and some of the statistical analyses, we needed to make sure that all normalized tracings have the same number of sample points at the same x-coordinates by resampling at *m* (in most such cases *m* = 100) equidistant positions on the x-axis between 0.0 and 1.0. More precisely, given a tracing described by the 0 < *n* + 1 ≤ *m* 2D points *β*_0_ = (0, 0), *β*_1_ = (*x*_1_, *y*_1_), … *β*_*n* − 1_ = (*x*_*n* − 1_, *y*_*n* − 1_), *β*_*n*_ = (1, 0), we computed the intersection between the verticals at each of the *m* equidistant positions on the *x*-axis $x_{i}^{\prime }=\frac{i}{n-1}$, *i* ∈ {0, 1, …*n* − 1} with the corresponding segment of the tracing *β*_*j*_ *β*_*j*+1_ such that $x_{j}\leq x_{i}^{\prime }\leq x_{j+1}$ (the general procedure is that if there is more than one such segment, we would pick the one with the smallest *j*, but this does not occur in our samples), resulting in a set of y coordinates $y_{i}^{\prime }=y_{j}+\left(y_{j+1}-y_{j}\right)\frac{x_{i}^{\prime }-x_{j}}{x_{j+1}-x_{j}}$. This process ensures that all the tracings are sampled at the same *m* equidistant *x*-coordinates always starting and ending at *y*-coordinate 0, making them easy to align and compare.

### Fitting a Bézier curve to a tracing

Given a normalized tracing defined by *n* + 1 2D points *β*_0_ = (0, 0), *β*_1_ = (*x*_1_, *y*_1_), … *β*_*n* − 1_ = (*x*_*n* − 1_, *y*_*n* − 1_), *β*_*n*_ = (1, 0), we fit a four-parameter Bézier curve using a Genetic Algorithm [61] as follows. The “genome” has four real-number “genes” (taking values between 0.0 and 1.0) representing the four parameters of the Bézier curve “angle”, “concavity”, “fronting” and “weight”, and the “fitness function” is computed by first generating the Bézier curve defined by the current values of the four parameters, followed by discretization into *n* + 1 $y_{i}^{b}$ *y*-coordinates on the Bézier curve corresponding to the *n* + 1 *x*_*i*_ *x*-coordinates, and the computation of the mean squared error (*MSE*) between the discretized Bézier curve and the tracing

$$\begin{array}{c} MSE=\frac{1}{n}\sum_{i=0}^{n}{\left(y_{i}-y_{i}^{b}\right)}^{2} \end{array}$$ (12)

which represents the genome’s fitness value. In our runs we used a population size of 100 genomes for 1000 generations (or less), and for each tracing we performed 100 independent replications of the algorithm in order to explore the fitness landscape and prevent being captured by local optima. For each replication we used only the best fitting genome (i.e., the parameter values that minimized the *MSE* between the corresponding Bézier curve and the actual tracing) for analysis; these data are available in the S2 Dataset. The actual parameters used are given in Table 1.

**Table 1:** The parameters for the Genetic Algorithm.

Parameter	Value or representation
Population size	100 agents, initialized with 1000 agents
Genome	Floating-point valued vector *V* = (*a*, *c*, *f*, *w*) ∈ [0, 1]^4^
Mutation	Gaussian (*μ* = 0, *σ* = 0.01)
Recombination	None
Parent selection	Stochastic universal sampling (*s* = 1.25)
Survivor selection	*μ* + *λ* (with elitism)
Termination	generations *g* > 1000, or *g* > 50 & no elite improvement for $\frac{1}{4}g$
N° replications	100

The parameters used by the Genetic Algorithm that fits a Bézier curve to a given tracing, as defined in [62].

### Fixed and free Bézier curve parameters

To investigate the influence of fixing parameters of the Bézier curve on its goodness of fit, we investigated the 16 *conditions* resulting from specifying which parameters are free and which are fixed. In this context, “fixing” a parameter means that it could only have a single given value, while a “free” parameter’s value could be freely adjusted (between 0.0 and 1.0) by the fitting algorithm. In the first pass, we allowed all parameters to be free in order to obtain the globally best-fitting parameter values *a*_*fix*_ = 0.1149, *c*_*fix*_ = 0.5878, *f*_*fix*_ = 0.382, *w*_*fix*_ = 0.7204; these values were then used, in the second pass, for the corresponding fixed conditions.

The 16 conditions are denoted here using the first letter of the fixed parameters, if any; therefore the fully-free condition, “”, means that all parameters are free, condition “a” means that parameter “angle” is fixed, condition “acf” means that the parameters “angle”, “concavity” and “fronting” are fixed (leaving thus only the “weight” parameter free), and the full condition, “acfw”, means that all parameters are fixed. The full list of conditions (the powerset of the four parameters, 2^{“a”, “c”, “f”, “w”}^) is: “”, “a”, “c”, “f”, “w”, “ac”, “af”, “aw”, “cf”, “cw”, “fw”, “acf”, “acw”, “afw”, “cfw”, and “acfw”.

### Evaluating the goodness of fit

Thus, for each tracing in each of the 16 conditions we have 100 independent sets of 4 Bézier curve parameter values (denoted in the following for brevity as *a*, *c*, *f* and *w* for “angle”, “concavity”, “fronting” and “weight”, respectively) that best fit the tracing (i.e., minimize the mean squared error, *MSE*). Because of the normalization, the *MSE* can take values between 0 and $\sqrt{n+1}$, where *n* + 1 is the number of landmarked points in the tracing, and given that our tracings have between 8 and 25 points (mean 21.2), the *MSE* ranges between a minimum of 0.0 and a maximum of 2.83 or 5.00 with a mean of 4.61 (depending on the actual number of points in the tracing); therefore we can consider that 0.0 ≤ *MSE* ≤ 5.0. To obtain a better representation of the expected distribution of the *MSE*, we randomly generated, for each tracing, 10,000 “curves” with the same number of points (i.e., these have random *y*-values for each of the tracing’s points) and computed the *MSE* between the tracing and these “curves”. Across all hard palate profiles (henceforth denoted as “HPP”), tracings and replications, these random *MSE*s vary between 0.0015 and 0.71, with an average of 0.21 and standard deviation of 0.051. Additionally, for each such case we computed the percent of the random *MSE*s smaller than the actual *MSE* between the tracing and the best-fitting Bézier curve, as well as a one-sample *t*-test between this distribution of random *MSE* and the actual *MSE*.

### Relationship between fitted parameters and original tracings

As detailed above, for each condition *C* ∈ { “”, “a”, “c”, “f”, “w”, “ac”, “af”, “aw”, “cf”, “cw”, “fw”, “acf”, “acw”, “afw”, “cfw”, “acfw”} and tracing, there are 100 replications of the fitting process, resulting in 100 sets of bets fitting parameter values. The relationship between these sets of best fitting parameter values might reveal the structure of the parameter space, and we analyzed, on the one hand, the relationship between sets belonging to the same tracing, and, on the other, the relationship between HPPs and their best fitting parameter values.

Therefore, we computed Mantel correlations [63] between, on the one hand, the distances (Euclidean and Procrustes [51]) between the original tracings and, on the other, the Euclidean distance between the parameter values as found by the fitting process (however, given the prohibitive computational costs required for processing all 100 replications for all tracings, we sampled 1,000 random replications, resulting in 1,000 Mantel correlations for the Euclidean distances and 1000 for the Procrustes distances). The Procrustes distances were computed using R’s library shapes [64] and more precisely with the function procOPA which returns the squared root of the ordinary Procrustes sum of squares, and function procGPA which returns the root mean square of the full Procrustes distances to the mean shape. A Mantel correlation of −1.0 indicates a perfect negative correlation between the distances (i.e., when two points are very close together in one space they are very far in the other); 0.0 indicates a complete lack of correlation; 1.0 indicates a perfect correlation between the distances (i.e., when two points are very close together in one space, they are also very close in the other).

A different approach to this question uses concepts from spatial Point Pattern Analysis [65], by testing whether the observed pattern of points (here, sets of best fit parameter estimates) are distributed at random, more clustered, or more dispersed than expected. One approach is to compare the Nearest Neighbor (nn) and mean distances between the actual parameter estimates with the nn and mean distances between 1,000 randomly generated sets of an equal number of parameter estimates. Another is to plot the generalized Ripley’s $\hat{k}$ function (see [66] for details on its generalization to more than two dimensions) showing, at each distance scale (“lag”), whether the data is random, clustered, or dispersed.

### Choosing between conditions

By fixing various combinations of our four free parameters (“a”, “c”, “f”, and “w”), we have 16 possible conditions (“”, “a”, “c”, “f”, “w”, “ac”, “af”, “aw”, “cf”, “cw”, “fw”, “acf”, “acw”, “afw”, “cfw”, and “acfw”) that can be used to fit a set of HPPs. We would like to be able to chose a set of free parameters that is minimal (in line with Occam’s razor and reduced computational costs of the fitting process) but still produces a good fit to the data.

Simply comparing the distribution of *MSE* across conditions is not well-suited given that it is expected that conditions with more free parameters fit the data better. A popular approach is to use methods based on information theory that simultaneously consider the model’s fit to the data and its complexity, the best-known [67] being *Akaike’s Information Criterion* (*AIC*) and the *Bayesian (or Schwarz’s) Information Criterion* (*BIC*).

*AIC* is defined as *AIC* = 2*k* − 2 ln(*L*) and *BIC* is *BIC* = *ln*(*n*) ⋅ *k* − 2 ln(*L*), where *k* is the number of free parameters of the model, *L* is the maximum likelihood of the model for the observed data, and *n* is the number of observations. However, we cannot directly compute the likelihood of our model for the given data, but we can estimate the −2 ln(*L*) term using the squared sum of errors ([68]; see also http://www.r-bloggers.com/genestim-a-simple-genetic-algorithm-for-parameters-estimation/ and http://stats.stackexchange.com/questions/16508/calculating-likelihood-from-rmse), resulting in estimates linearly proportional to 2*k* + *n* ⋅ ln(*MSE*) and ln(*n*) ⋅ *k* + *n* ⋅ ln(*MSE*), respectively. With these, we obtain estimates of the *AIC* and *BIC* for each replicate, and we can compare their distribution for different conditions, choosing the condition that is significantly better, having a lower *AIC* (or *BIC*) estimate at a threshold of 5 points.

### Comparing PCA and Bézier curve fit

In order to compare our method and the classic PCA [69] approach, we fitted both methods to the same 107 MRI midsagittal HP tracings.

For the PCA method, we first aligned and resampled (at *n* equidistant points) all the normalized tracings. It is sometimes suggested that for PCA the number of variables must be relatively low compared to the number of observations (but see, for example, https://www.encorewiki.org/display/~nzhao/The+Minimum+Sample+Size+in+Factor+Analysis for a discussion) and experiments we have conducted varying *n* have suggested that a good accuracy is achieved for *n* = 25. We then conducted PCA with the corresponding *y*-coordinate values at each of the *n* resampled *x*-coordinate locations as the variables, and the tracings as the observations.

Then, for each tracing we reconstructed the *y*-coordinates corresponding to the *n* resampled points when using the first *l* PCs (here, *l* ∈ {1, 2, 3}), *PC*_1_, *PC*_2_, … *PC*_*l*_, and the tracing’s specific loadings on these PCs. We then computed the mean standard error (*MSE*) between the actual *y*-coordinates of the tracing and the *y*-coordinates of the reconstruction.

Finally, we compared these *l*-PC-based *MSE*s with the distribution of *MSE*s obtained by our method in all 16 conditions.

## Results

### Generating possible hard palate shapes

**Fig 7:**
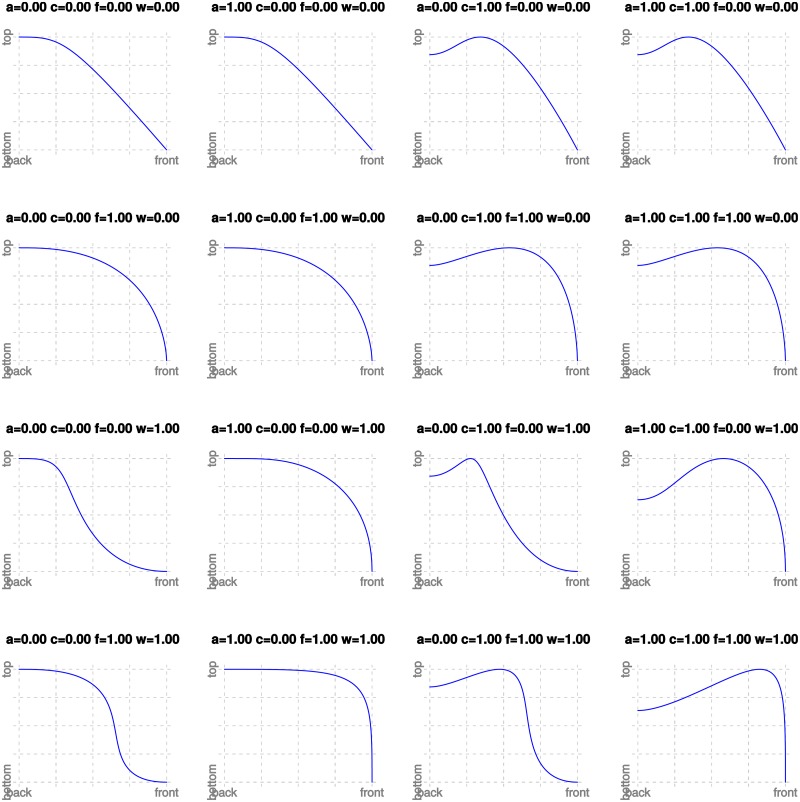
The most extreme Bézier curves. These are the Bézier curves generated by our method for the most extreme possible values of the four parameters (namely 0.0 and 1.0).

Fig 7 shows the Bézier curves generated by the most extreme values of the four parameters (the corners of the 4-dimensional hypercube [0, 1]^4^) and S1 Script contains an interactive R [55] script for the visualization of the whole parameter space. It can be seen that even the extreme cases do not visually seem to be completely impossible shapes of human hard palates, but, as discussed below in more detail, the actual tracings of real human HPPs do not cover the parameter space, suggesting that some regions are more “natural” than others.

**Fig 8:**
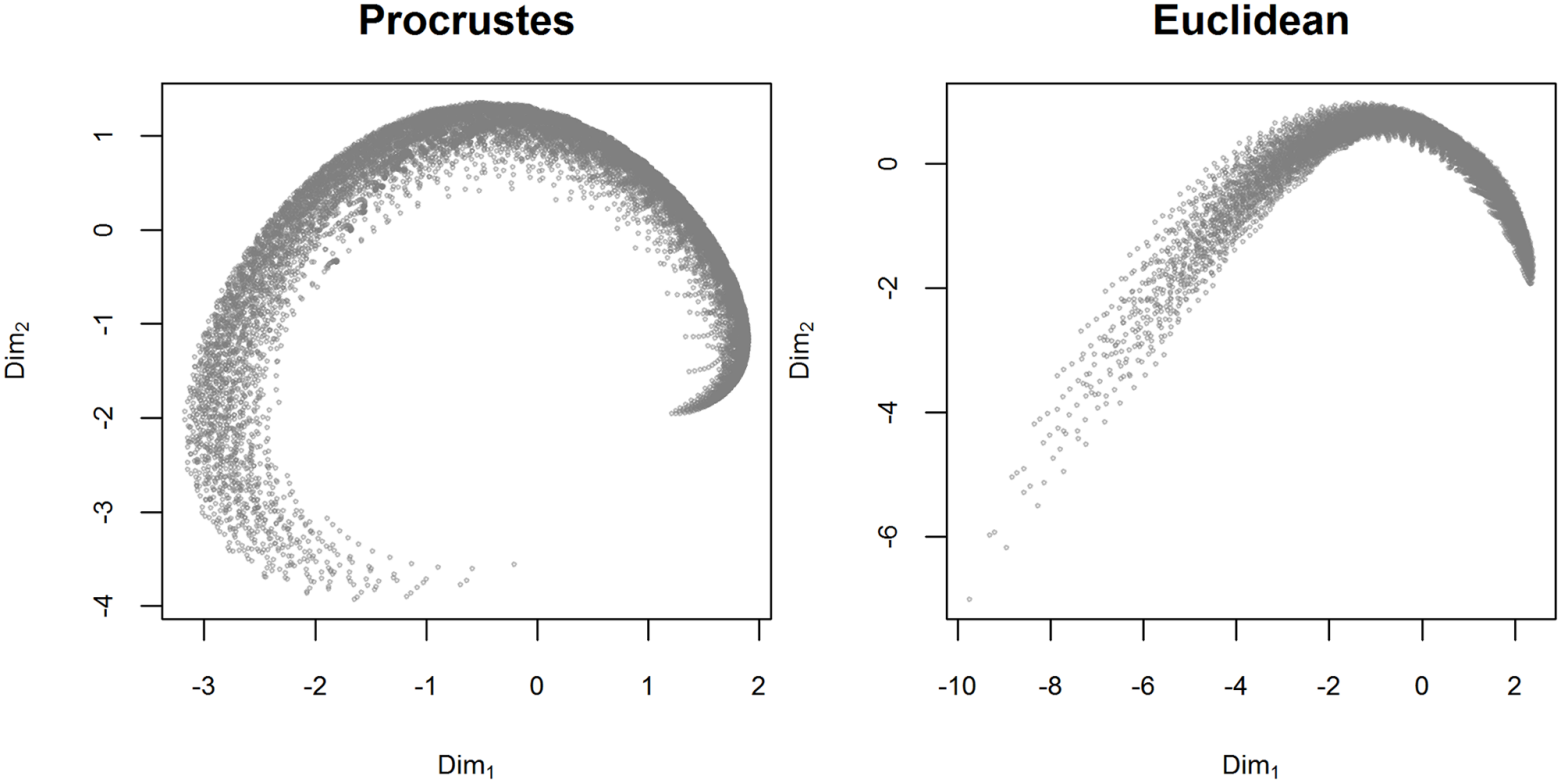
MDS of distances between the generated Bézier curves. The left-hand panel shows the two-dimensional Multidimensional Scaling (MDS) projection of the Procrustes distances between the generated Bézier curves, while the right-hand panel shows the 2D MDS projection of the Euclidean distances between the curves’ parameter values (a 4-dimensional space). Due to computational constraints, we used a reduced set of 11^4^ = 14,641 equally spaced parameter values.

Due to the prohibitive computational costs of processing all 51^4^ = 6,765,201 parameter values, we considered a reduced set of 11 equally spaced points resulting in 11^4^ = 14,641 parameter values. In this reduced set, for all possible pairs of parameter values we computed the Procrustes distance between the generated Bézier curves as well as the Euclidean distance between the corresponding parameter values. There is a very high and significant Mantel correlation (computed with 1,000 permutations) between these two sets of distances (Pearson’s *r* = 0.98 and Spearman’s *ρ* = 0.99, for both *p* < 10^−4^), showing that the difference between the generated Bézier curves corresponds to the difference in the parameter values of the model used to generate them. Fig 8 contains the two-dimensional Multidimensional Scaling (MDS) of the Procrustes distances between the generated Bézier curves (left-hand panel) and of the Euclidean distances between the curves’ parameter values (right-hand panel), showing that they are relatively similar.

**Fig 9:**
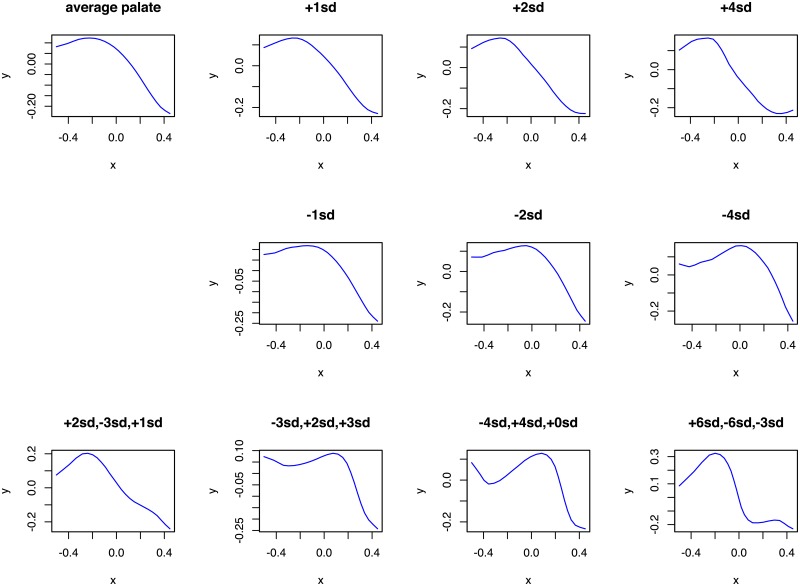
Possible hard palate shapes generated from the first three Principal Components derived from our sample of MRI scans using various weights. The first panel (top left, “average palate”) shows the curve generated using the average loadings across the sample on the first three PCs (${\bar{w}}_{i}$, *i* ∈ {1, 2, 3}), while the following three top and three mid panels show the curves generated going the same number of standard deviations away (positive or negative) from the sample averages (i.e., the panel “+1sd” was constructed with weights ${\bar{w}}_{i}+1.0\mathrm{sd}\left(w_{i}\right)$). The bottom four panels show various combinations (in no particular order) of deviations (in terms of standard deviations) from the sample average. It can be seen that while the curves generated in the neighborhood of the sample average seem plausible human HPPs, the farther away one deviates from this average, the less plausible these shapes become.

PCA can also be used to generate new hard palate shapes: given a sample of HPPs, one extracts the first PCs, PC_*i*_, that explain most of the variance and, using new loadings, $w_{i}\in R$, computes the resulting shape ∑_*i*_ *w*_*i*_PC_*i*_. However, while the shapes generated using loadings *w*_*i*_ in the neighborhood of the actual loadings of the sample HPPs are quite realistic, they become less and less so the more different the *w*_*i*_’s are to the actual loadings (Fig 9). It is unclear what the range of possible loadings *w*_*i*_ is, and the vast majority of shapes generated with this procedure do not seem to represent valid human hard palates. Moreover, in order to use this PCA-based generation procedure, one needs first to extract the PCs and their loadings from a particular sample, making the procedure dependent on this “calibration” sample.

### Tracing midsagittal hard palate profiles

**Fig 10:**
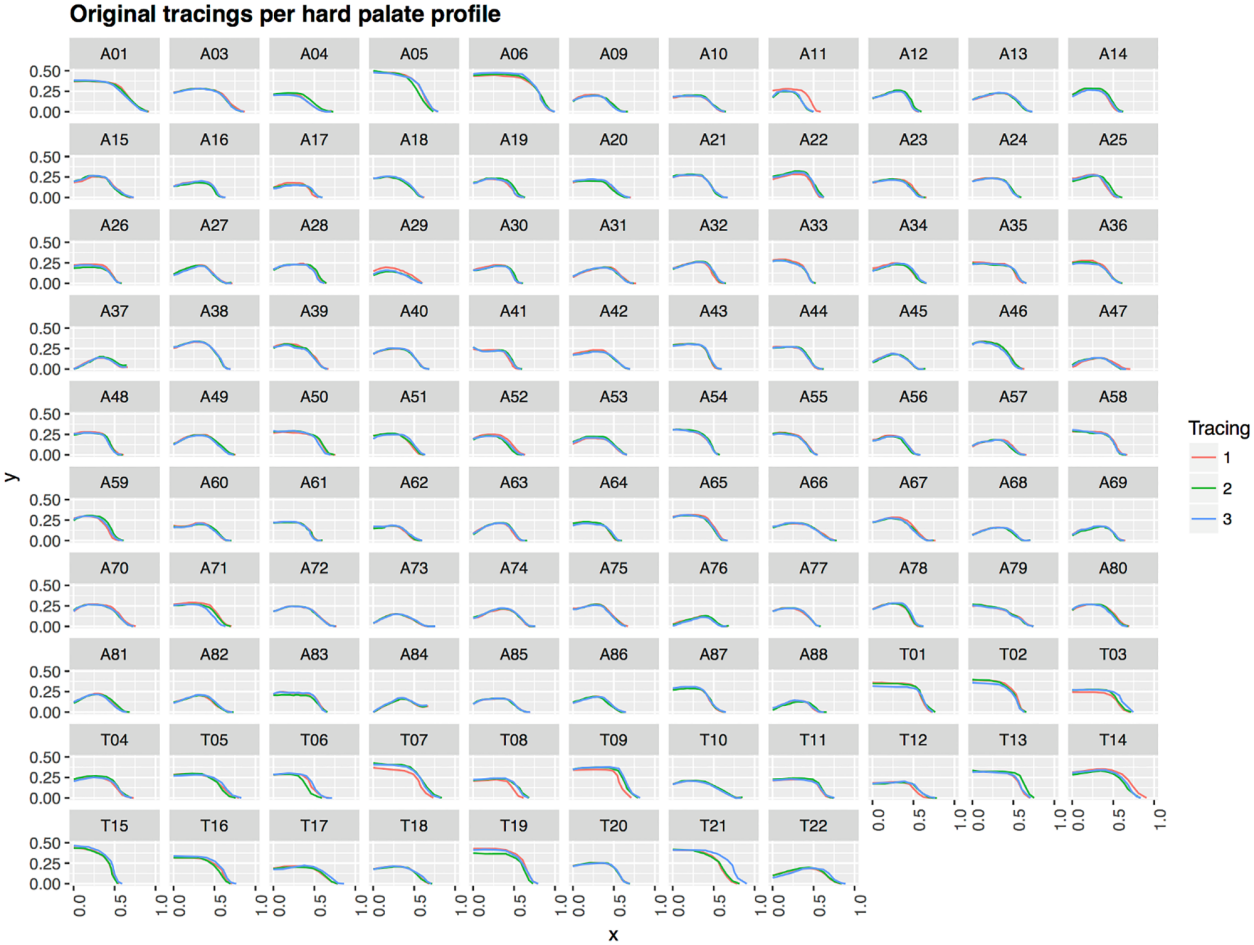
Original tracings per hard palate profile. The three independent original replication tracings per HPP are shown with different colors. The tracings are oriented with the alveolar ridge to the right. The *x* and *y* coordinates have been mirrored to respect the conventions in this paper and are scaled respecting the original x/y scale. Fig 11 shows the normalized tracings allowing a better view of how the shape varies across the traces.

**Fig 11:**
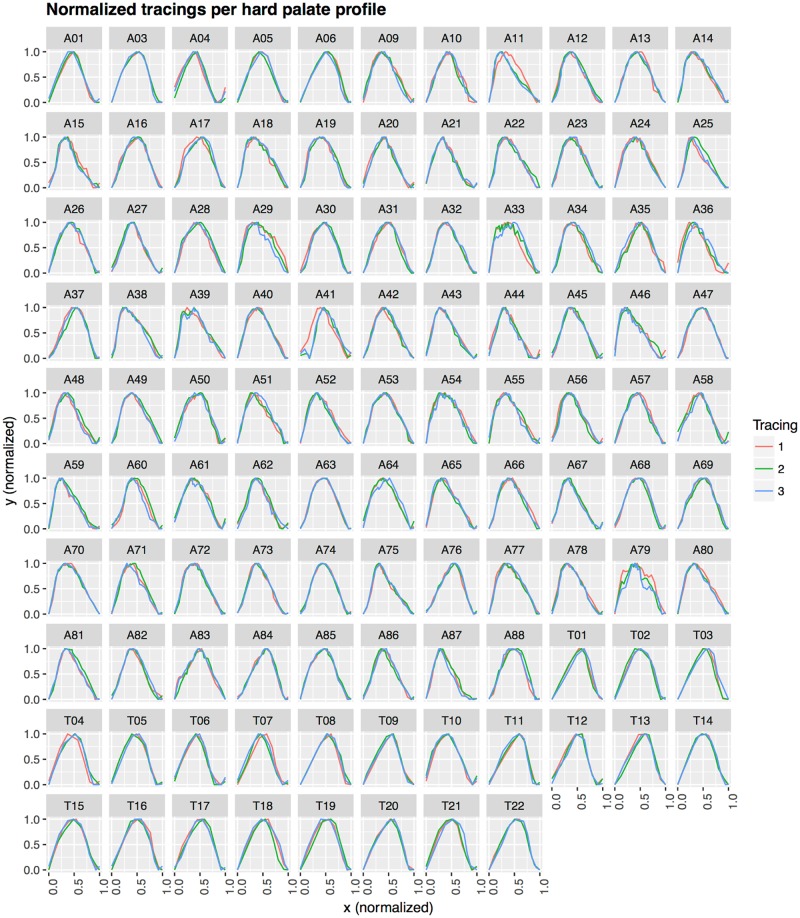
Normalized tracings per hard palate profile. This uses the same conventions as Fig 10 except that now the *x* and *y* coordinates are normalized to the interval [0, 1] (i.e., translated, rated and scaled). Here, it is clearer that the three replication tracings are quite consistent but there are also slight differences between them.

Fig 10 (actual data available in S1 Dataset) shows the three manual tracings of the 107 HP midsagittal profiles, while Fig 11 shows them normalized, facilitating their comparison. Visually, it the three tracings are very similar, but not identical: there are very slight differences between them.

Table 2 shows the Pearson’s correlations, Euclidean distances and Procrustes distances [51] between all pairs of normalized tracings (i.e., tracing 1 versus tracing 2, 1 vs. 3, and 2 vs. 3), as well as the generalized Procrustes distance [51] between all three tracings simultaneously, for each participant, where the tracings have been resampled at 100 equally spaced horizontal points to allow comparison. Across HPPs, the inter-tracing correlations are extremely high (*r* ≥ 0.94) and the Euclidean and Procrustes distances are very small. Moreover, there are no significant differences between the three replication tracings across HPPs (all paired *t*-tests are not significant) and the correlations between tracings are positive (and mostly significant). This suggests that there are no systematic differences between tracings and that the between-tracings errors are due to the objective difficulty of landmarking. Therefore, the tracing process is very reliable, with a mean correlation between tracings close to 1.0.

**Table 2:** Inter-tracing reliability. The ID is the unique identifier of a HPP. The pairs of tracings are denoted as 1–2, 2–3 and 1–3, respectively. *r* is the Pearson’s correlation, *d*_*E*_ the Euclidean distance, *d*_*P*_ the (ordinary) Procrustes distance between pairs of tracings, and *d*_*Pgen*_ the generalized Procrustes distance between all three replication tracings simultaneously. For 100 discretization steps, each between 0.0 and 1.0, the maximum possible Euclidean distance is $\sqrt{100\cdot {\left(1.0-0.0\right)}^{2}}=10.0$. The bottom part of the table (*italic*, below the line) gives the means across HPPs.

ID	*r*			*d*_*E*_			*d*_*P*_			*d*_*Pgen*_
	1–2	2–3	1–3	1–2	2–3	1–3	1–2	2–3	1–3	
A03	1.00	0.99	1.00	0.18	0.42	0.35	0.02	0.03	0.03	0.04
A04	0.95	0.98	0.99	1.14	0.74	0.55	0.10	0.06	0.05	0.10
A05	0.99	0.98	1.00	0.57	0.74	0.41	0.05	0.07	0.04	0.07
A06	1.00	0.99	0.98	0.32	0.75	0.78	0.03	0.05	0.06	0.06
A09	0.98	0.99	0.98	0.62	0.66	0.88	0.06	0.05	0.07	0.08
A10	0.98	0.98	0.98	0.73	0.75	0.72	0.07	0.07	0.07	0.09
A11	0.91	0.99	0.88	1.47	0.40	1.71	0.13	0.04	0.15	0.16
A12	0.99	1.00	0.98	0.69	0.60	0.68	0.05	0.03	0.06	0.06
A13	0.98	1.00	0.99	0.69	0.29	0.56	0.06	0.02	0.05	0.06
A14	0.99	0.99	0.99	0.46	0.44	0.70	0.03	0.04	0.04	0.05
A15	0.98	1.00	0.98	0.74	0.42	0.89	0.07	0.03	0.07	0.08
A16	0.99	1.00	0.99	0.56	0.33	0.46	0.05	0.03	0.04	0.05
A17	0.97	0.99	0.95	0.91	0.60	1.10	0.08	0.05	0.10	0.10
A18	0.99	0.99	0.99	0.55	0.57	0.60	0.05	0.04	0.04	0.06
A19	0.98	0.98	0.99	0.62	0.70	0.64	0.06	0.05	0.05	0.07
A20	0.99	0.99	0.98	0.61	0.51	0.66	0.05	0.04	0.06	0.07
A21	0.99	0.99	0.99	0.40	0.51	0.41	0.04	0.05	0.04	0.06
A22	0.98	0.98	1.00	0.82	0.75	0.32	0.07	0.06	0.03	0.07
A23	0.98	0.98	0.99	0.59	0.63	0.38	0.05	0.06	0.04	0.07
A24	0.99	0.99	0.99	0.61	0.42	0.63	0.04	0.04	0.05	0.06
A25	0.94	0.97	0.99	1.30	0.96	0.48	0.11	0.08	0.04	0.11
A26	0.98	0.99	0.99	0.67	0.44	0.56	0.06	0.04	0.05	0.07
A27	1.00	0.99	0.99	0.44	0.59	0.50	0.03	0.04	0.05	0.05
A28	0.98	0.99	0.99	0.97	0.59	0.50	0.07	0.04	0.04	0.07
A29	0.97	0.94	0.94	1.05	1.22	1.62	0.07	0.09	0.08	0.12
A30	0.98	0.99	0.99	0.75	0.51	0.59	0.06	0.04	0.04	0.06
A31	0.98	0.98	0.99	0.76	0.80	0.49	0.06	0.06	0.04	0.07
A32	1.00	0.99	0.99	0.30	0.47	0.46	0.03	0.04	0.04	0.05
A33	0.97	0.95	0.90	1.03	1.01	1.71	0.08	0.09	0.14	0.14
A34	0.99	0.99	0.97	0.54	0.73	1.00	0.05	0.04	0.07	0.07
A35	0.98	0.98	0.96	0.72	0.92	0.95	0.06	0.06	0.08	0.09
A36	0.95	0.97	0.87	1.09	0.90	1.73	0.10	0.08	0.16	0.15
A37	0.98	0.99	0.99	0.99	0.68	0.53	0.07	0.06	0.04	0.07
A38	0.99	0.98	0.99	0.42	0.72	0.63	0.04	0.05	0.05	0.06
A39	0.98	0.99	0.97	0.70	0.61	0.79	0.06	0.04	0.08	0.08
A40	0.99	1.00	0.99	0.48	0.26	0.44	0.04	0.02	0.04	0.05
A41	0.82	0.99	0.81	2.13	0.58	2.05	0.17	0.05	0.17	0.19
A42	0.99	0.99	0.98	0.48	0.50	0.76	0.05	0.04	0.07	0.07
A43	0.99	0.99	0.99	0.49	0.45	0.39	0.04	0.04	0.03	0.05
A44	0.99	0.99	0.99	0.66	0.49	0.74	0.05	0.05	0.05	0.07
A45	0.99	0.98	1.00	0.48	0.64	0.34	0.04	0.06	0.03	0.06
A46	0.97	0.98	0.99	0.82	0.90	0.52	0.08	0.06	0.04	0.08
A47	0.99	1.00	0.99	0.45	0.18	0.42	0.04	0.02	0.03	0.04
A48	0.97	0.98	0.99	0.86	0.92	0.60	0.07	0.06	0.05	0.08
A49	0.99	0.99	1.00	0.67	0.63	0.32	0.04	0.05	0.03	0.05
A50	0.99	0.97	0.97	0.50	0.82	0.84	0.05	0.07	0.08	0.09
A51	0.99	0.97	0.97	0.58	0.87	0.93	0.05	0.08	0.07	0.09
A52	0.99	0.99	0.99	0.66	0.50	0.59	0.04	0.05	0.04	0.06
A53	0.99	0.99	0.99	0.38	0.40	0.39	0.04	0.03	0.04	0.05
A54	0.99	0.98	0.98	0.68	0.73	0.73	0.04	0.07	0.06	0.07
A55	0.99	0.97	0.98	0.55	0.81	0.77	0.05	0.07	0.07	0.09
A56	0.99	0.98	0.97	0.79	0.79	0.91	0.05	0.07	0.08	0.08
A57	0.98	0.99	0.99	0.77	0.47	0.49	0.07	0.04	0.04	0.07
A58	0.97	0.97	0.98	0.80	0.83	0.82	0.08	0.07	0.07	0.10
A59	0.99	0.99	1.00	0.67	0.63	0.33	0.05	0.05	0.03	0.06
A60	0.99	0.94	0.97	0.82	1.42	0.83	0.05	0.11	0.07	0.11
A61	0.99	0.99	0.99	0.37	0.65	0.65	0.03	0.05	0.05	0.06
A62	0.99	0.97	0.97	0.51	0.95	0.77	0.04	0.08	0.07	0.08
A63	1.00	1.00	1.00	0.26	0.19	0.33	0.02	0.02	0.03	0.03
A64	0.99	0.95	0.96	0.38	1.01	0.95	0.03	0.09	0.09	0.10
A65	0.99	0.99	0.99	0.52	0.59	0.61	0.05	0.04	0.05	0.06
A66	0.98	0.99	0.98	0.76	0.48	0.63	0.05	0.04	0.05	0.06
A67	1.00	0.99	0.99	0.29	0.51	0.42	0.02	0.04	0.04	0.04
A68	0.99	0.99	1.00	0.65	0.68	0.27	0.05	0.05	0.02	0.06
A69	1.00	0.99	1.00	0.46	0.67	0.33	0.03	0.05	0.03	0.05
A70	0.99	1.00	1.00	0.39	0.27	0.29	0.04	0.02	0.03	0.04
A71	0.99	0.96	0.99	0.68	1.03	0.55	0.05	0.09	0.05	0.08
A72	0.99	0.99	1.00	0.45	0.46	0.32	0.04	0.03	0.03	0.04
A73	1.00	1.00	0.99	0.41	0.31	0.49	0.04	0.03	0.05	0.05
A74	1.00	1.00	1.00	0.29	0.32	0.26	0.03	0.03	0.02	0.03
A75	0.99	0.99	0.99	0.44	0.37	0.45	0.03	0.04	0.04	0.05
A76	0.99	1.00	0.99	0.45	0.31	0.48	0.04	0.03	0.04	0.05
A77	0.99	0.99	0.98	0.47	0.47	0.57	0.04	0.04	0.05	0.06
A78	1.00	1.00	0.99	0.44	0.32	0.65	0.03	0.02	0.04	0.04
A79	0.96	0.96	0.92	1.24	0.98	1.83	0.08	0.08	0.12	0.13
A80	0.99	1.00	0.98	0.53	0.26	0.69	0.04	0.02	0.05	0.06
A81	0.98	0.98	1.00	0.83	0.75	0.26	0.06	0.06	0.02	0.06
A82	0.99	0.99	0.98	0.46	0.66	0.69	0.04	0.06	0.06	0.07
A83	0.96	0.98	0.99	0.85	0.68	0.50	0.07	0.05	0.04	0.08
A84	0.99	1.00	1.00	0.46	0.37	0.34	0.04	0.03	0.03	0.05
A85	1.00	1.00	1.00	0.35	0.36	0.23	0.03	0.03	0.02	0.03
A86	0.99	0.97	0.99	0.58	0.74	0.38	0.06	0.07	0.04	0.07
A87	0.99	0.98	0.98	0.49	0.91	0.73	0.04	0.07	0.06	0.07
A88	0.97	0.98	0.99	1.01	0.82	0.57	0.08	0.08	0.05	0.09
T01	0.99	0.95	0.97	0.39	1.05	0.77	0.03	0.10	0.07	0.09
T02	0.98	0.97	1.00	0.63	0.84	0.26	0.05	0.07	0.02	0.07
T03	0.99	0.94	0.97	0.55	1.24	0.91	0.05	0.10	0.08	0.10
T04	0.94	1.00	0.96	1.19	0.39	1.00	0.10	0.03	0.09	0.10
T05	1.00	0.99	0.99	0.27	0.63	0.64	0.03	0.05	0.05	0.06
T06	0.99	0.99	0.99	0.55	0.61	0.46	0.05	0.05	0.04	0.06
T07	0.95	0.99	0.95	1.15	0.66	1.16	0.11	0.04	0.10	0.11
T08	0.98	1.00	0.99	0.76	0.41	0.56	0.06	0.03	0.04	0.06
T09	0.99	1.00	0.99	0.56	0.43	0.40	0.04	0.02	0.03	0.04
T10	0.99	1.00	1.00	0.68	0.35	0.50	0.05	0.03	0.03	0.05
T11	1.00	0.99	0.98	0.32	0.59	0.83	0.02	0.05	0.07	0.07
T12	1.00	0.99	0.99	0.42	0.50	0.71	0.03	0.05	0.05	0.05
T13	0.98	0.98	1.00	0.85	0.66	0.39	0.07	0.06	0.03	0.07
T14	1.00	0.99	1.00	0.27	0.43	0.25	0.03	0.04	0.02	0.04
T15	0.99	0.99	0.99	0.44	0.61	0.45	0.04	0.04	0.04	0.05
T16	0.98	0.99	0.99	0.64	0.42	0.59	0.06	0.04	0.05	0.07
T17	0.98	0.98	0.99	0.69	0.62	0.60	0.06	0.06	0.05	0.07
T18	0.98	0.96	0.99	1.09	1.47	0.51	0.07	0.10	0.04	0.09
T19	0.97	0.95	0.99	0.88	1.15	0.42	0.08	0.10	0.03	0.09
T20	1.00	0.99	1.00	0.40	0.41	0.27	0.03	0.03	0.03	0.04
T21	1.00	0.94	0.94	0.41	1.23	1.19	0.02	0.11	0.11	0.11
T22	1.00	0.99	0.99	0.25	0.52	0.45	0.02	0.05	0.04	0.05
*Mean*	*0.98*	*0.98*	*0.98*	*0.64*	*0.64*	*0.65*	*0.05*	*0.05*	*0.05*	*0.07*

Because the Euclidean distances, Procrustes distances, and Pearson’s correlations, are extremely similar (Mantel correlations ≥ 0.94 in absolute value), we will focus here only on the Euclidean distances. We submitted them to a *k*-means clustering algorithm (R’s library fpc [70] function pamk, clustering around medioids and estimating the optimal number of clusters using the average silhouette width criterion [71]), and we found that the best number of clusters is *k* = 2. Moreover, the tracings of any given HPP tend to appear in the same cluster (for 91 out of 107 HPPs, or 85.0%, have all three tracings in the same cluster), confirming, in a different manner, that the tracing process is reliable.

**Fig 12:**
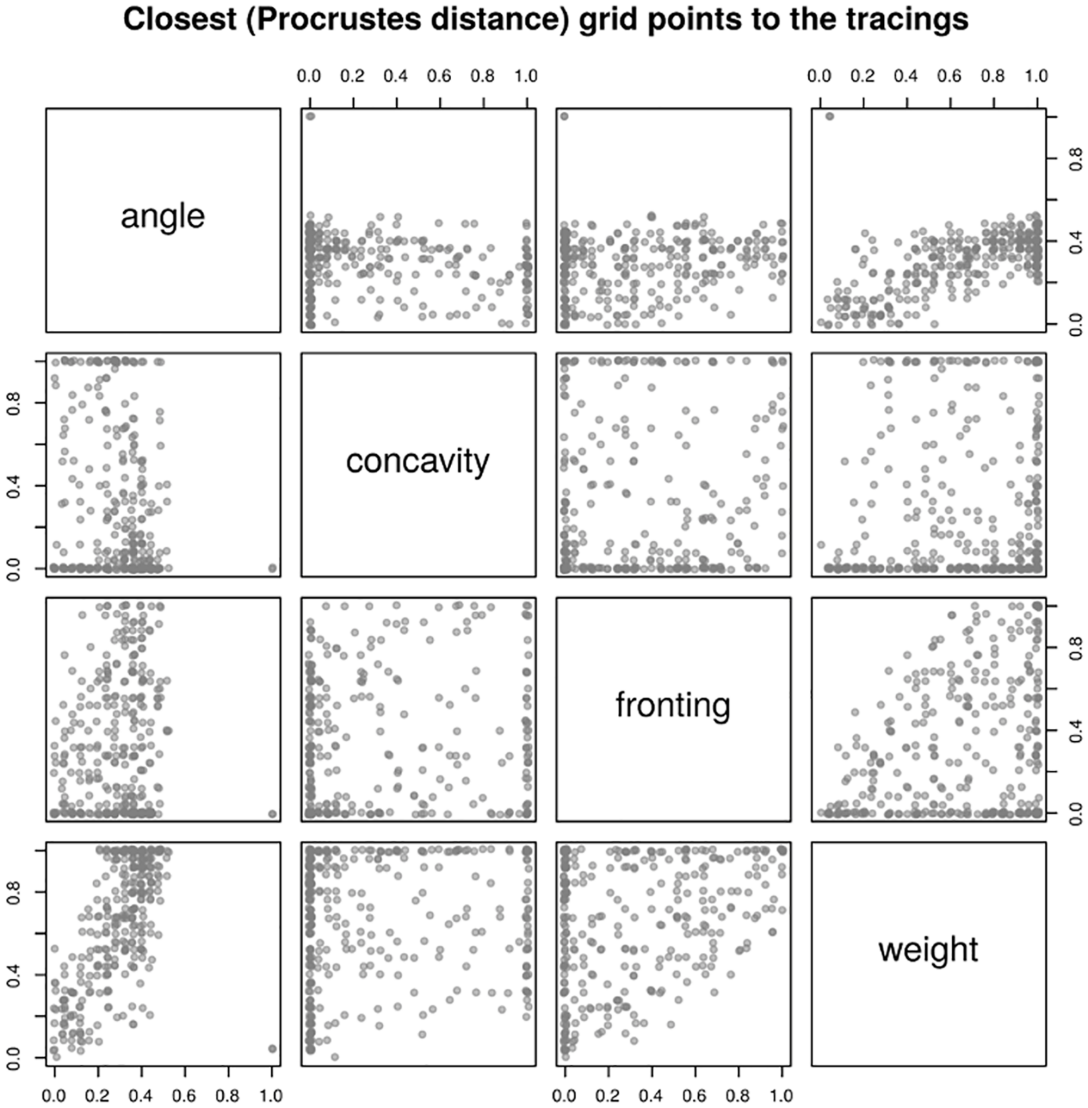
Closest (in terms of Procrustes distance) grid point to the tracings. Each dot represents the closest (in terms of minimizing the Procrustes distance) grid point to a tracing (the actual values have been jittered for better visualization). Each panel represents a 2D projection on two parameters of the 4-dimensional parameter space.

To understand the distribution of the actual variation in human HPPs, we computed the Procrustes distances between each tracing and each of the 456,976 systematically generated Bézier curves with 26 equally spaced parameter values (due to computational constraints, we downsampled from the full set of 6,765,201 51-equally spaced parameter values described in the **Materials and Methods**). Fig 12 plots for each tracing the closest grid point, showing that the tracings do not cover uniformly the whole parameter space.

**Fig 13:**
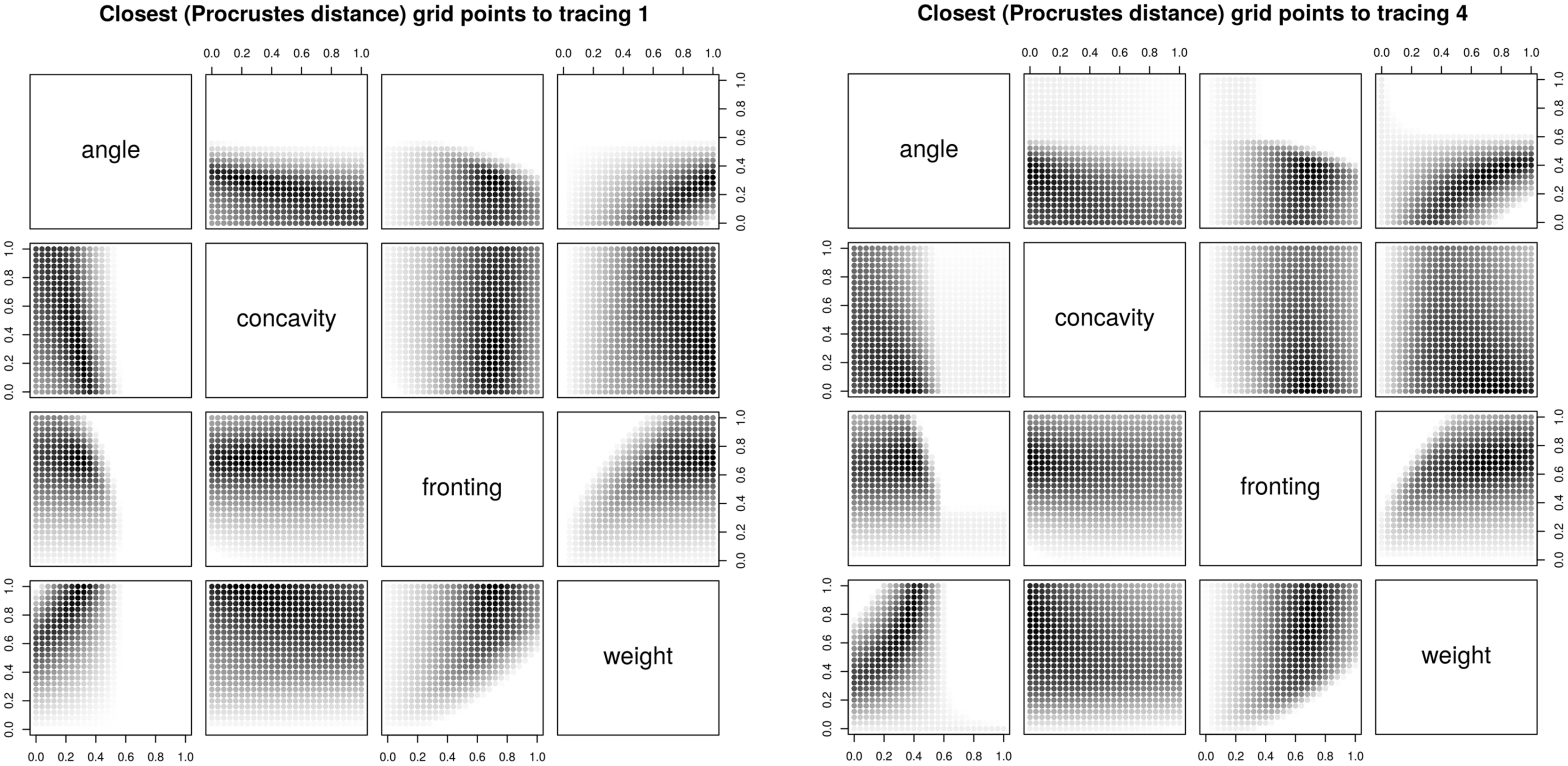
Closest (in terms of Procrustes distance) 100,000 grid points to two representative tracings. Each dot represents one of the top closest 100,000 grid points to tracing 1 (left panel) and tracing 4 (right panel, respectively), the darker the color the closer it being to the tracing. Each panel represents a 2D projection on two parameters of the 4-dimensional parameter space.

The analysis of individual tracings shows that the structure of the parameter space is smooth but non-trivial (Fig 13 shows two representative cases), and that the three tracings of the same HPP are highly similar (not shown). Thus, actual human hard palates are non-randomly distributed in the parameter space, apparently more clustered than expected (a similar picture emerges from the distribution of the Bézier fit parameters discussed below).

### Goodness of fit and parameter values across replications

Across all tracings and conditions, the Bézier curves fit the data much better than expected by chance: the one-sample *t*-tests comparing the *MSE*s of the actual HPP curves with *MSE*s of the randomly generated curves show the first are all significantly lower than the second.

How do the 100 independent replications relate to each other? Do all replications result in similar *MSE* and parameter estimates, suggesting that there is a unique “best-fitting” Bézier curve given by the set of parameter values, or are there multiple such sets? If the latter, are the *MSE*s comparable across these sets, indicating that there might be multiple equally good “best-fitting” Bézier curves, or are the *MSE*s different for different parameter values, suggesting that the “fitness landscape” is very complex and the GA becomes stuck in different local optima?

As expected, the different conditions strongly affect the answers, and we must analyze each of the 16 conditions separately; the details are in S1 Text, and we only present here summaries of the results.

#### In the fully-free condition (“”)

With all four parameters free to vary, there is a lot of variation between the 100 replications. For all HPPs, the *MSE*s are significantly different between the three tracings (one-way ANOVAs), and for most HPPs the variances in *MSE*s are significantly different as well (pair-wise Fligner-Killeen test with Bonferroni correction). The *MSE*s’ standard deviations within each tracing and HPP are very small (see Table 3). All four free parameters show big and significant differences between tracings within HPPs, and large spreads between the replications within tracings, except for “fronting”.

**Table 3:** Mean standard deviation (across the three replication tracings) of the goodness of fit (*MSE*) and parameter values for each condition.

condition	*MSE*	angle	conc	fronting	weight
“”	3.5e-05	0.031	0.03	0.0049	0.043
a	2.2e-06	–	0.0063	0.0015	0.0018
c	4.9e-06	0.0074	–	0.0012	0.0081
f	1.2e-05	0.0083	0.012	–	0.011
w	4.8e-06	0.002	0.011	0.0018	–
ac	1.5e-08	–	–	0.00029	0.00027
af	6.1e-08	–	0.0012	–	0.00027
aw	3.7e-08	–	0.00058	0.00013	–
cf	3e-08	0.00042	–	–	0.00058
cw	2.7e-08	0.00021	–	0.00037	–
fw	1.3e-07	0.00032	0.0018	–	–
acf	1.5e-10	–	–	–	1e-05
acw	3.1e-09	–	–	8.8e-06	–
afw	1.4e-11	–	3.4e-06	–	–
cfw	4.5e-10	1.1e-05	–	–	–
acfw	0	–	–	–	–

A dash (–) denotes a fixed parameter in a condition.

For most HPPs, the parameter values estimated by the 100 replications within each of the three tracings tend to cover different regions of the parameter space (see S1 Text), and the region of the parameter space explored for a given HPP and tracing tends to be roughly linear.

The Mantel correlations between the pair-wise distances (Euclidean or Procrustes) between the tracings and the pair-wise Euclidean distances between fitted parameter values (Table 4) are all significant (*p* < 10^−4^ uncorrected for multiple comparisons) and range between 0.48 and 0.50 (mean 0.49) for the Euclidean distances, and 0.45 and 0.47 (mean 0.46) for the Procrustes distances, suggesting that the parameter estimates do preserve the relative relationships between tracings.

**Table 4:** The distribution of parameter estimates per condition.

Condition	Euclidean			Procrustes		
	min	mean	max	min	mean	max
“”	0.48	0.49	0.50	0.45	0.46	0.47
a	0.56	0.56	0.56	0.54	0.54	0.54
c	0.59	0.60	0.60	0.56	0.57	0.57
f	0.40	0.41	0.42	0.36	0.38	0.39
w	0.52	0.52	0.54	0.51	0.51	0.52
ac	0.71	0.71	0.71	0.72	0.72	0.72
af	0.55	0.55	0.55	0.52	0.52	0.52
aw	0.69	0.69	0.69	0.71	0.72	0.72
cf	0.39	0.39	0.39	0.33	0.33	0.33
cw	0.68	0.68	0.68	0.71	0.71	0.71
fw	0.46	0.46	0.47	0.44	0.44	0.45
acf	0.85	0.85	0.85	0.83	0.83	0.83
acw	0.88	0.88	0.88	0.90	0.90	0.90
afw	0.59	0.59	0.59	0.58	0.58	0.58
cfw	0.88	0.88	0.88	0.87	0.87	0.87

The first column gives the conditions (the fully fixed condition “acfw” is missing as there are no differences between replications), the next three give the minimum, mean and maximum of the Mantel correlations between the original Euclidean distances between tracings and the Euclidean distances between parameter estimates, while the next three columns give the same information for the Procrustes distances between tracings and the Euclidean distances between parameter estimates. All Mantel correlations were computed with 1000 permutations and are significant at the 0.01 *α*-level (no multiple testing correction).

The Nearest Neighbor (nn) and mean distances suggest that the actual parameter estimates are more clustered than expected (*p* < 10^−4^), a finding also supported by the generalized Ripley’s $\hat{k}$ function, which shows that there the HPPs are not randomly distributed in the parameter space, being clustered at larger lags and dispersed at smaller lags.

Taken together, these results show that there are strong and (mostly) linear trade-offs between the four free parameters in the sense that, for a given tracing, the 100 optimally fitting parameter values are non-randomly distributed in the parameter space, suggesting the existence of ridges of equal fitness, resulting in multiple approximately equally well-fitting Bézier curves for a given tracing. The fitted parameter values tend to conserve the distances between the original tracings, reinforcing the validity of our fitting method.

Therefore, we expect that fixing some of these four parameters may not adversely affect the goodness of fit and might, in fact, reduce the equivalently good regions of the parameter space. The plots and summaries for all 16 conditions are in S1 Text and in Tables 3 and 4, but, in brief, we have the following results.

#### For the conditions with one fixed parameter (“a”, “c”, “f” and “w”)

The 100 replications are much more consistent, both in terms of their goodness of fit (*MSE*) and free parameter estimates, tending to form neat clusters within HPPs. The *MSE* and parameter estimates differ between most tracings and HPPs, but their variances are smaller than for the fully-free condition “”, with condition “f” showing slightly more variance in parameter estimates than the other three. The parameter estimates are clustered and preserve the relationships between the original tracings slightly better than for the fully-free condition, and best for condition “c”.

#### For the conditions with two fixed parameters (“ac”, “af”, “aw”, “cf”, “cw” and “fw”)

The estimates and goodness of fit become very tight and the 100 replications cover basically the same spot in the parameter space. This is confirmed by the strong tendency to form three clear-cut clusters of replications corresponding to the tracings, suggesting the fit is precise enough to detect the subtle differences between tracings. The parameter estimates are clustered and preserve the relationship between the original tracings, best for conditions “ac”, “aw” and “cw”.

#### For the conditions with three fixed parameters (“acw”, “afw” and “cfw”)

The parameter estimates still preserve the relationship between the original tracings, best for “acw”, but the clustering of the parameter estimates is not as clear-cut as before.

#### When all parameters are fixed (“acfw”)

The goodness of fit and parameter estimates per participant and tracing are (as expected) completely fixed.

### Which condition(s) fit the real data best?

The previous section shows that there are differences in how well different conditions fit the data, which, coupled with considerations of computational costs associated with the fitting process, raise the important question concerning how to choose the one (or more) condition(s) that, in some sense, fit the data “best”.

At the coarsest level, allowing all four parameters (“angle”, “concavity”, “fronting” and “weight”) to vary (i.e., the fully-free condition “”) results in a wide (but patterned) dispersion of the 100 replications in the parameter space. Moreover, the tightness of the goodness of fit and of the parameter estimates increases with less free parameters. These are due to the subtle dependencies between the parameters describing our model.

**Fig 14:**
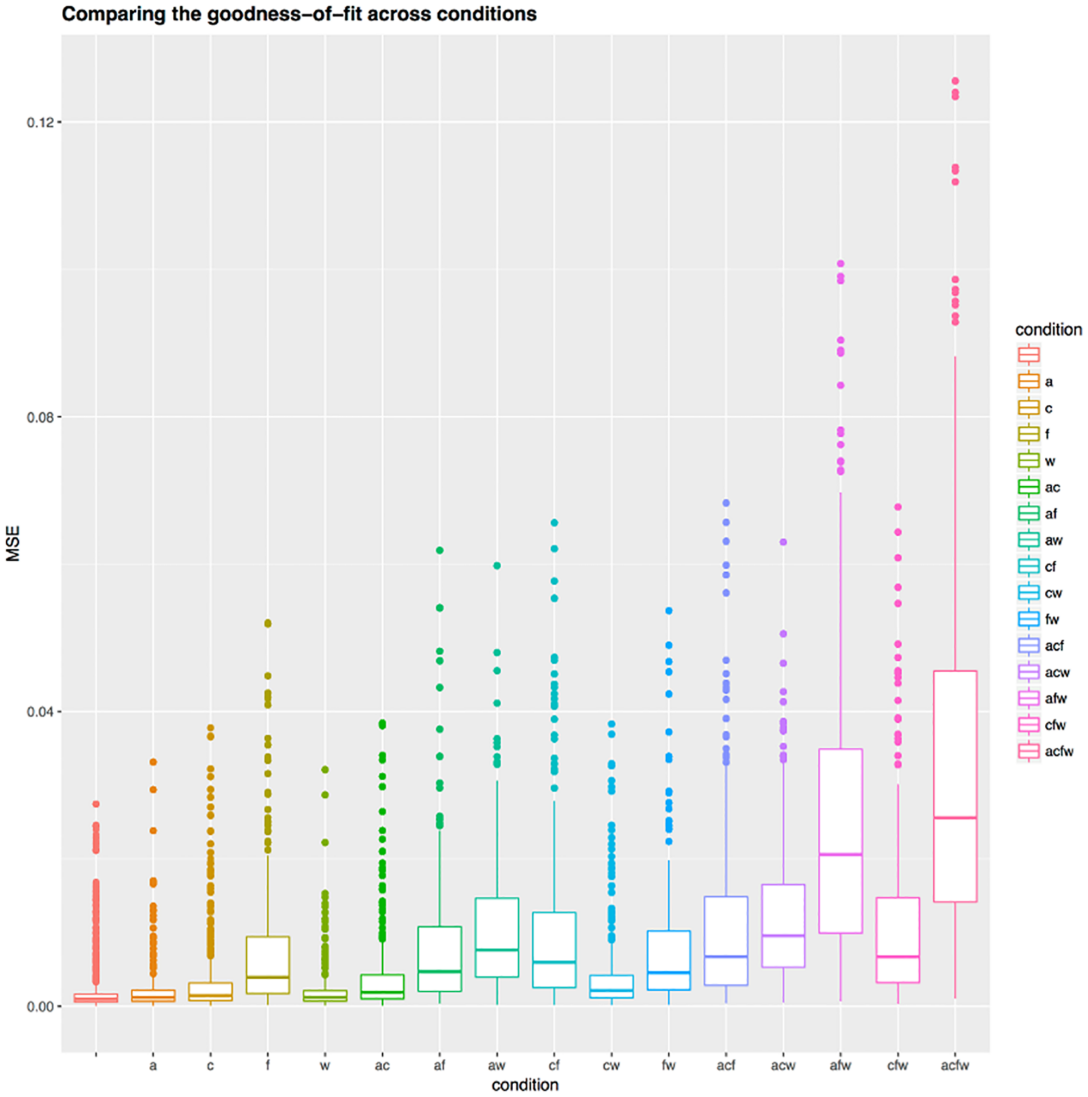
Comparing the goodness of fit across conditions. The distribution of goodness of fit (*MSE*) across conditions (identified both on the horizontal axis and by color) represented as boxplots.

**Fig 15:**
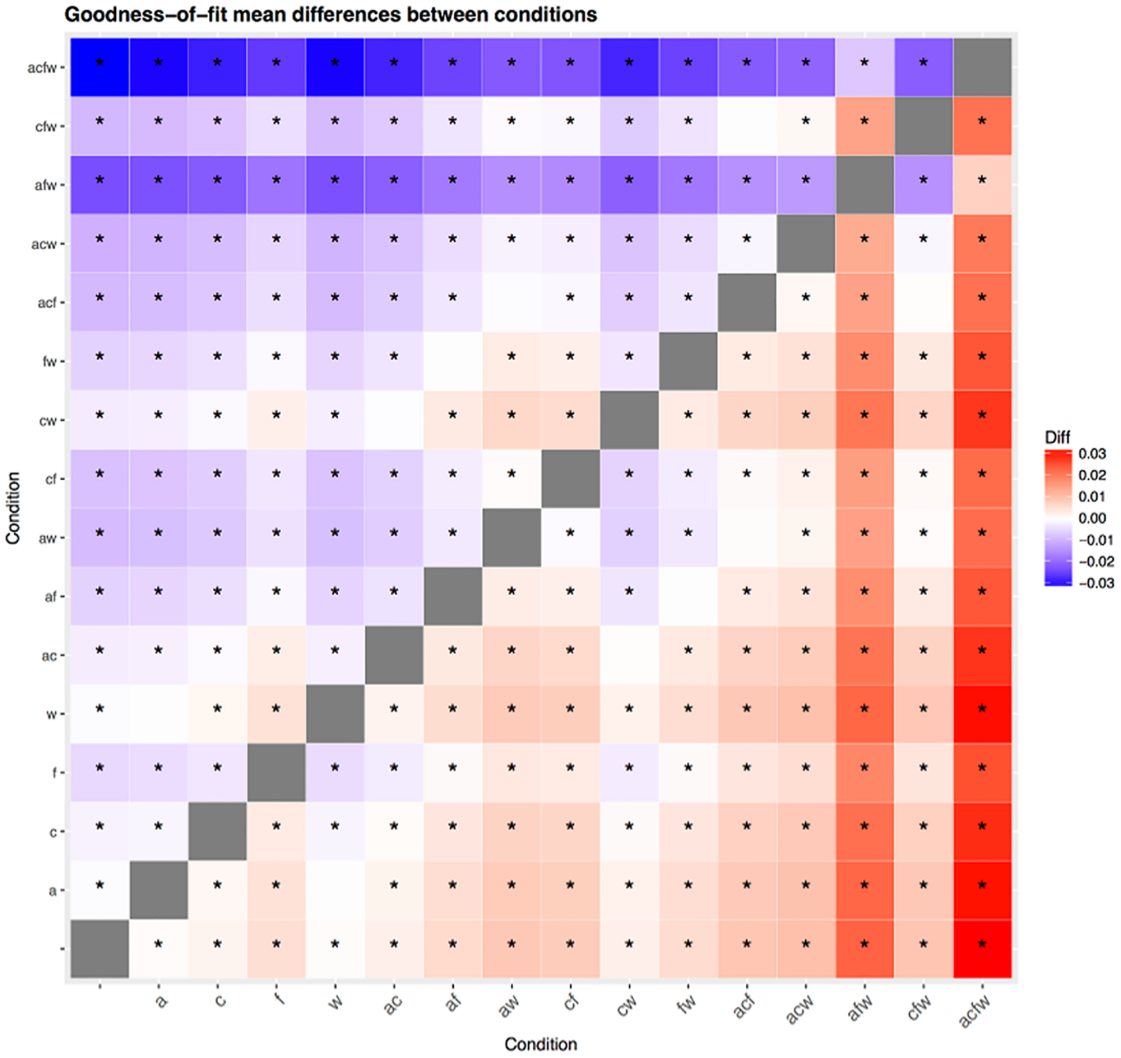
Difference in goodness of fit between conditions. This symmetric matrix represents the difference in mean goodness of fit (*MSE*) between all pairs of conditions (in column—row format) as color, varying between no difference (white) and maximum difference (positive red, negative blue); a star * means that the cell represents a significant difference in goodness of fit between the two conditions at the *α*-level of 0.01 after Tukey’s HSD posthoc testing correction.

Comparing the goodness of fit (*MSE*) across conditions (Figs 14 and 15 and Table 5) shows that the worst fit happens for the fully-fixed condition “acfw”, followed by some of the three-fixed parameters conditions. More precisely, the fully-free condition “” has overall the best fit, significantly better (at *α*-level 0.01 after Tukey’s multiple testing correction) than all the other conditions. However, fixing one parameter results in only a very slight worsening of the fit, while fixing two parameters results in conditions “af”, “aw”, “cf” and “fw” forming a block of similar fits, and “ac” and “cw” forming a second block of similar fits that are only slightly worse than the one-free-parameter conditions “a”, “c” and “w”. The three- and four-fixed parameter conditions result in much worse fits than the one-fixed parameter conditions.

**Table 5:** The mean and standard deviation of the goodness of fit per condition.

Condition	mean(*MSE*)	sd(*MSE*)
“”	0.0018	0.0031
a	0.0023	0.0037
c	0.0036	0.0057
f	0.0069	0.008
w	0.0022	0.0035
ac	0.0042	0.0061
af	0.0078	0.009
aw	0.011	0.0093
cf	0.01	0.012
cw	0.0044	0.0062
fw	0.0077	0.0085
acf	0.011	0.012
acw	0.012	0.0099
afw	0.026	0.021
cfw	0.011	0.012
acfw	0.033	0.026

**Fig 16:**
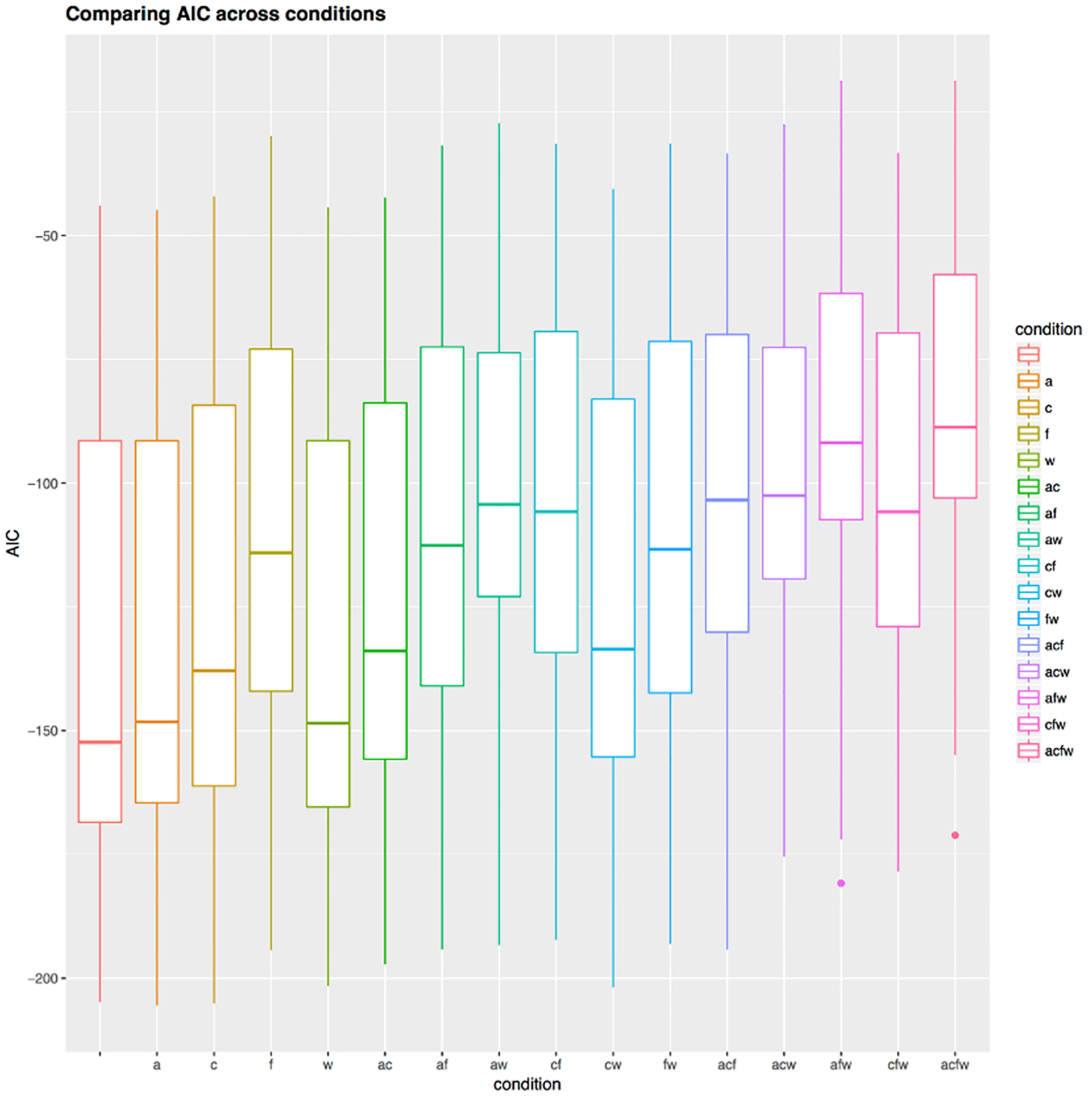
Comparing the AIC across conditions. The distribution of Akaike’s Information Criterion (*AIC*) across conditions (identified both on the horizontal axis and by color) represented as boxplots; lower *AIC* values are preferred.

**Fig 17:**
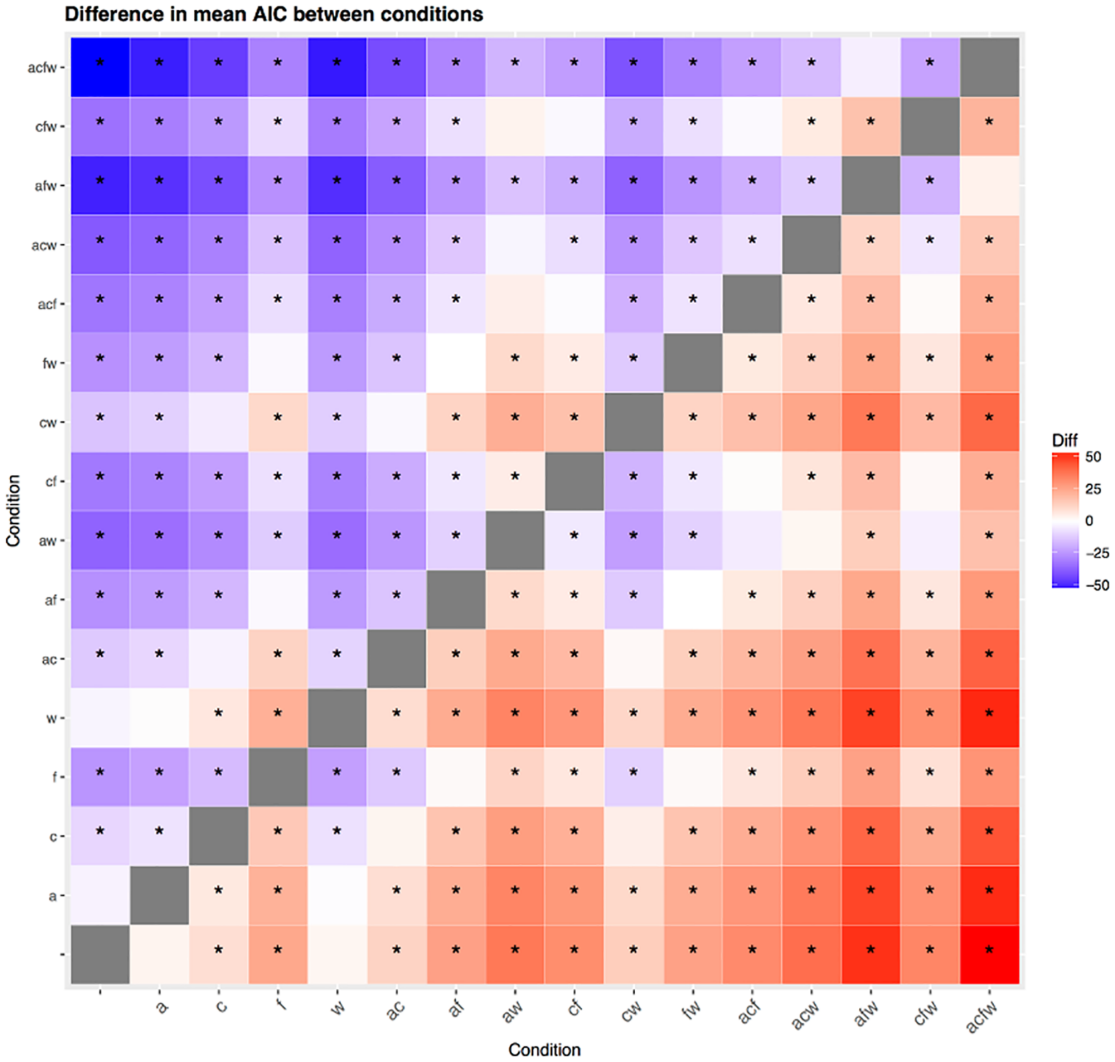
Difference in *AIC* between conditions. This symmetric matrix represents the difference in Akaike’s Information Criterion (*AIC*) between all pairs of conditions (same conventions as in Fig 15) as color, varying between no difference (white) and maximum difference (positive red, negative blue); a star * means that the cell represents a significant difference in *AIC* between the two conditions (i.e., the difference is bigger than 5 *AIC* points).

Figs 16 and 17 show that when considering the Akaike Information Criterion, *AIC*, (the pattern is very similar for *BIC*), the fully-free condition “” is not better (i.e., its *AIC* score differs by less than 5 *AIC* points) than the conditions with one fixed parameter “a” and “w”, but that it is indeed much better than all the other conditions. The two-fixed parameters conditions “ac” and “cw”, while worse than “a” and “w” (and, as an observation, obtained from these by fixing “c”), are nevertheless comparable to the “c” condition.

All these results together allow us to make the following recommendation:

- if the computational costs are the limiting factor, then the “ac” or “cw” conditions might be chosen, otherwise
- “a” or “w” are equally good choices and should be preferred to all other conditions.

### Fitting data: Bézier versus PCA

**Fig 18:**
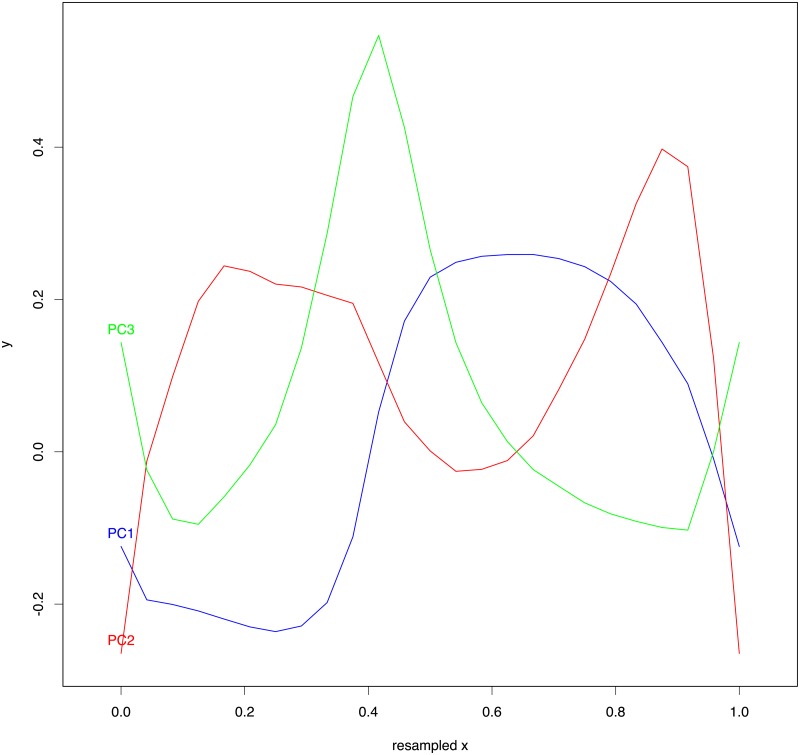
The first three Principal Components. The first three PCs, PC_1_—PC_3_, resulting from fitting the normalized and resampled (at 25 equally spaced horizontal points) 90 tracings.

**Fig 19:**
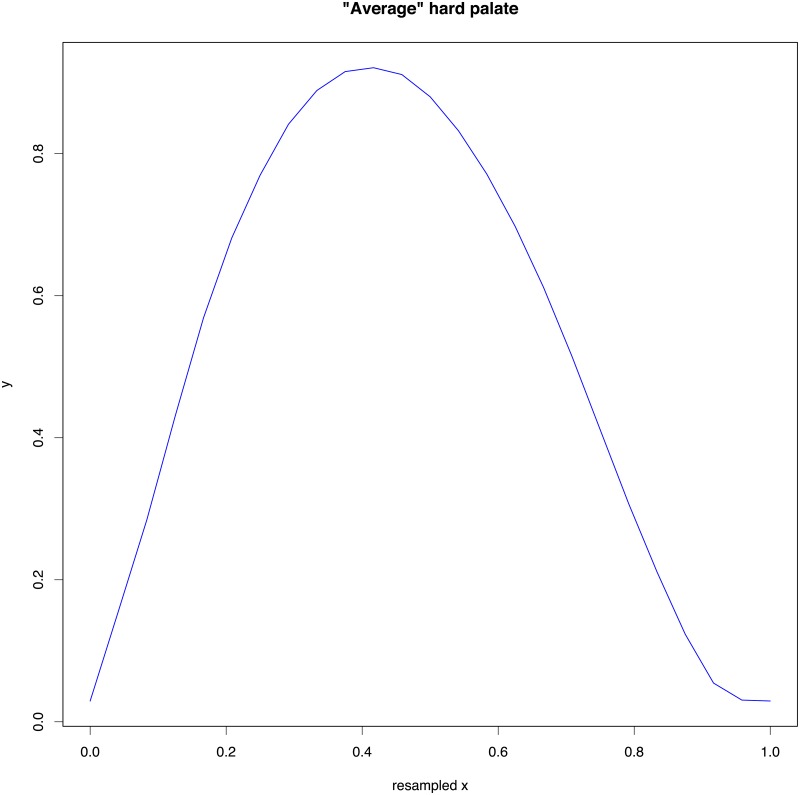
The “average” hard palate. The “average” hard palate reconstructed using the first three PCs, PC_1_—PC_3_, resulting from fitting the 90 normalized and resampled (at 25 equally spaced horizontal points) tracings.

**Fig 20:**
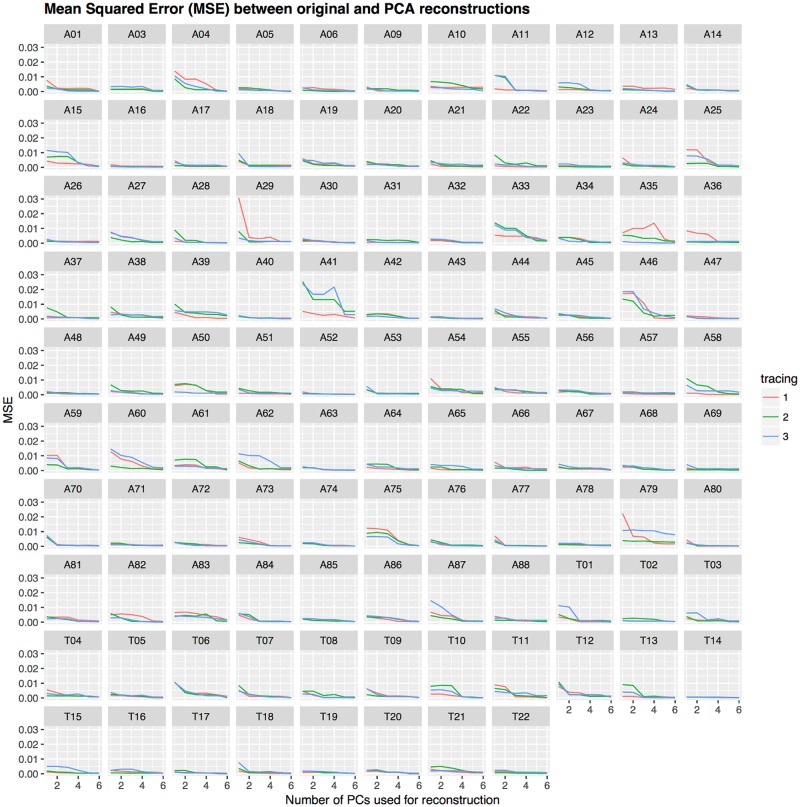
Relationship between goodness-of-fit and number of Principal Components. The vertical axis shows the goodness of fit (*MSE*) of the reconstructed tracings to the actual tracings when using a given number of PCs (the horizontal axis), across HPPs (the panels) and tracings (colors).

We conducted PCA on the resampled tracings (at 25 equally spaced horizontal positions) and we found that the first PC, PC_1_, explains most of the variance (52.7%), followed by PC_2_ (14.2%), and PC_3_ (11.6%). Fig 18 gives a visual representation of the first three PCs: PC_1_ represents a hard palate in very broad outlines with an accent on the anterior part (the alveolar ridge to the right), PC_2_ modulates this general outline in the front and dome parts, and PC_3_ further modifies the shape of the region immediately behind the alveolar ridge. Fig 19 shows the “average” hard palate obtained using the mean loadings across all participants on the first three PCs. These reconstructed tracings using the first 3 PCs are quite accurate across HPPs and tracings, as shown in Fig 20.

**Fig 21:**
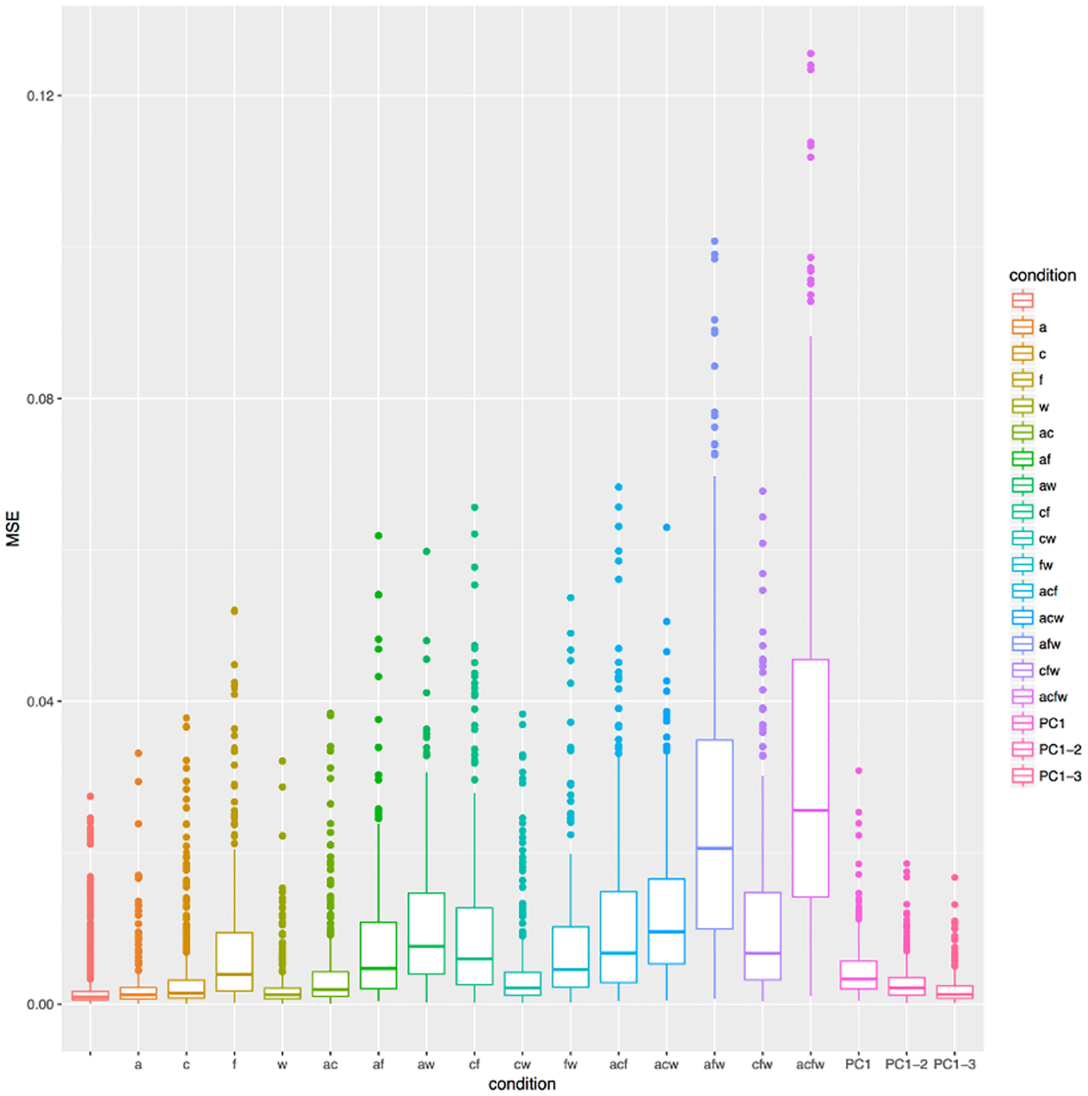
Comparing the goodness of fit of PCA and Bézier-based methods. The first 16 boxplots represent the conditions for the Bézier curve fitting method, while the last three boxplots represent the PCA fitting method using the first PC, the first two PCs, and the three first PCs, respectively (the boxplots are also distinguished using colors). The vertical axis represents is the *MSE*.

Table 6 and Fig 21 compare the goodness of fit of the various Bézier conditions with that of PCA using the first, the first two, and the first three PCs, respectively. As expected, how well the PCA fit the data depends on the number of PCs (degrees of freedom) used, with better fits for more PCs. Comparing the fit of the Bézier method with the PCA, we found that using only the first PC (thus, allowing only one degree of freedom) fits similarly to the Bézier conditions with two fixed parameters “ac” and “cw” (and two degrees of freedom), using the first two PCs (two degrees of freedom) is similar to the one fixed parameter condition “c” (three degrees of freedom), and using the first three PCs (three degrees of freedom) is equivalent to the Bézier conditions with one fixed parameter “a” and “w” (three degrees of freedom).

**Table 6:** Comparing the goodness of fit of PCA and Bézier-based methods.

Cond	mean(*MSE*)	1 PC	2 PCs	3 PCs
a	0.0023	-49.1%*	-23.9%*	9.4%
c	0.0036	-19.6%*	20.2%*	72.7%*
f	0.0069	53.7%*	129.8%*	230.2%*
w	0.0022	-51.0%*	-26.8%*	5.2%
ac	0.0042	-7.0%	39.0%*	99.8%*
af	0.0078	73.8%*	159.8%*	273.3%*
aw	0.011	139.0%*	257.2%*	413.3%*
cf	0.01	127.7%*	240.3%*	389.0%*
cw	0.0044	-2.0%	46.5%*	110.5%*
fw	0.0077	71.2%*	155.9%*	267.8%*
acf	0.011	146.7%*	268.7%*	429.9%*
acw	0.012	176.4%*	313.2%*	493.8%*
afw	0.026	479.4%*	766.1%*	1144.7%*
cfw	0.011	150.7%*	274.8%*	438.6%*
acfw	0.033	643.8%*	1011.8%*	1497.8%*

This table compares the goodness of fit to the data (*MSE*) of the PCA-based and the Bézier-based methods when considering 1, 2 or 3 PCs. The first column gives the conditions, the second gives the mean *MSE* for the conditions; the next three columns give the percent difference (i.e., how much better or worse the Bezier fit is relative to the PCA fit, where 0 means equal, 10% means a ten percent better fit for PCA, while −10% means a ten percent better fit for the Bezier procedure), and a star * means that the Bonferroni-corrected *p*-values of independent samples *t*-tests comparing the distribution of *MSE* between the PCA and Bézier fits is significant at the *α*-level of 0.01.

Therefore, our Bézier method fits the data relatively well compared with PCA, but the PCA does have an advantage in the sense that for the same number of degrees of freedom it fits the data better.

## Discussion

Our method, based on Bézier curves, is primarily aimed at applications that need to *generate* plausible human midsagittal hard palate profiles. As an example, we are currently using it in the development of an agent-based computer model where computational agents have a realistic geometric model of the vocal tract (based on Peter Birkholz’s widely-used VocalTractLab [72], http://www.vocaltractlab.de, denoted in the following as VTL) that is used to produce actual speech sounds by controlling various parameters of VTL such as tongue tip position or lower jaw aperture. We use the Bézier model to define agents with particular, well-defined hard palate anatomy, so that we can systematically investigate the influence of anatomical variation on speech production, with applications in speech pathology, but also in understanding normal inter-individual phonetic variation and even cross-cultural phonetic and phonological diversity [73]. With the Bézier model, we can specify the hard palate shape either (a) manually, through an interactive interface allowing the real-time modification of the four parameters and the visualization of the resulting Bézier curve, (b) by fitting a real hard palate shape from MRI data, or (c) by encoding the four parameters in the “genome” of an evolutionary algorithm allowing the investigation of inheritance, genetic drift and selection on hard palate shape. We have also developed a preliminary extension of this model to three dimensions using a parabolic description of the coronal shape of the hard palate, and we have integrated it into the biomechanical modeling system ArtiSynth [52] (www.artisynth.org), allowing us to explore the influence of hard palate shape on articulatory biomechanics. One application will be to refine the biomechanical modeling of click consonant articulation we have developed to study the influence of alveolar ridge on click production [74], replacing the current rather artificial manipulation of the alveolar ridge shape by the more adequate one offered by the Bézier curve approach.

Moreover, our method can also *fit* real human midsagittal hard palate shapes very well using only three (or even two) free parameters, to a level similar to (but slightly worse than) the widely-used Principal Component Analysis (PCA) method. While this is of secondary priority to us, it is nevertheless an important property of our method. First, it shows that indeed our method is appropriate for modeling human midsagittal hard palate profiles. Second, it summarizes real data in a small number (two or three) of meaningful parameters that can be used to statistically analyze patterns of anatomical variation.

There are other methods available, such as PCA, classic (CM) and geometric morphometrics (GM), and higher-order polynomials, that can be used to fit and, to some extent, generate human midsagittal hard palate shapes, and each might be more appropriate in certain settings. Far from promoting our Bézier curves approach as the “silver bullet”, we recognize that in many application GM is the preferred solution as it separates shape from size in a principled way, while CM is widely used and vast amounts of data are available using such descriptions. However, we have shown that the Bézier curves-based approach introduced here is particularly well-suited for computational approaches and might also be a useful way of summarizing existing anatomical variation using more interpretable parameters.

## Supporting information

S1 Dataset This XZ-compressed TAB-separated file contains the tracings (3 per HPP) in the “long” format (i.e., the entries for the same tracing appear on consecutive rows). It contains: **ID** (the hard palate’s unique ID), **tracing** (the tracing attempt number, between 1 and 3), and **x** and **y** (the (x, y) coordinates of the consecutive tracing points for a givne tracing, from top to bottom).
(XZ) Click here for additional data file.

S2 Dataset Tracings and best fitting Bézier curves. This XZ-compressed TAB-separated file contains, for each HPP, tracing and replication (one such case per row), the tracings and best fitting Bézier curves, in the following format: **name** (participant ID), **sex** and **age** (participant characteristics), **tracing** (between 1 and 3), **replication** (between 0 and 99), **x.start**, **x.end**, **y.start**, **y.end** and **rotation** (each tracing’s original coordinates of the leftmost and rightmost points, and the rotation before normalization), **lndmk.x.01** to **lndmk.x.25** (the normalized x-axis coordinates of the tracing points, starting always at 0.0 and ending at 1.0; those not used in a particular tracing are NA), **generation** (the generation at which the best-fitting Bézier curve was found), **condition** (the fixed parameters as string of letters), **MSE** (the mean squared error of the fit between the Bézier curve and the actual tracing), **angle.fixed**, **conc.fixed**, **fronting.fixed** and **weigth.fixed** (redundant information of the fixed and free parameters, see column **condition** above), **angle**, **conc**, **fronting** and **weigth** (the actual values of the four parameters describing the Bézier curve that best fits the tracing in the current replication), **lndmk.y.orig.01** to **lndmk.y.orig.25** (the normalized y-axis coordinates of the tracing points corresponding to the **lndmk.x.01** to **lndmk.x.25** x-axis coordinates), **lndmk.y.estim.01** to **lndmk.y.estim.25** (the normalized y-axis coordinates of the best Bézier curve found for the tracing in the current replication corresponding to the **lndmk.x.01** to **lndmk.x.25** x-axis coordinates of the tracing points). Please note that in order to reduce the file size, we used a lower decimal accuracy, which might produce slightly different results from the ones reported in the paper.
(XZ) Click here for additional data file.

S1 Text Goodness of fit and parameter values across replications for all conditions. The file plots-for-all-conditions.pdf contains all the relevant plots for each of the 16 conditions.
(PDF)

S1 Script Interactive exploration of generated Bézier curves. This R script for RStudio uses library manipulate to interactively change the values of the four parameters “angle”, “concavity”, “fronting” and “weight” and to display in real-time the corresponding Bézier curve. Please note that the Python script given in S2 Script is more general and allows the generation on-the-fly of the Bézier curve, while this script only permits the visualization of the already generated Bézier curves. The script is given in the file interactive-script-bezier.R, released under a GPL v2 license (https://www.gnu.org/licenses/old-licenses/gpl-2.0.en.html); please see the script file for details and requirements.
(R) Click here for additional data file.

S2 Script The Bézier curve model. The interactive Python 2 script generates a Bézier curve for a given set of parameter values and also allows the visual exploration of the effects and meaning of the model parameters. Please note that this script is general and allows the generation on-the-fly of the Bézier curve, while the R script in S1 Script only permits the visualization of the already generated Bézier curves. The script is given in the file bezier-model.py, released under a GPL v2 license (https://www.gnu.org/licenses/old-licenses/gpl-2.0.en.html); please the script file for details and requirements.
(PY) Click here for additional data file.

## References

[1] Barbujani G, Colonna V. Human genome diversity: frequently asked questions. Trends in Genetics. 2010;26(7):285–95. doi: 10.1016/j.tig.2010.04.002 20471132 10.1016/j.tig.2010.04.002

[2] Durand C, Rappold GA. Height matters-from monogenic disorders to normal variation. Nat Rev Endocrinol. 2013;9(3):171–7. doi: 10.1038/nrendo.2012.251 23337954 10.1038/nrendo.2012.251

[3] Bates TC, Luciano M, Medland SE, Montgomery GW, Wright MJ, Martin NG. Genetic variance in a component of the language acquisition device: ROBO1 polymorphisms associated with phonological buffer deficits. Behav Genet. 2011 1;41(1):50–7. doi: 10.1007/s10519-010-9402-9 20949370 10.1007/s10519-010-9402-9

[4] Plomin R, Haworth CMA, Meaburn EL, Price TS, Davis OSP. Common DNA Markers Can Account for More Than Half of the Genetic Influence on Cognitive Abilities. Psychol Sci. 2013 4;24(4):562–8. doi: 10.1177/0956797612457952 23501967 10.1177/0956797612457952 PMC3652710

[5] Jobling MA, Hollox E, Hurles M, Kivisild T, Tyler-Smith C. Human evolutionary genetics (2nd edition). New York: Garland Science; 2013.

[6] Ramachandran S, Deshpande O, Roseman CC, Rosenberg NA, Feldman MW, Cavalli-Sforza LL. Support from the relationship of genetic and geographic distance in human populations for a serial founder effect originating in Africa. Proc Natl Acad Sci U S A. 2005;102:15942–7. doi: 10.1073/pnas.0507611102 16243969 10.1073/pnas.0507611102 PMC1276087

[7] Manica A, Amos W, Balloux F, Hanihara T. The effect of ancient population bottlenecks on human phenotypic variation. Nature. 2007;448(7151):346–8. doi: 10.1038/nature05951 17637668 10.1038/nature05951 PMC1978547

[8] Betti L, Balloux F, Amos W, Hanihara T, Manica A. Distance from Africa, not climate, explains within-population phenotypic diversity in humans. Proc Biol Sci. 2009;276(1658):809–14. doi: 10.1098/rspb.2008.1563 19129123 10.1098/rspb.2008.1563 PMC2664379

[9] Gick B, Wilson I, Derrick D. Articulatory Phonetics (1st edition). Malden, MA: Wiley-Blackwell; 2013.

[10] Praveen BN, Sunitha A, Sumona P, Shubhasini A, Syed V. Various Shapes of Soft Palate: A Lateral Cephalometric Study. World Journal of Dentistry. 2011;2:207–10. doi: 10.5005/jp-journals-10015-1084

[11] Byers SN, Churchill SE, Curran B. Identification of euro-Americans, Afro-Americans, and Amerindians from palatal dimensions. Journal of forensic sciences. 1997;42:3–9. doi: 10.1520/JFS14061J 8988568

[12] Ferrario VF, Sforza C, Dellavia C, Colombo A, Ferrari RP. Three-dimensional hard tissue palatal size and shape: a 10-year longitudinal evaluation in healthy adults. Int J Adult Orthodon Orthognath Surg. 2002;17(1):51–8. 11934056

[13] Fitch WT, Giedd J. Morphology and development of the human vocal tract: a study using magnetic resonance imaging. J Acoust Soc Am. 1999 9;106(3 Pt 1):1511–22. doi: 10.1121/1.427148 10489707 10.1121/1.427148

[14] Kumar DK, Gopal KS. Morphological Variants Of Soft Palate In Normal Individuals: A Digital Cephalometric Study. Journal of Clinical and Diagnostic Research. 2011;5(6):1310–3.

[15] Lammert A, Proctor M, Narayanan S. Morphological Variation in the Adult Vocal Tract: A Study Using rtMRI. In: Proceedings of the 9th International Seminar on Speech Production. 2011.

[16] Lammert A, Proctor M, Narayanan S. Morphological Variation in the Adult Hard Palate and Posterior Pharyngeal Wall. Journal of Speech, Language and Hearing Research. 2013;56:521–30. doi: 10.1044/1092-4388(2012/12-0059) 10.1044/1092-4388(2012/12-0059) PMC3885355 23690566

[17] You M, Li X, Wang H, Zhang J, Wu H, Liu Y, et al Morphological variety of the soft palate in normal individuals: a digital cephalometric study. Dentomaxillofac Radiol. 2008;37(6):344–9. doi: 10.1259/dmfr/55898096 18757720 10.1259/dmfr/55898096

[18] Harshman R, Ladefoged P, Goldstein L. Factor analysis of tongue shapes. J Acoust Soc Am. 1977;62(3):693–713. doi: 10.1121/1.381581 903511 10.1121/1.381581

[19] Vorperian HK, Kent RD, Gentry LR, Yandell BS. Magnetic resonance imaging procedures to study the concurrent anatomic development of vocal tract structures: preliminary results. International Journal of Pediatric Otorhinolaryngology. 1999 8;49(3):197–206. doi: 10.1016/S0165-5876(99)00208-6 10519699 10.1016/s0165-5876(99)00208-6

[20] Dellavia C, Sforza C, Orlando F, Ottolina P, Pregliasco F, Ferrario VF. Three-dimensional hard tissue palatal size and shape in Down syndrome subjects. The European Journal of Orthodontics. 2007;29(4):417–22. doi: 10.1093/ejo/cjm026 17702802 10.1093/ejo/cjm026

[21] Dixon MJ, Marazita ML, Beaty TH, Murray JC. Cleft lip and palate: synthesizing genetic and environmental influences. Nat Rev Genet. 2011;12(3):167–78. doi: 10.1038/nrg2933 21331089 10.1038/nrg2933 PMC3086810

[22] Patel SR, Frame JM, Larkin EK, Redline S. Heritability of upper airway dimensions derived using acoustic pharyngometry. Eur Respir J. 2008;32(5):1304–8. doi: 10.1183/09031936.00029808 18579548 10.1183/09031936.00029808 PMC2655306

[23] Reynoso MC, Hernández A, Soto F, García-Cruz O, Martínez y Martínez R, Cantú JM. Autosomal dominant macroglossia in two unrelated families. Hum Genet. 1986;74(2):200–2. doi: 10.1007/BF00282095 3770748 10.1007/BF00282095

[24] von Cramon-Taubadel N. Global human mandibular variation reflects differences in agricultural and hunter-gatherer subsistence strategies. PNAS. 2011;108(49):19546–51. doi: 10.1073/pnas.1113050108 22106280 10.1073/pnas.1113050108 PMC3241821

[25] Kaifu Y, Kasai K, Townsend GC, Richards LC. Tooth wear and the “design” of the human dentition: A perspective from evolutionary medicine. Am J Phys Anthropol. 2003 1 1;122(S37):47–61. doi: 10.1002/ajpa.10329 10.1002/ajpa.10329 14666533

[26] Bourdiol P, Mishellany-Dutour A, Abou-El-Karam S, Nicolas E, Woda A. Is the tongue position influenced by the palatal vault dimensions? J Oral Rehabil. 2010;37(2):100–6. doi: 10.1111/j.1365-2842.2009.02024.x 19925581 10.1111/j.1365-2842.2009.02024.x

[27] Brunner J, Fuchs S, Perrier P. The influence of the palate shape on articulatory token-to-token variability. ZAS Papers in Linguistics. 2005;42:43–67.

[28] Brunner J, Fuchs S, Perrier P. On the relationship between palate shape and articulatory behavior. J Acoust Soc Am. 2009;125(6):3936–49. doi: 10.1121/1.3125313 19507976 10.1121/1.3125313

[29] Hiki S, Itoh H. Influence Of Palate Shape On Lingual Articulation. Speech Communication. 1986;5:141–58. doi: 10.1016/0167-6393(86)90004-X

[30] Lammert A, Proctor M, Katsamanis A, Narayanan S. Morphological Variation in the Adult Vocal Tract: A Modeling Study of its Potential Acoustic Impact. In: Twelfth Annual Conference of the International Speech Communication Association. 2011. Available from: http://www.mproctor.net/docs/lammert11_IS2011_morphology.pdf

[31] Tiede MK, Boyce SE, Holland CK, Choe KA. A new taxonomy of American English /r/ using MRI and ultrasound. The Journal of the Acoustical Society of America. 2004;115(5):2633–4. doi: 10.1121/1.4784878

[32] Weirich M, Fuchs S. Vocal tract morphology can influence speaker specific realisations of phonemic contrasts. Proceedings of the ISSP. 2011;251–8. 10.1044/1092-4388(2013/12-0217) 24687445

[33] Weirich M. Articulatory and Acoustic Inter-Speaker Variability in the Production of German Vowels. ZAS Papers in Linguistics. 2010;52:19–42.

[34] Zhou X, Espy-Wilson CY, Tiede M, Boyce S. An articulatory and acoustic study of “retroflex” and “bunched” American English rhotic sound based on MRI. INTERSPEECH. 2007 p. 54–7. Available from: http://www.isr.umd.edu/Labs/SCL/publications/conference/t_zhou_etal_icslp_07.pdf 10.1121/1.2902168 PMC2680662 18537397

[35] Stanier P, Moore GE. Genetics of cleft lip and palate: syndromic genes contribute to the incidence of non-syndromic clefts. Hum Mol Genet. 2004;13 Spec No 1:R73–81. doi: 10.1093/hmg/ddh052 14722155 10.1093/hmg/ddh052

[36] Boehringer S, van der Lijn F, Liu F, Günther M, Sinigerova S, Nowak S, et al Genetic determination of human facial morphology: links between cleft-lips and normal variation. Eur J Hum Genet. 2011;19(11):1192–7. doi: 10.1038/ejhg.2011.110 21694738 10.1038/ejhg.2011.110 PMC3198142

[37] Bush JO, Jiang R. Palatogenesis: morphogenetic and molecular mechanisms of secondary palate development. Development. 2012;139(2):231–43. doi: 10.1242/dev.067082 22186724 10.1242/dev.067082 PMC3243091

[38] Leslie EJ, Marazita ML. Genetics of Cleft Lip and Cleft Palate. Am J Med Genet C Semin Med Genet. 2013;163(4):246–58. doi: 10.1002/ajmg.c.31381 10.1002/ajmg.c.31381 PMC3925974 24124047

[39] Online Mendelian Inheritance in Man, OMIM^®^. McKusick-Nathans Institute of Genetic Medicine, Johns Hopkins University (Baltimore, MD), 2015 URL: http://omim.org/

[40] Riquelme A, Green LJ. Palatal width, height, and length in human twins. Angle Orthod. 1970;40(2):71–9. 5266016 10.1043/0003-3219(1970)040<0071:PWHALI>2.0.CO;2

[41] Townsend GC, Richards LC, Sekikawa M, Brown T, Ozaki T. Variability of palatal dimensions in South Australian twins. J Forensic Odontostomatol. 1990;8(2):3–14. 2130047

[42] D’Souza AS, Mamatha H, Jyothi N. Morphometric analysis of hard palate in south Indian skulls. Biomedical Research. 2012;23(2):173–5.

[43] Hassanali J, Mwaniki D. Palatal analysis and osteology of the hard palate of the Kenyan African skulls. Anat Rec. 1984;209(2):273–80. doi: 10.1002/ar.1092090213 6465536 10.1002/ar.1092090213

[44] van Reenen JF, Allen DW. The palatal vault of the Bushman (San), Vassekela and Himba. J Dent Assoc S Afr. 1987;42(8):489–92. 3509645

[45] Winkler EM, Kirchengast S. Metric characters of the hard palate and their cephalometric correlations in Namibian! Kung San and Kenyan tribes. Hum Biol. 1993;65(1):139–50. 8436387

[46] Younes S, el Angbawi MF, al Dosari AM. A comparative study of palatal height in a Saudi and Egyptian population. J Oral Rehabil. 1995;22(5):391–5. doi: 10.1111/j.1365-2842.1995.tb00790.x 7616351 10.1111/j.1365-2842.1995.tb00790.x

[47] Brunner J, Hoole P, Perrier P, Fuchs S. Temporal development of compensation strategies for perturbed palate shape in German/sch/-production. In: Proceedings of the 7th International Seminar on Speech Production. 2006. p. 247–54. Available from: http://halshs.archives-ouvertes.fr/hal-00403289/

[48] Tiede M, Gracco V, Shiller D, Espy-Wilson S, Boyce CE. Perturbed palatal shape and North American English /r/ production. Journal of the Acoustical Society of America. 2005;117(4):2568–2569. doi: 10.1121/1.4788555

[49] Chovalopoulou M-E, Valakos ED, Manolis SK. Sex determination by three-dimensional geometric morphometrics of the palate and cranial base. Anthropol Anz. 2013;70(4):407–25. doi: 10.1127/0003-5548/2013/0363 24620567 10.1127/0003-5548/2013/0363

[50] Bugaighis I, O’Higgins P, Tiddeman B, Mattick C, Ali OB, Hobson R. Three-dimensional geometric morphometrics applied to the study of children with cleft lip and/or palate from the North East of England. The European Journal of Orthodontics. 2010;. doi: 10.1093/ejo/cjp140 20097701 10.1093/ejo/cjp140

[51] Zelditch ML, Swiderski DL, Sheets HD. Geometric Morphometrics for Biologists: A Primer. Academic Press; 2012.

[52] Lloyd JE, Stavness I, Fels SS. ArtiSynth: A fast interactive biomechanical modeling toolkit combining multibody and finite element simulation In Payan Y (Ed.) Soft tissue biomechanical modeling for computer assisted surgery (pp. 355–394). Berlin, Germany: Springer; 2012.

[53] MATLAB and Statistics Toolbox Release 2012b, The MathWorks, Inc., Natick, Massachusetts, United States. 2012.

[54] Python Software Foundation. Python Language Reference, version 2.7. Available at http://www.python.org

[55] R Core Team. R: A language and environment for statistical computing. R Foundation for Statistical Computing, Vienna, Austria. 2015. https://www.R-project.org/.

[56] Bézier Curve. Wolfram MathWorld. http://mathworld.wolfram.com/BezierCurve.html [Accessed Nov. 2015]

[57] Bézier Curve. Wikipedia. https://en.wikipedia.org/wiki/Bézier_curve [Accessed Nov. 2015]

[58] Farin G, Hoschek J, Kim M-S. Handbook of computer aided geometric design. Elsevier 2002

[59] RStudio Team. RStudio: Integrated Development for R. RStudio, Inc., Boston, MA; 2015. http://www.rstudio.com/.

[60] RStudio Team. manipulate: Interactive Plots for RStudio. R package version 0.98.1103; 2011.

[61] Banzhaf W, Nordin P, Keller RE, Francone FD. Genetic Programming: An Introduction. Morgan Kaufmann Publishers; 1997.

[62] Eiben A, Smith J. Introduction to evolutionary computing (Chapter: Genetic algorithm). Springer-Verlag, Berlin Heidelberg; 2003.

[63] Mantel N. The detection of disease clustering and a generalized regression approach. Cancer Res. 1967;27(2):209–20. 6018555

[64] Ian L. Dryden. shapes: Statistical Shape Analysis. R package version 1.1–11; 2015. http://CRAN.R-project.org/package=shapes.

[65] Schabenberger O, Gotway CA. Statistical Methods for Spatial Data Analysis. Boca Raton, Florida: Chapman & Hall/CRC Press; 2005.

[66] Dediu D, Levinson SC. Abstract Profiles of Structural Stability Point to Universal Tendencies, Family-Specific Factors, and Ancient Connections between Languages. Mesoudi A, editor. PLoS ONE. 2012;7(9):e45198 doi: 10.1371/journal.pone.0045198 23028843 10.1371/journal.pone.0045198 PMC3447929

[67] Burnham KP, Anderson DR. Model Selection and Multimodel Inference: A Practical Information-Theoretic Approach. Springer Science & Business Media; 2002.

[68] Panchal G, Ganatra A, Kosta YP, Panchal D. Searching Most Efficient Neural Network Architecture Using Akaike’s Information Criterion (AIC). International Journal of Computer Applications. 2010;1(5):41–4. doi: 10.5120/126-242

[69] Jolliffe IT. Principal Component Analysis. 2nd ed Springer Verlag: NY; 2002.

[70] Christian Hennig. fpc: Flexible Procedures for Clustering. R package version 2.1–10; 2015. http://CRAN.R-project.org/package=fpc.

[71] Rousseeuw P.J. Silhouettes: A graphical aid to the interpretation and validation of cluster analysis. J. Comput. Appl. Math., 20, 53–65; 1987 doi: 10.1016/0377-0427(87)90125-7

[72] Birkholz P. 3D-Artikulatorische Sprachsynthese. Logos Verlag, Berlin; 2005 http://www.vocaltractlab.de/publications/birkholz-2005-dissertation.pdf.

[73] Dediu D, Janssen R, Moisik SR. Language is not isolated from its wider environment: vocal tract influences on the evolution of speech and language. Language and Communication 2016;

[74] Moisik SR, Dediu D. Anatomical biasing and clicks: Evidence from biomechanical modeling. Journal of Language Evolution. in press;

