# Supplementary material for: Modelling human hard palate shape with Bézier curves
Source: PLoS One. 2018 Feb 15;13(2):e0191557. doi: 10.1371/journal.pone.0191557 (PMC5813942; doi:10.1371/journal.pone.0191557)
Supplement: S1 Text — The file plots-for-all-conditions.pdf contains all the relevant plots for each of the 16 conditions. (PDF) [file pone.0191557.s003.pdf]

Supplementary Information for "Modelling Human Hard Palate Shape with Bézier Curves": Goodness-of-fit and parameter values across replications for all conditions

Rick Janssen[1], Scott Moisik[2,1] and Dan Dediu[1,3,*]

[1]Language and Genetics Department, Max Planck Institute for Psycholinguistics, Nijmegen, The Netherlands
[2]Linguistics and Multilingual Studies, Nanyang Technological University, Singapore
[3]Donders Institute for Brain, Cognition and Behaviour, Nijmegen, The Netherlands
[*]Corresponding author: Dan.Dediu@mpi.nl

7th March 2017

This is part of the Supplementary Information for the paper "Modelling Human Hard Palate Shape with Bézier Curves" and contains the details concerning the relationship between replications for each of the 16 conditions (the fully free condition "" is reproduced here for easier comparison with the other conditions). The conventions for the figures are the same as in the main text for the fully-free "" condition, and we plot one figure per page. The conditions are grouped by the number of fixed parameters. *N.B.*: in these figures "participant" is a shorthand for what we called in the main text "hard palate profile" (HPP).

1 The condition with all parameetrs free (the fully-free condition, “”)

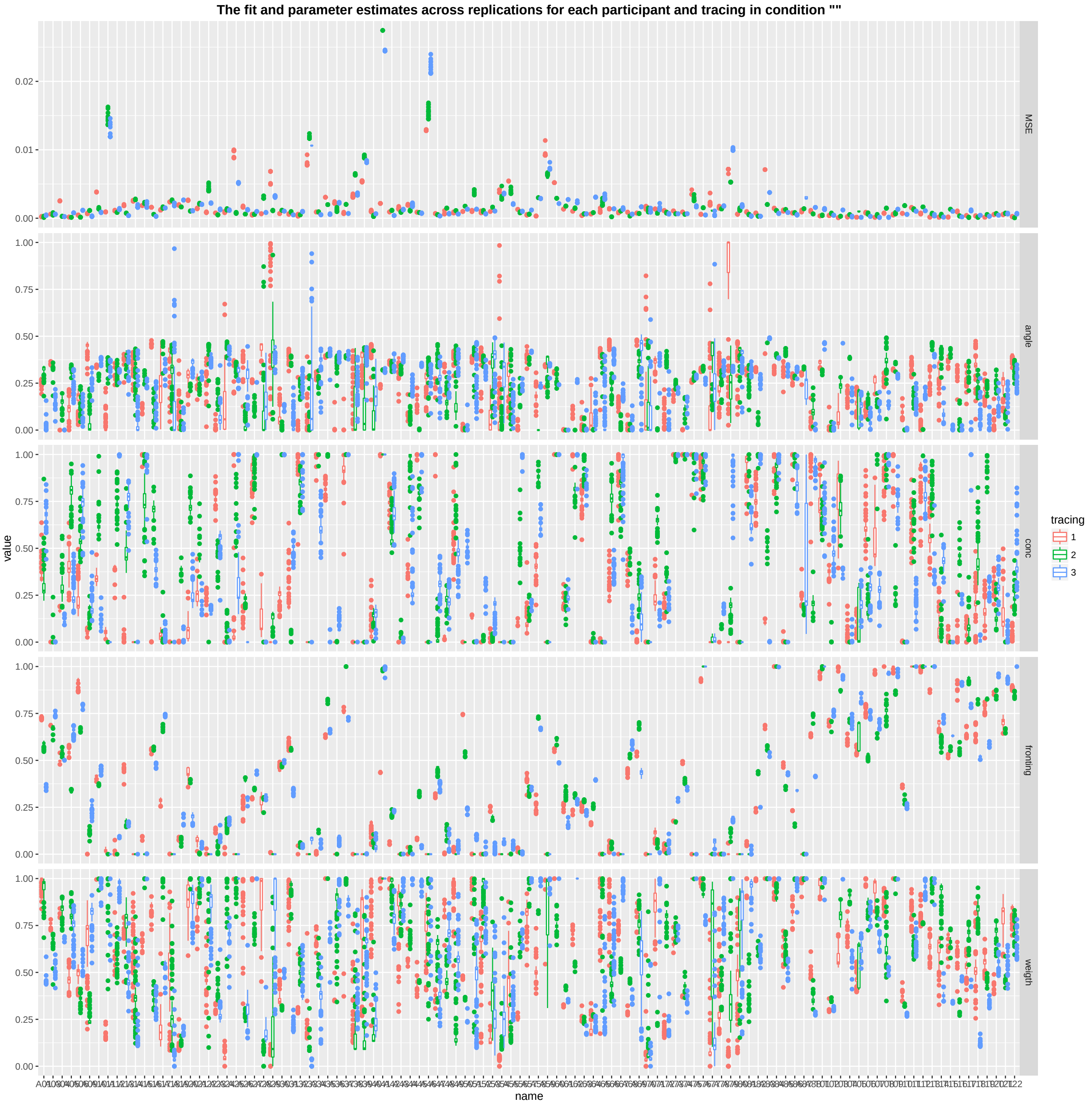

Figure 1: The fit and parameter estimates across the 100 replications for each participant and tracing in condition “”.

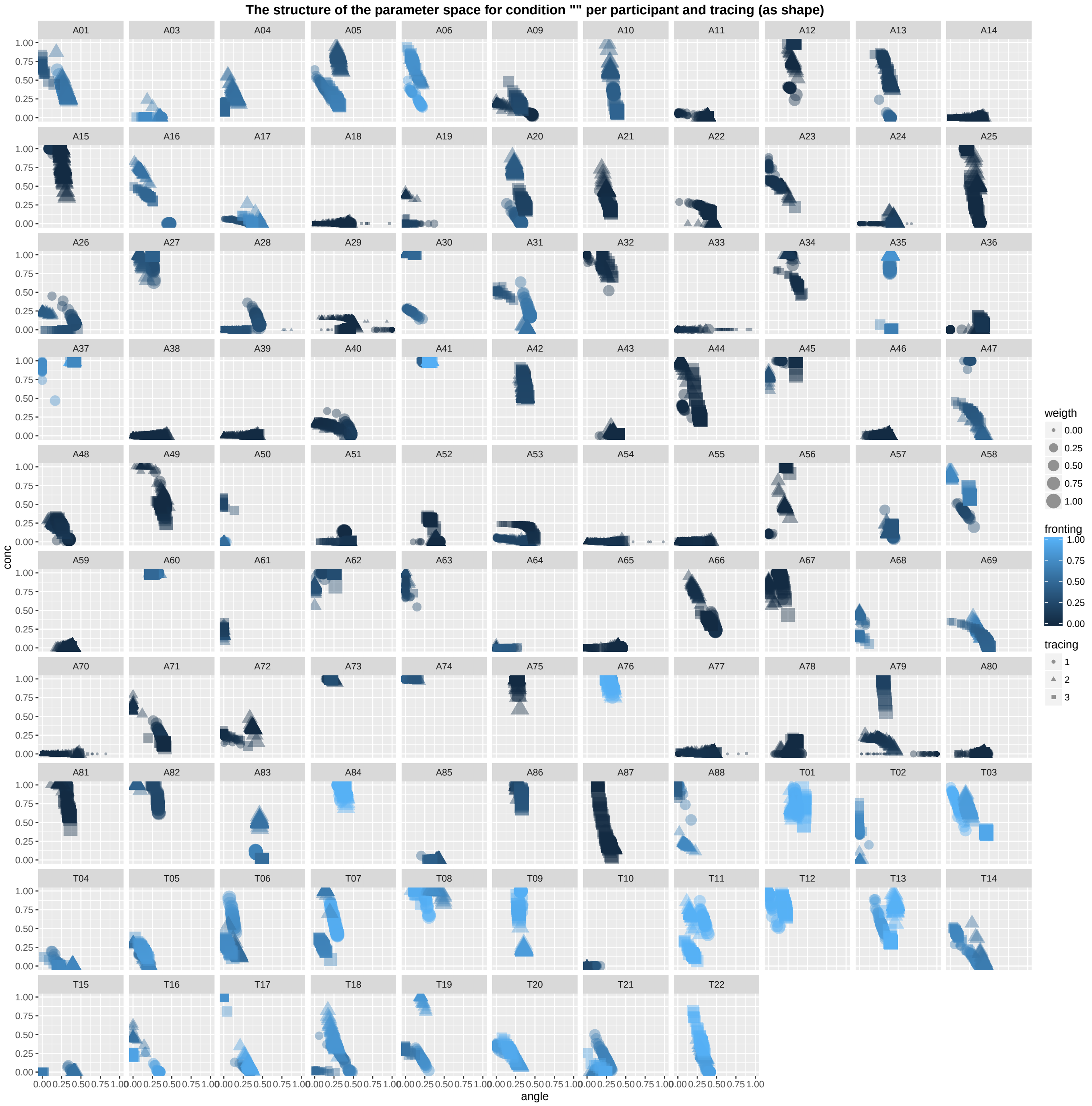

Figure 2: The parameter space across the 100 replications for each participant and tracing in condition “”.

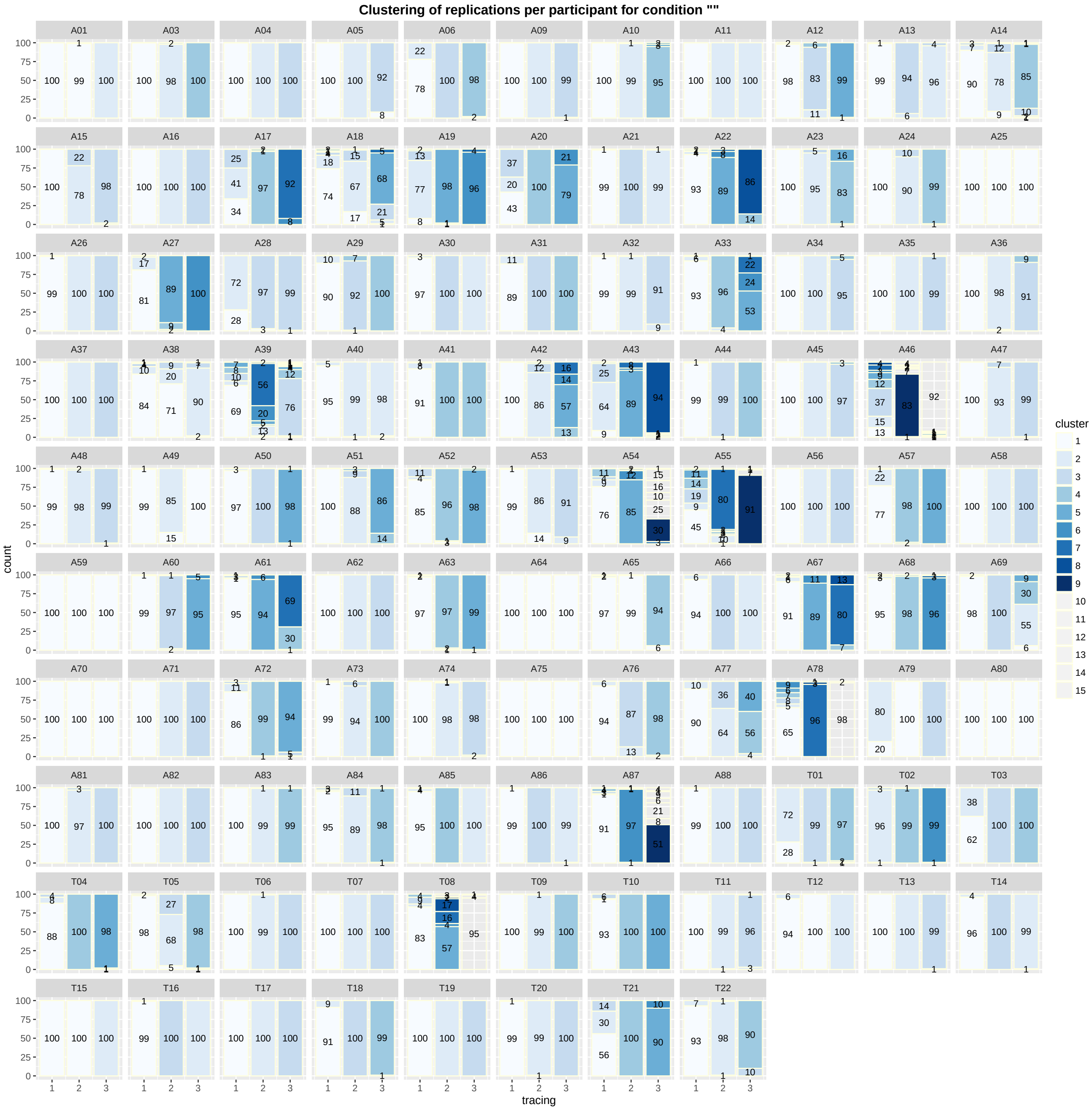

Figure 3: Clustering of replications for each participant and tracing in condition “” (some participants might be missing because there are no free parameters with non-constant estimates). Numbers give the number of replications in each cluster. Please note that the clusters are specific to each participant (i.e., cluster ”1” for participant ”X” is different than the cluster ”1” for participant ”Y”).

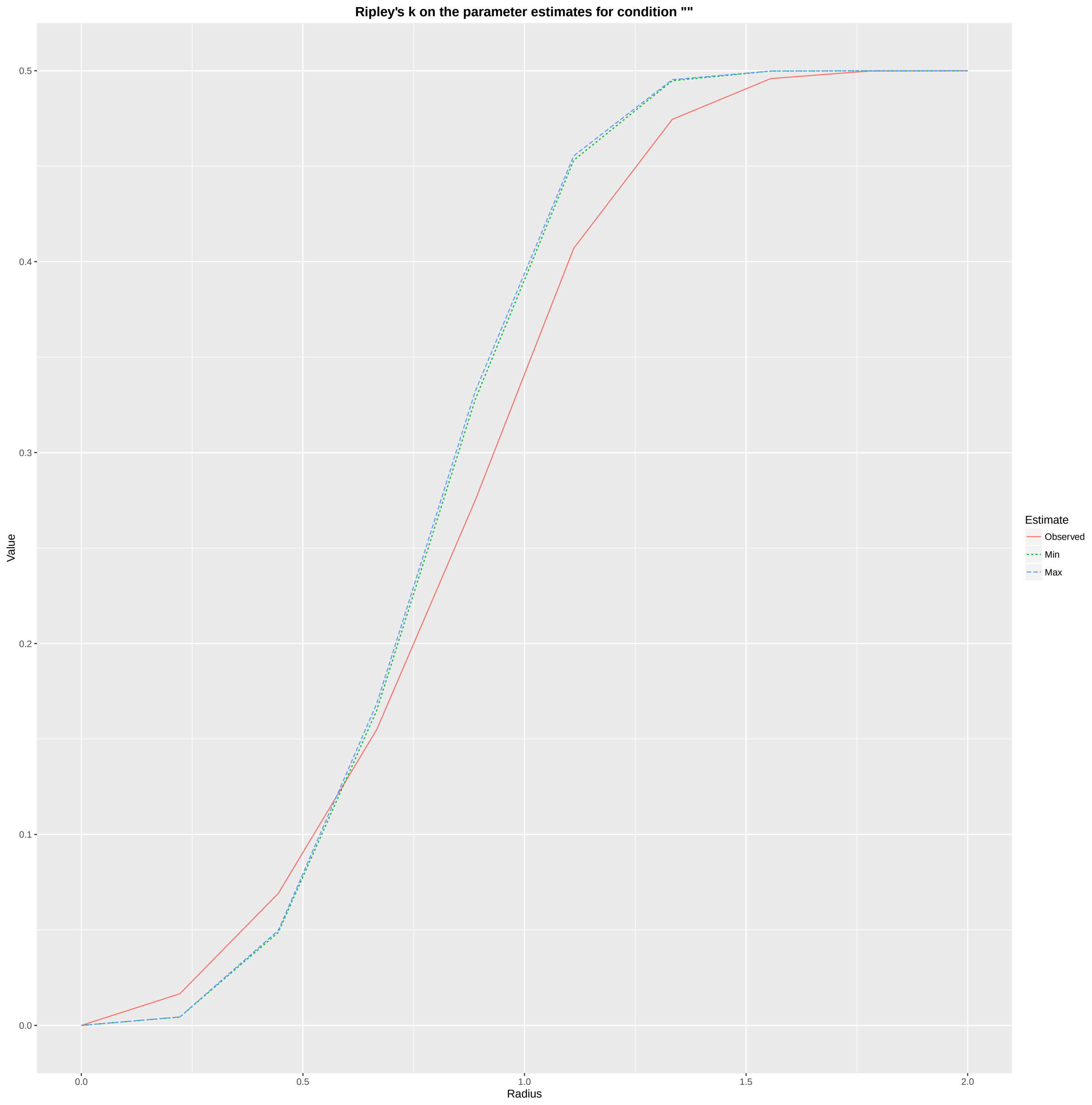

Figure 4: Generalized Ripley's $\hat{k}$ function for the parameter estimates (vertical axis) at 10 distance lags (horizontal axis), showing the actually observed values as well the minimum and maximum values for 100 randomly generated equivalent number of parameter estimates extracted from a Poisson distribution. If the observed $\hat{k}$ is higher than expected then there is significant clustering at that lag, while lower indicate dispersion; otherwise we cannot reject the hypothesis of spatial randomness.

2 Conditions with one fixed parameter (“a”,“c”,“f”,“w”)

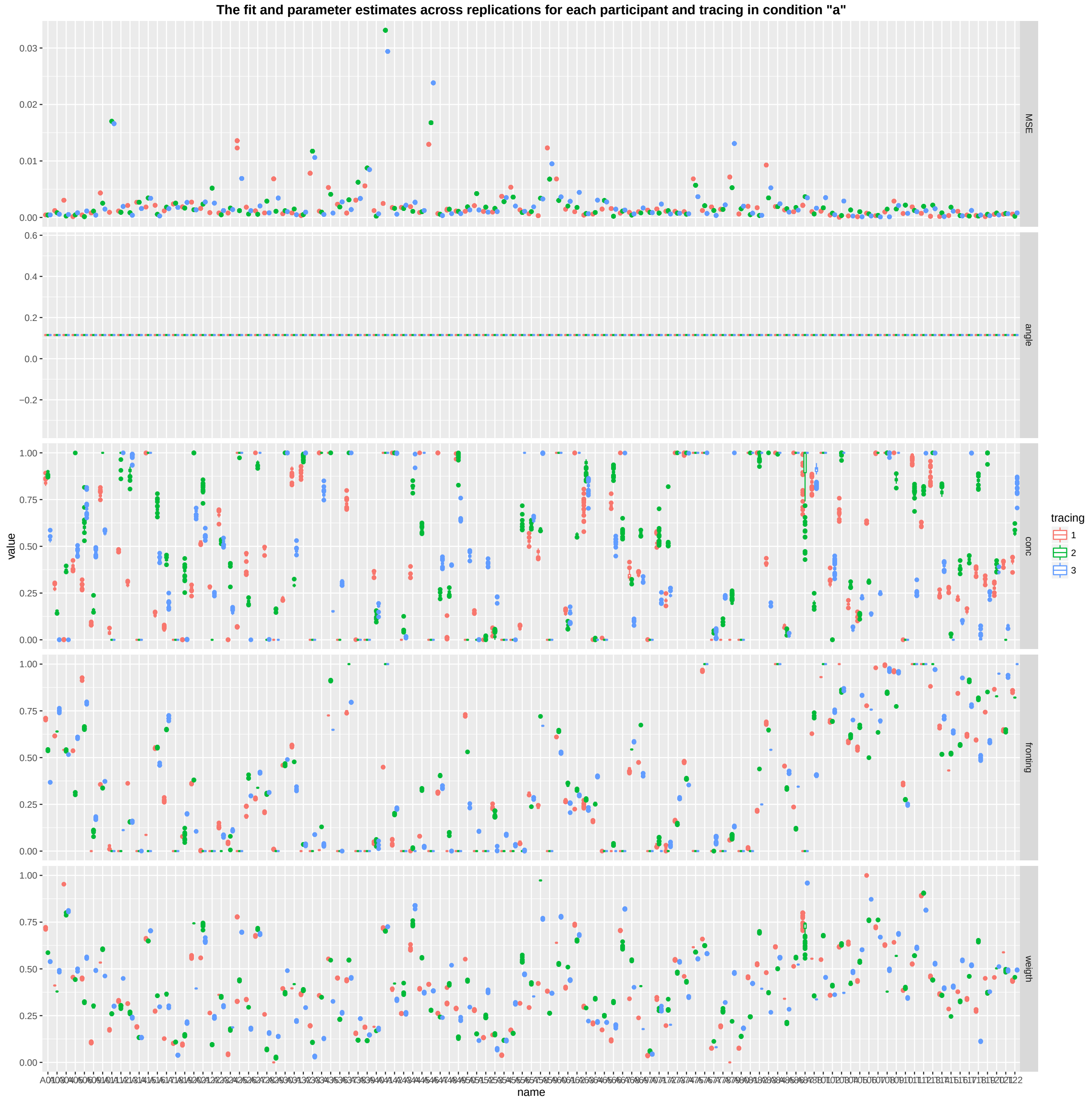

Figure 5: The fit and parameter estimates across the 100 replications for each participant and tracing in condition “a”.

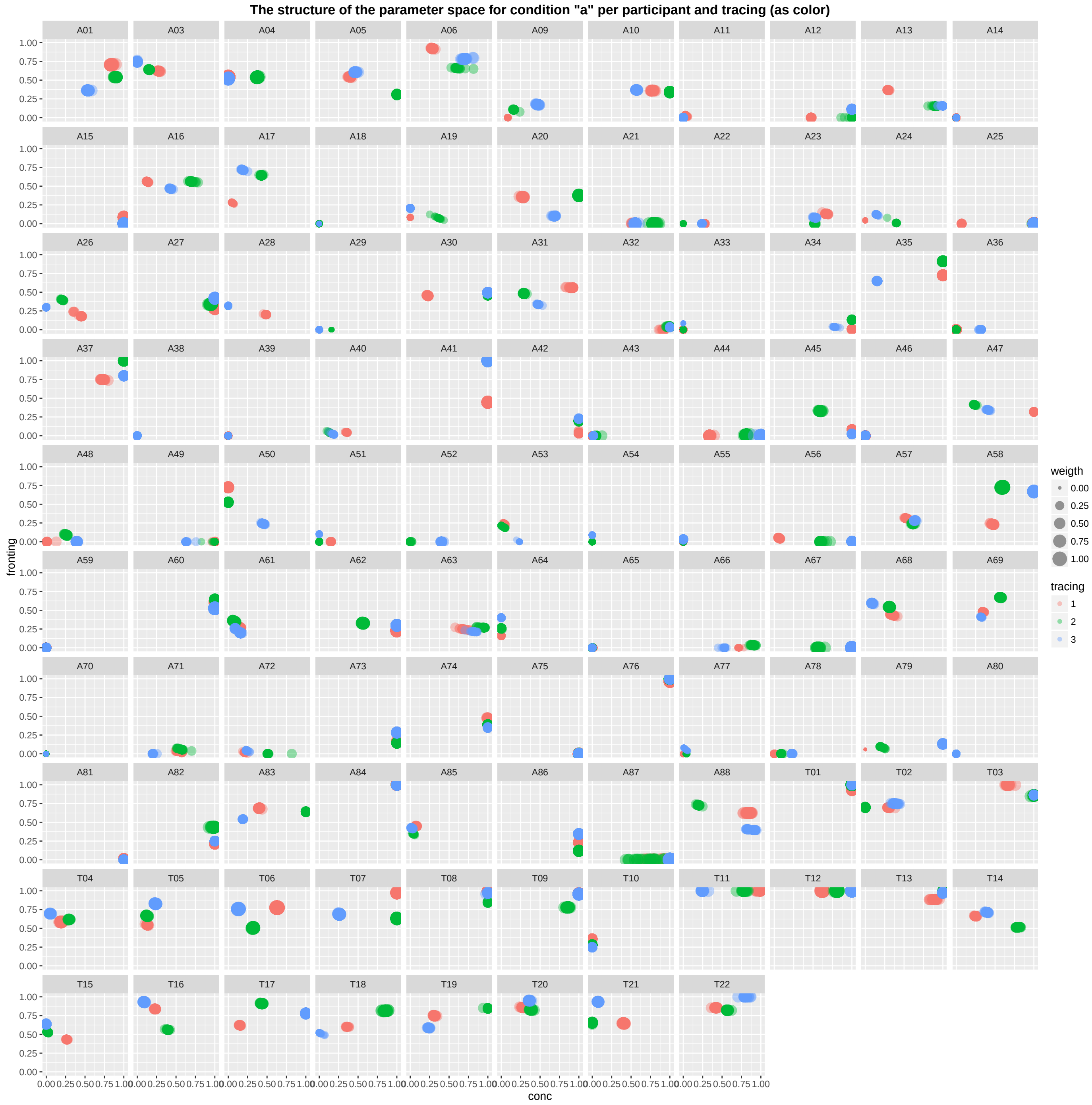

Figure 6: The parameter space across the 100 replications for each participant and tracing in condition "a".

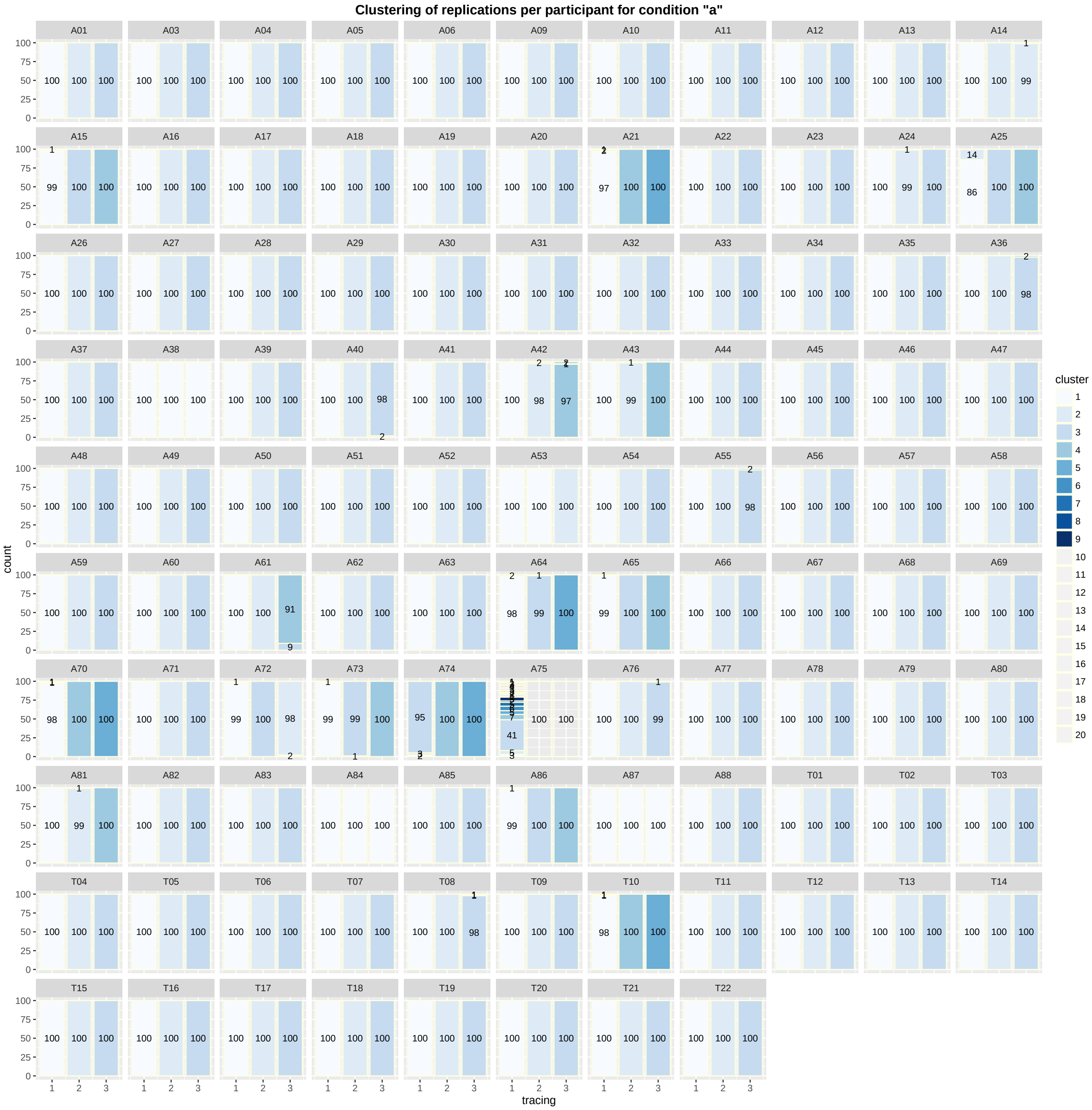

Figure 7: Clustering of replications for each participant and tracing in condition “a” (some participants might be missing because there are no free parameters with non-constant estimates). Numbers give the number of replications in each cluster. Please note that the clusters are specific to each participant (i.e., cluster ”1” for participant ”X” is different than the cluster ”1” for participant ”Y”).

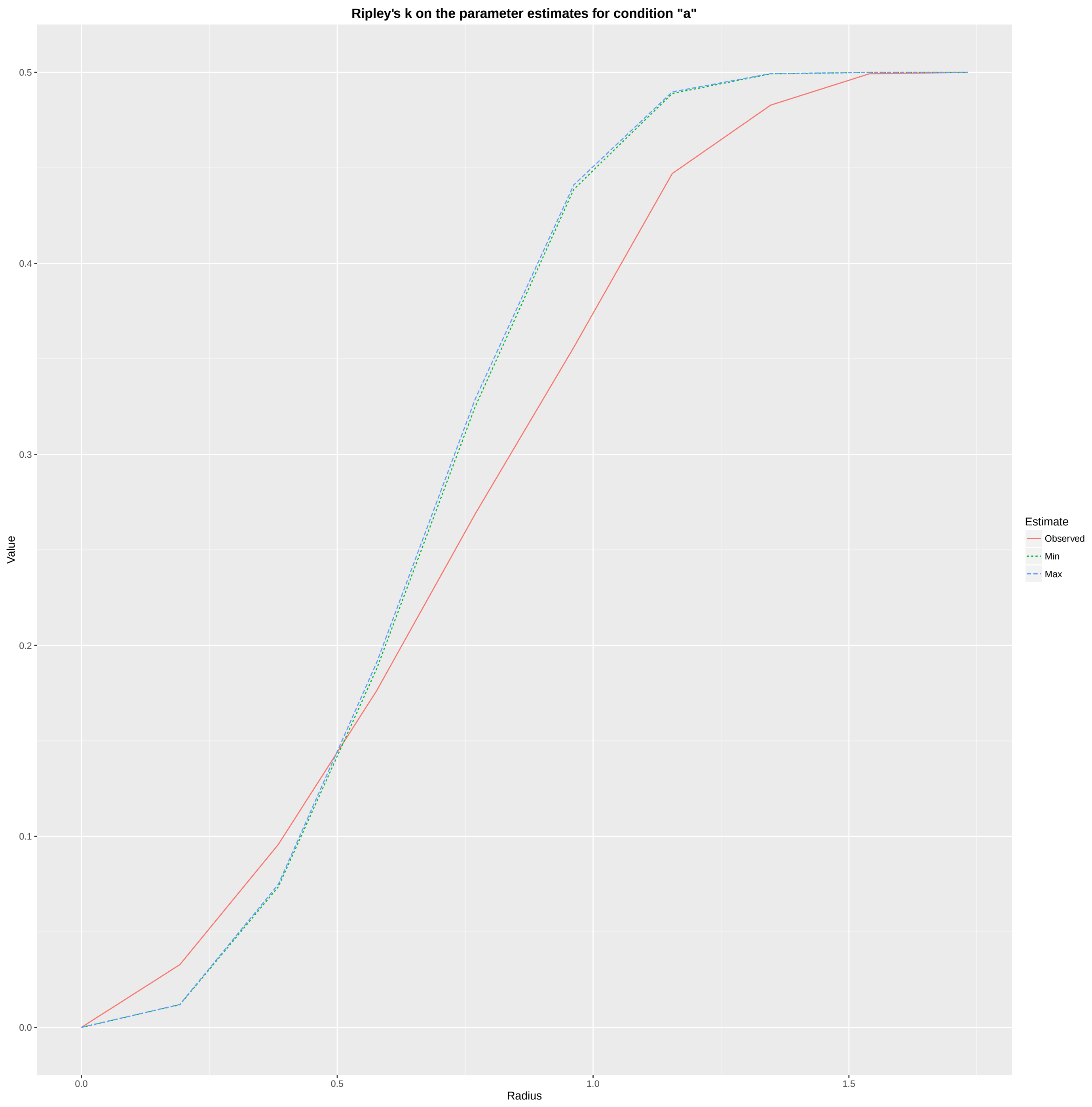

Figure 8: Generalized Ripley's $\hat{k}$ function for the parameter estimates (vertical axis) at 10 distance lags (horizontal axis), showing the actually observed values as well the minimum and maximum values for 100 randomly generated equivalent number of parameter estimates extracted from a Poisson distribution. If the observed $\hat{k}$ is higher than expected then there is significant clustering at that lag, while lower indicate dispersion; otherwise we cannot reject the hypothesis of spatial randomness.

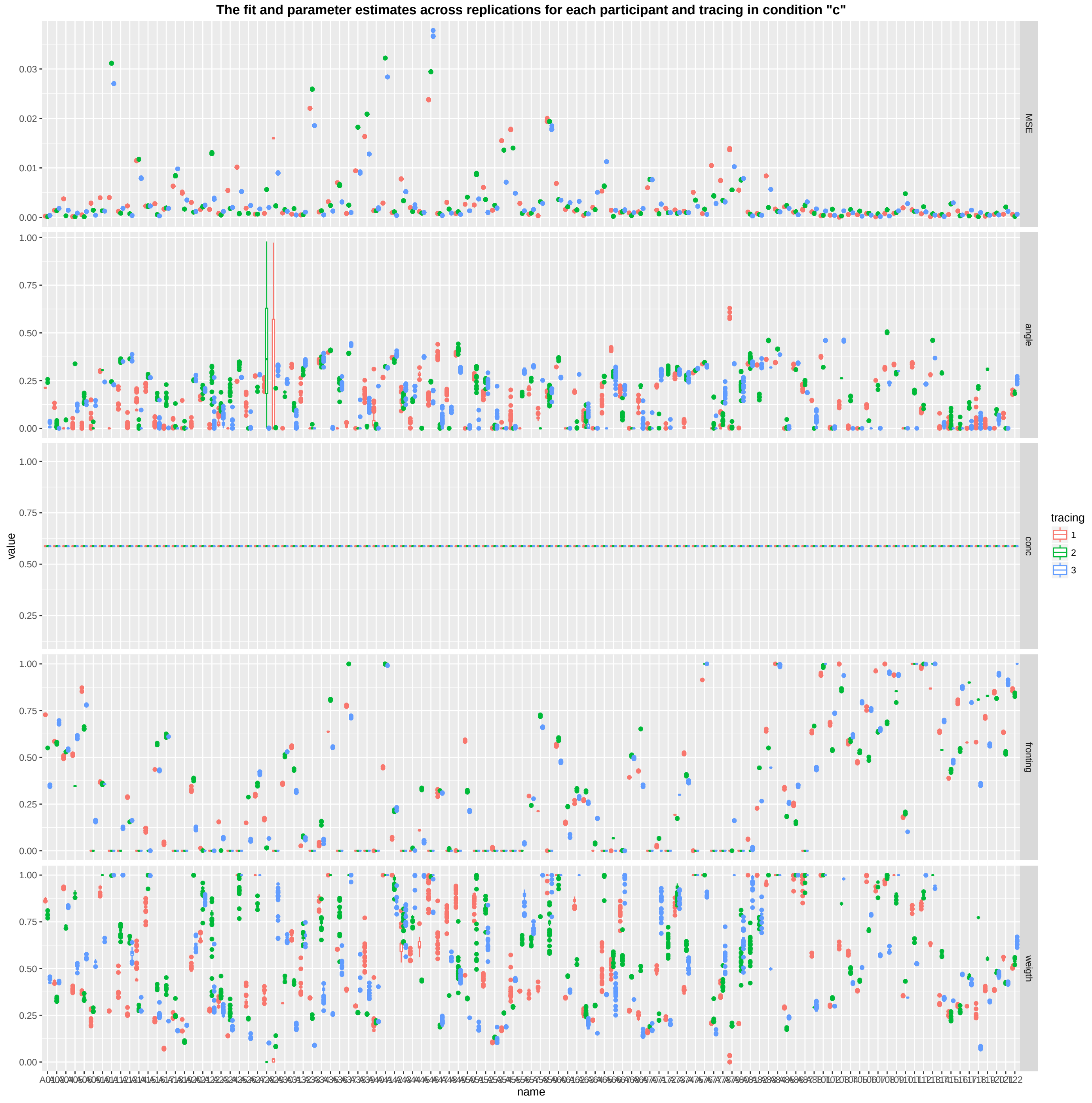

Figure 9: The fit and parameter estimates across the 100 replications for each participant and tracing in condition "c".

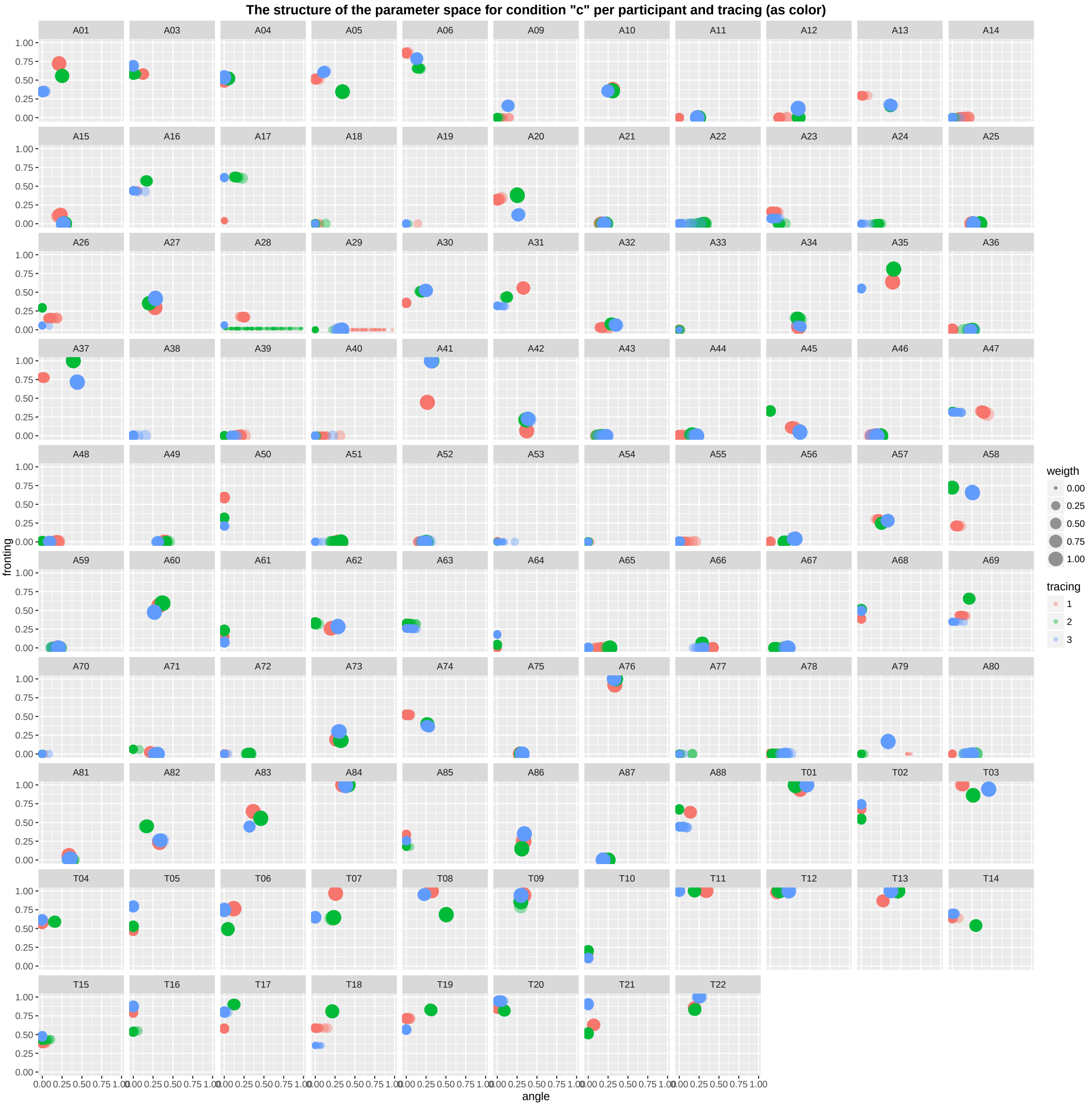

Figure 10: The parameter space across the 100 replications for each participant and tracing in condition “c”.

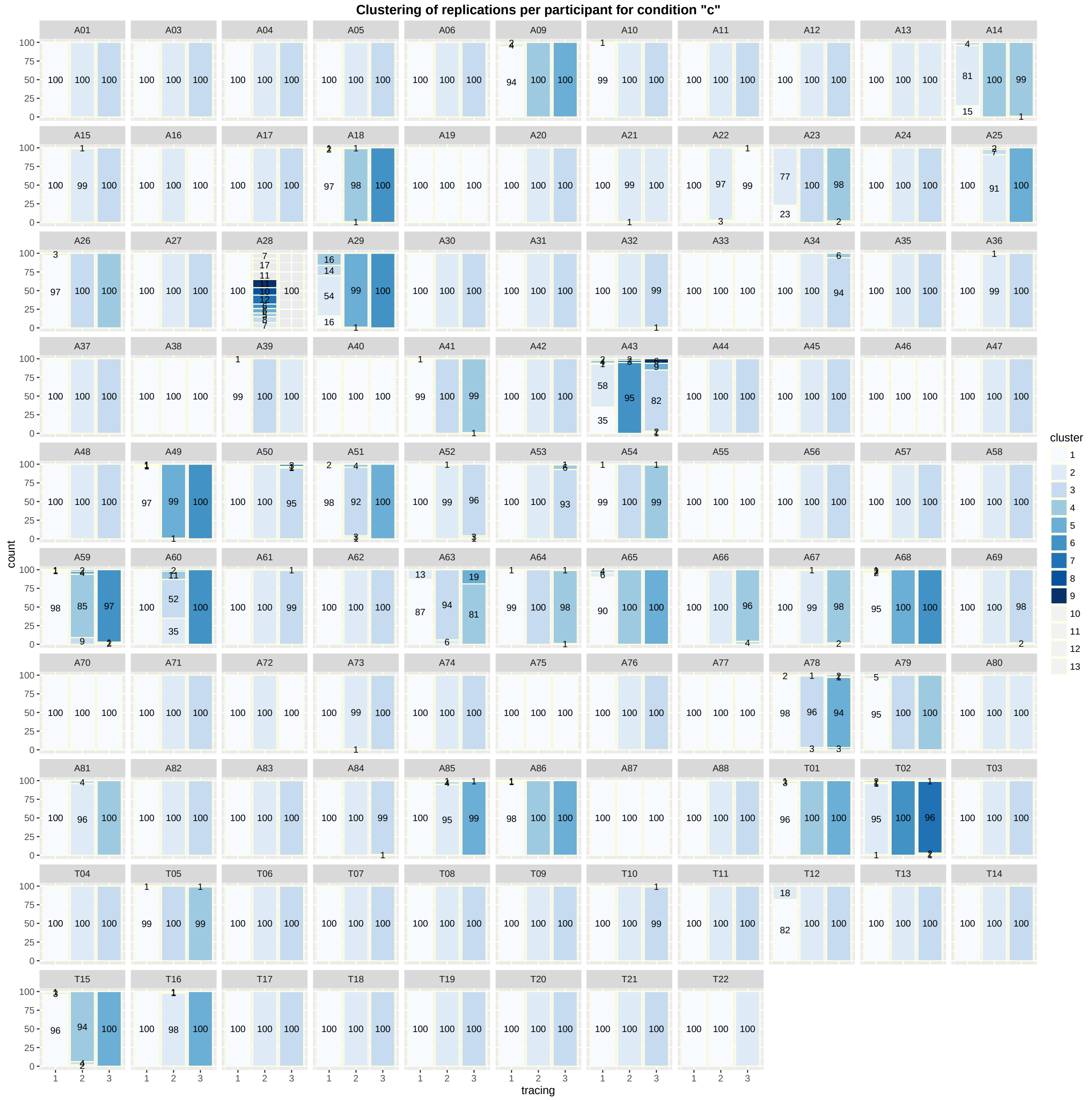

Figure 11: Clustering of replications for each participant and tracing in condition “c” (some participants might be missing because there are no free parameters with non-constant estimates). Numbers give the number of replications in each cluster. Please note that the clusters are specific to each participant (i.e., cluster ”1” for participant ”X” is different than the cluster ”1” for participant ”Y”).

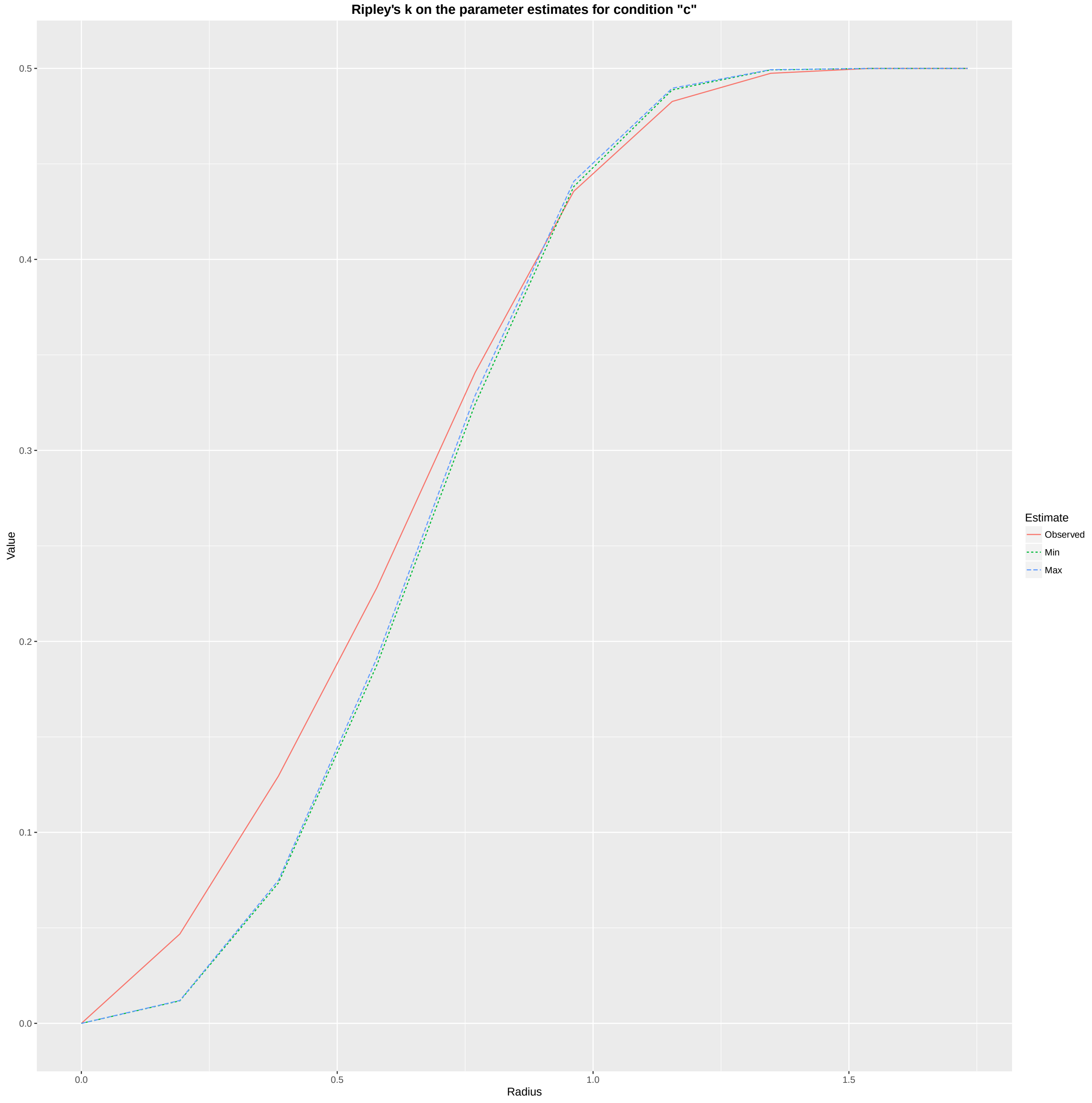

Figure 12: Generalized Ripley's $\hat{k}$ function for the parameter estimates (vertical axis) at 10 distance lags (horizontal axis), showing the actually observed values as well the minimum and maximum values for 100 randomly generated equivalent number of parameter estimates extracted from a Poisson distribution. If the observed $\hat{k}$ is higher than expected then there is significant clustering at that lag, while lower indicate dispersion; otherwise we cannot reject the hypothesis of spatial randomness.

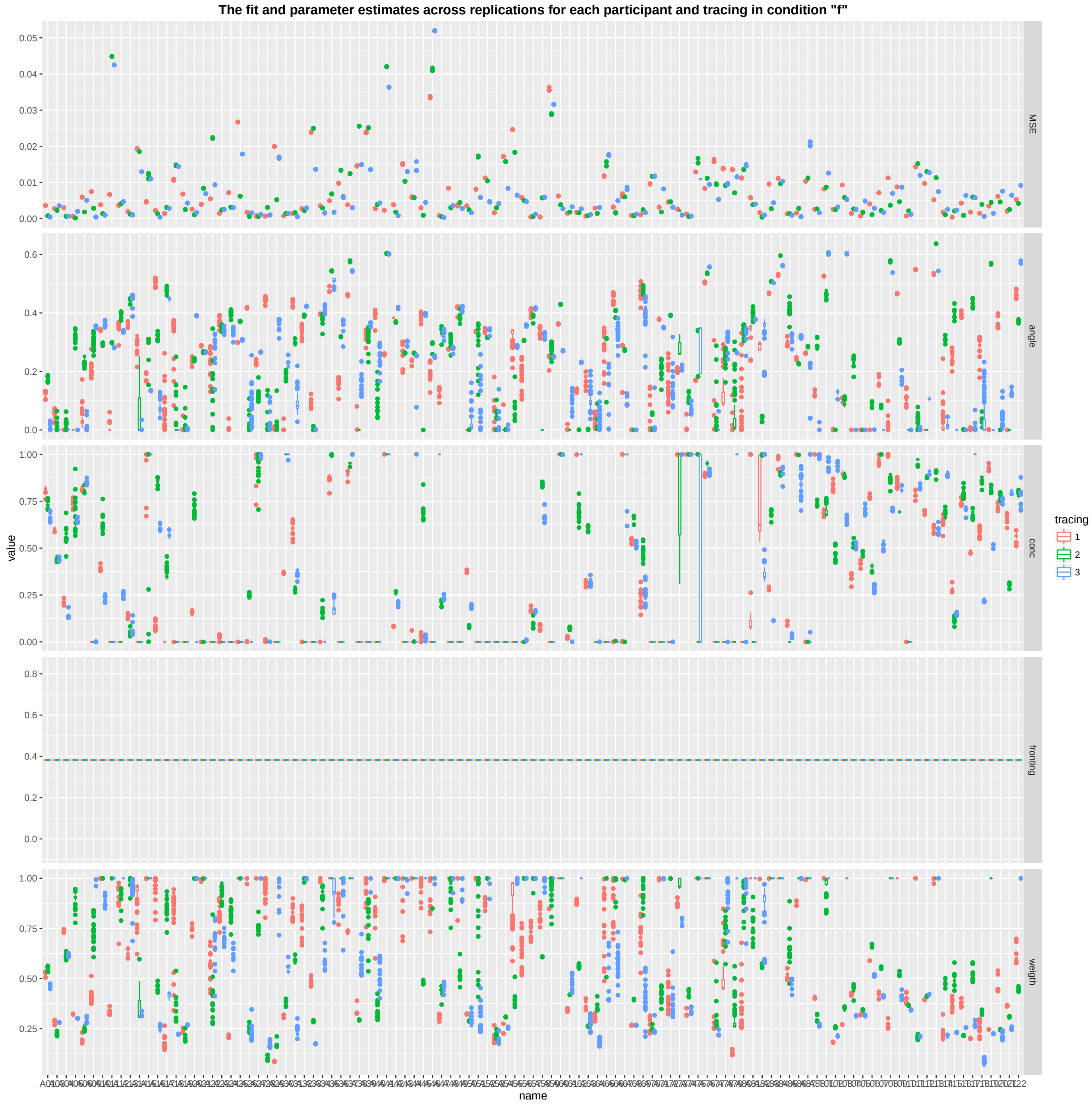

Figure 13: The fit and parameter estimates across the 100 replications for each participant and tracing in condition “f”.

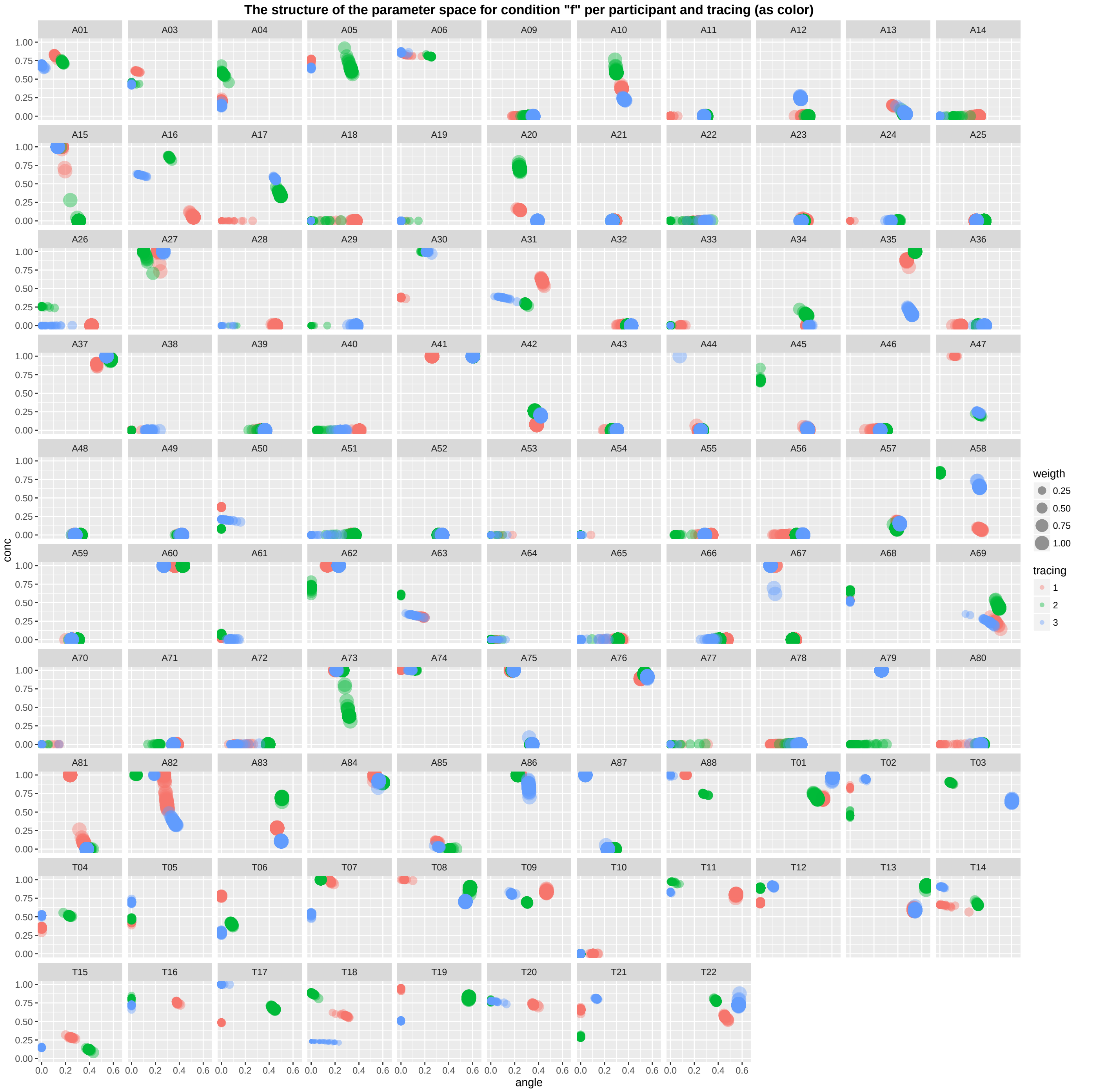

Figure 14: The parameter space across the 100 replications for each participant and tracing in condition “f”.

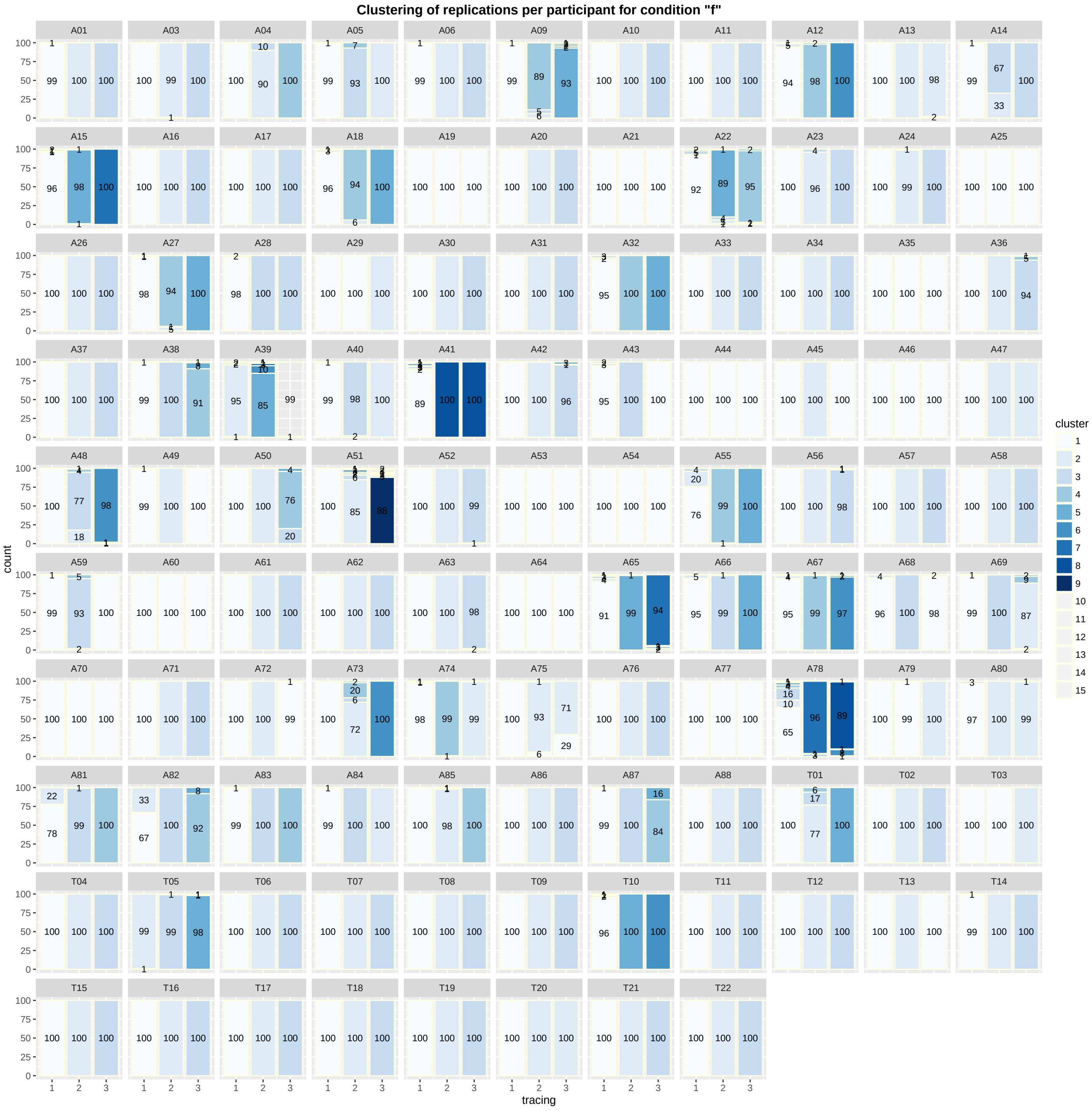

Figure 15: Clustering of replications for each participant and tracing in condition “f” (some participants might be missing because there are no free parameters with non-constant estimates). Numbers give the number of replications in each cluster. Please note that the clusters are specific to each participant (i.e., cluster ”1” for participant ”X” is different than the cluster ”1” for participant ”Y”).

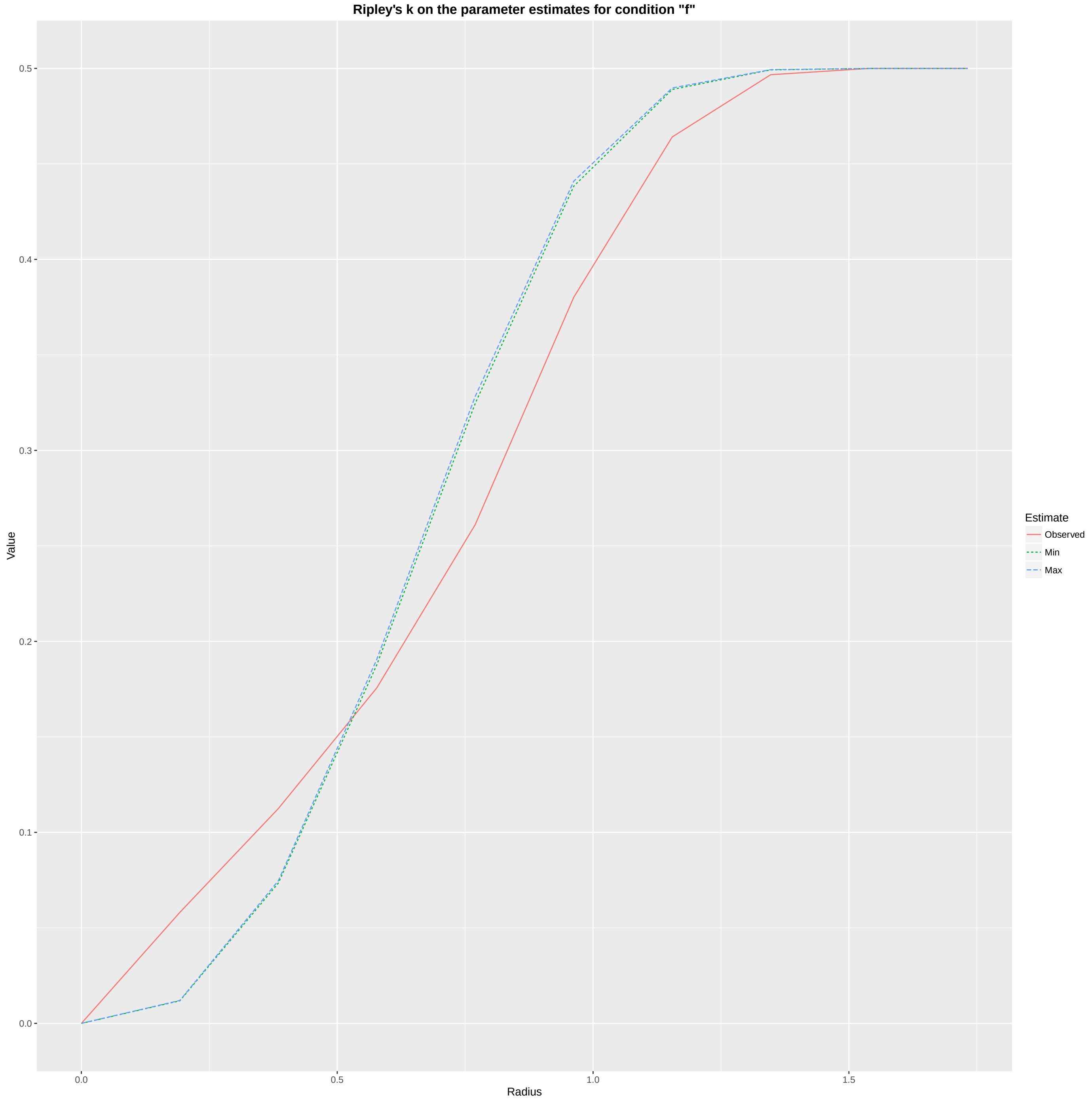

Figure 16: Generalized Ripley's $\hat{k}$ function for the parameter estimates (vertical axis) at 10 distance lags (horizontal axis), showing the actually observed values as well the minimum and maximum values for 100 randomly generated equivalent number of parameter estimates extracted from a Poisson distribution. If the observed $\hat{k}$ is higher than expected then there is significant clustering at that lag, while lower indicate dispersion; otherwise we cannot reject the hypothesis of spatial randomness.

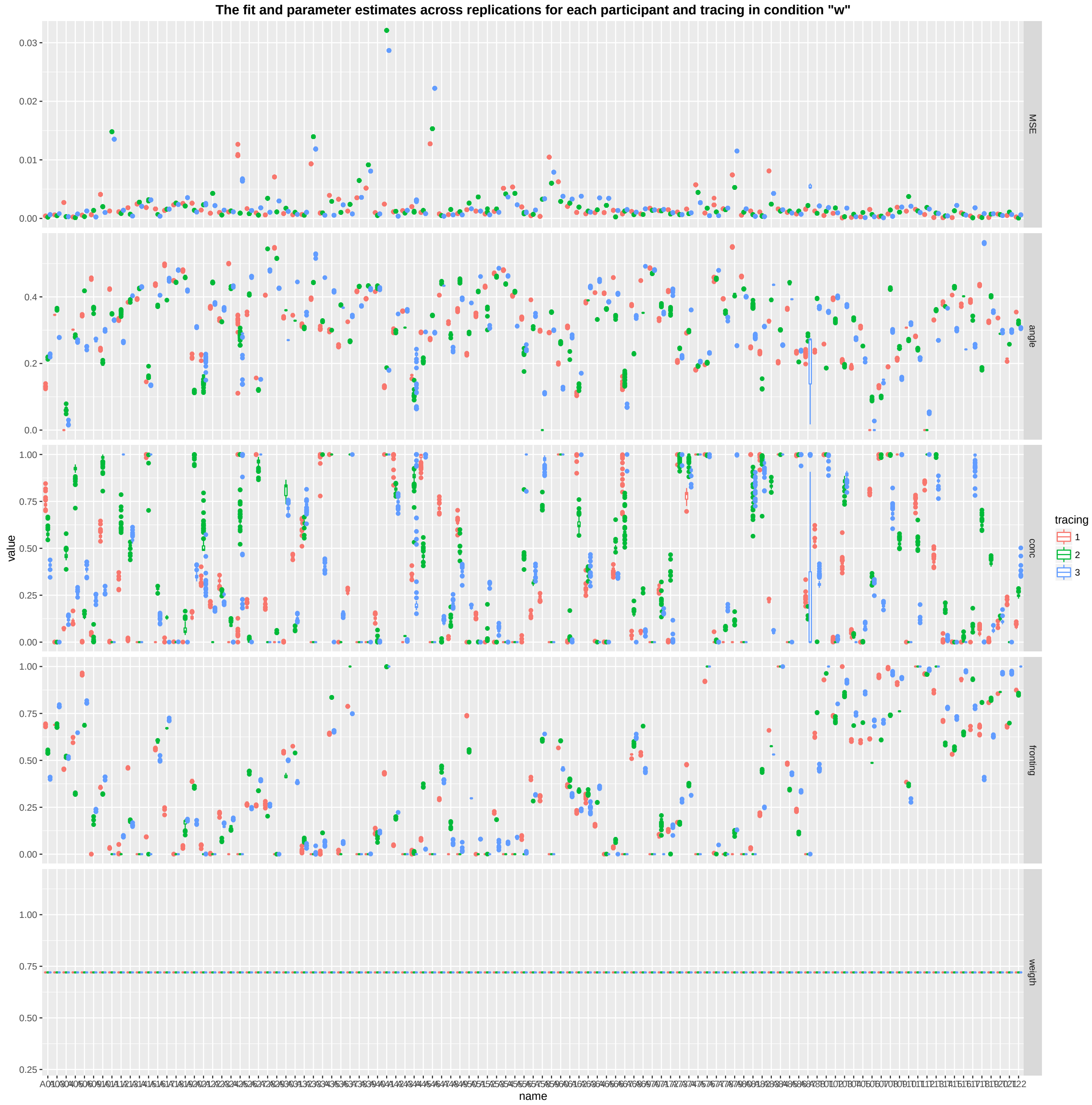

Figure 17: The fit and parameter estimates across the 100 replications for each participant and tracing in condition “w”.

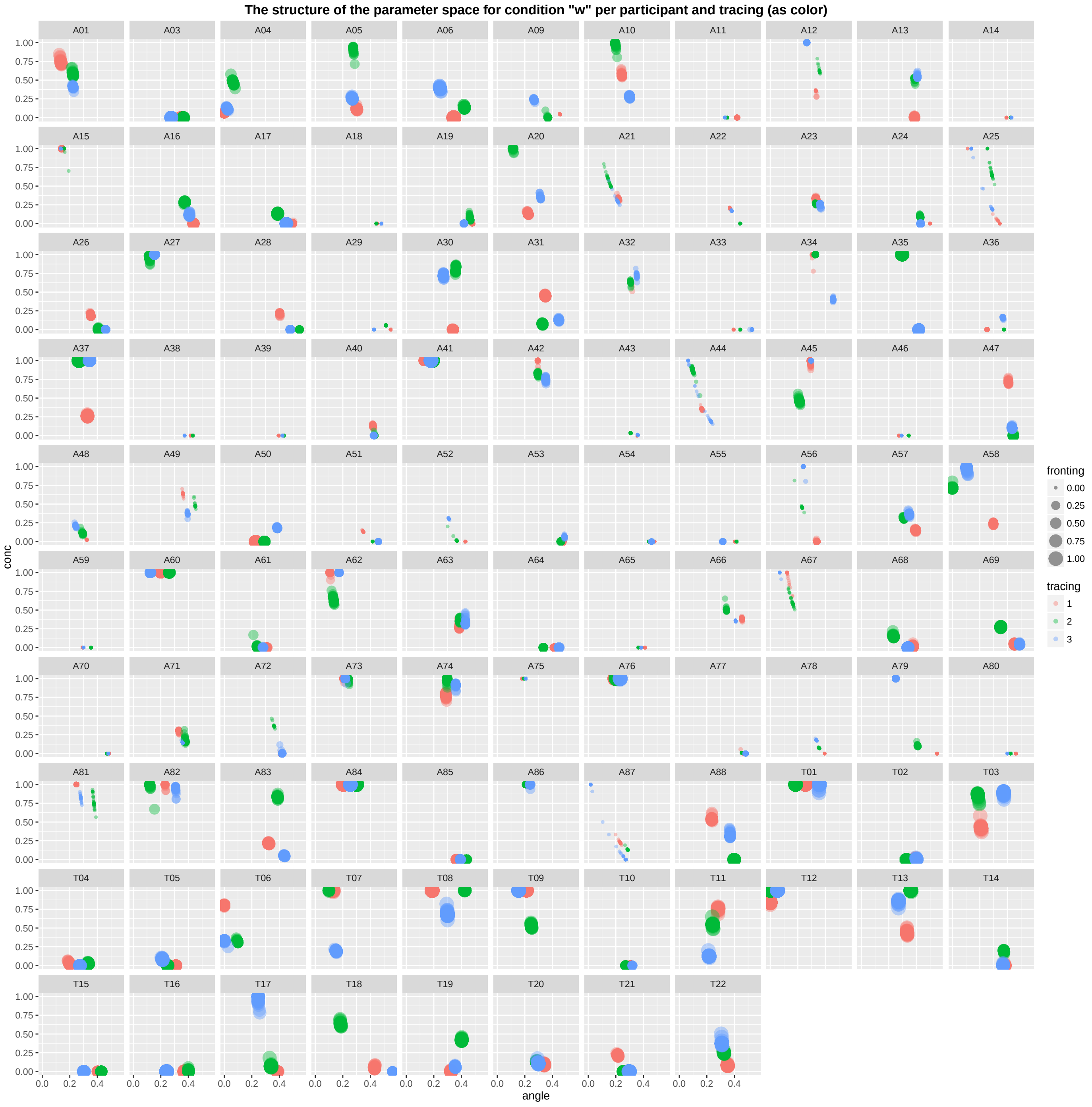

Figure 18: The parameter space across the 100 replications for each participant and tracing in condition “w”.

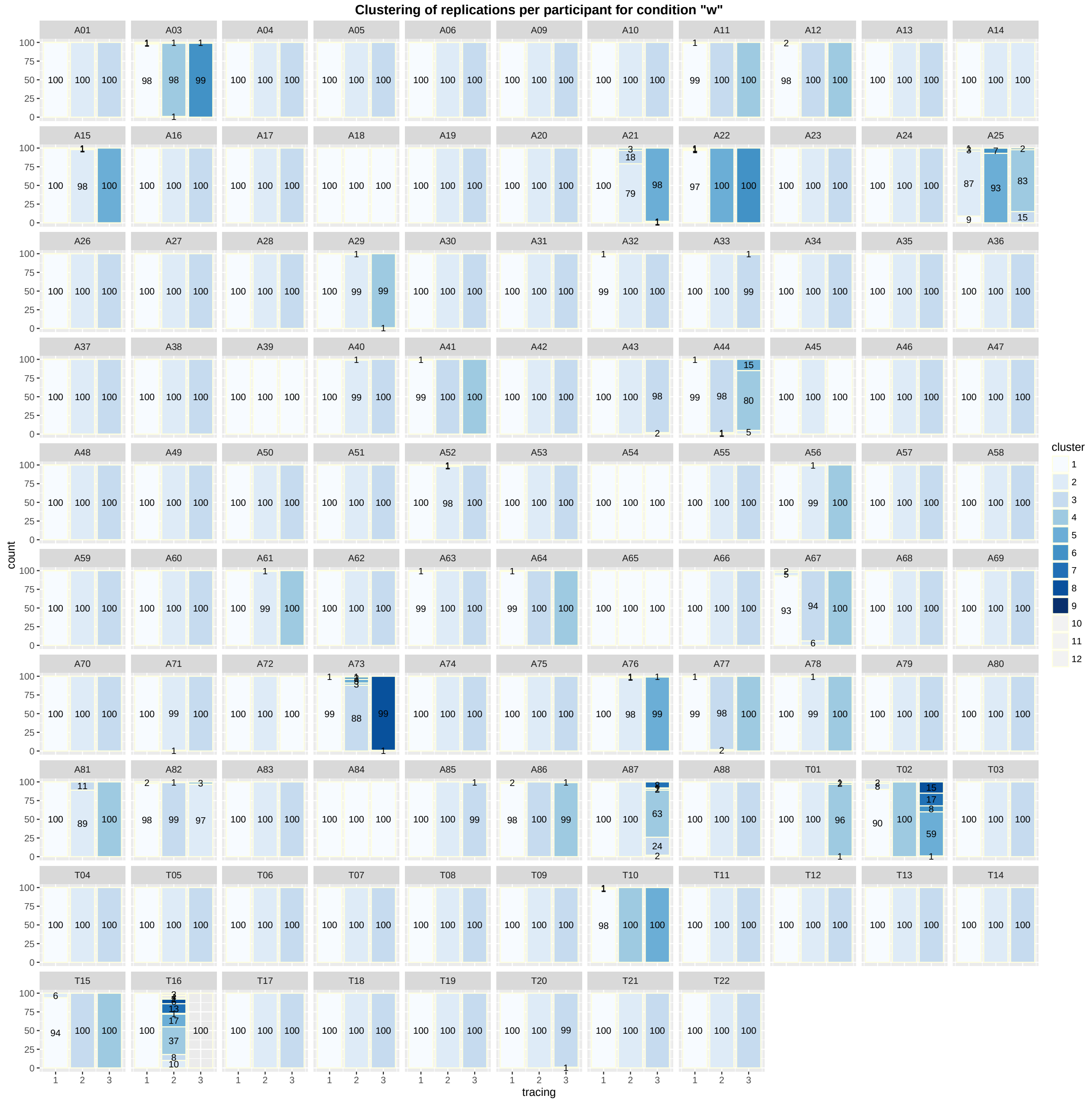

Figure 19: Clustering of replications for each participant and tracing in condition “w” (some participants might be missing because there are no free parameters with non-constant estimates). Numbers give the number of replications in each cluster. Please note that the clusters are specific to each participant (i.e., cluster ”1” for participant ”X” is different than the cluster ”1” for participant ”Y”).

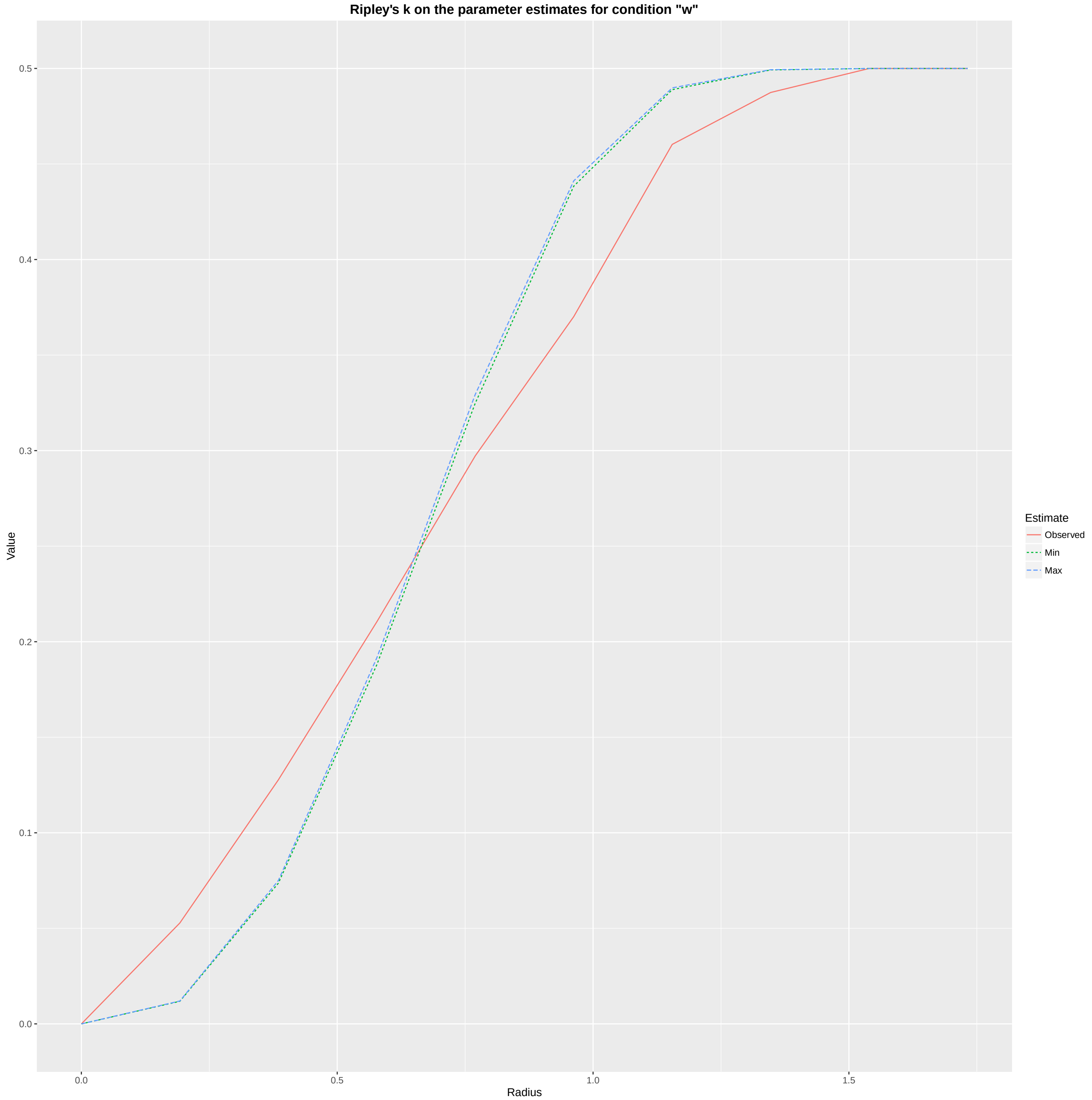

Figure 20: Generalized Ripley's $\hat{k}$ function for the parameter estimates (vertical axis) at 10 distance lags (horizontal axis), showing the actually observed values as well the minimum and maximum values for 100 randomly generated equivalent number of parameter estimates extracted from a Poisson distribution. If the observed $\hat{k}$ is higher than expected then there is significant clustering at that lag, while lower indicate dispersion; otherwise we cannot reject the hypothesis of spatial randomness.

3 Conditions with two fixed parameters (“ac”, “af”, “aw”, “cf”, “cw” and “fw”)

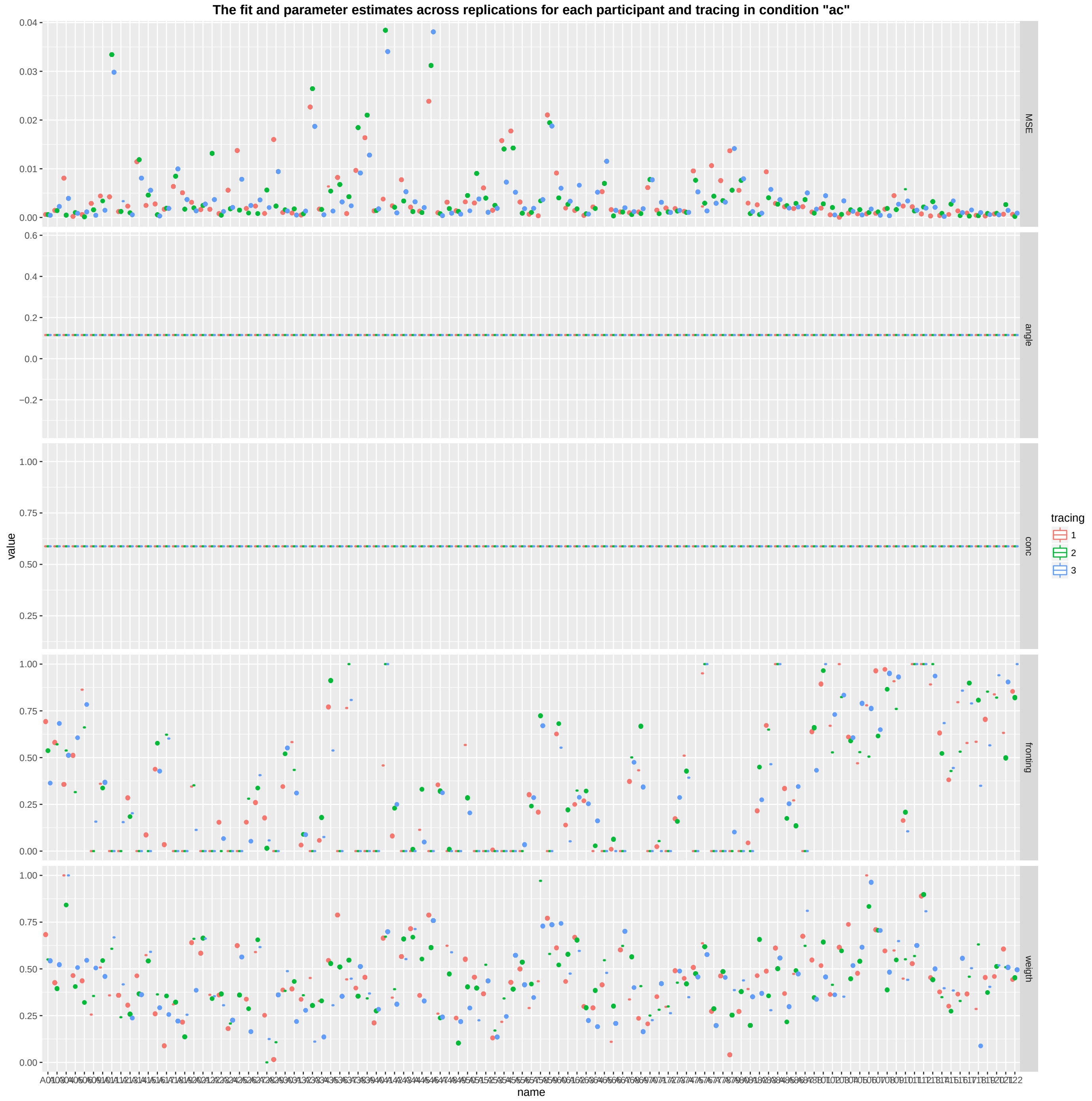

Figure 21: The fit and parameter estimates across the 100 replications for each participant and tracing in condition “ac”.

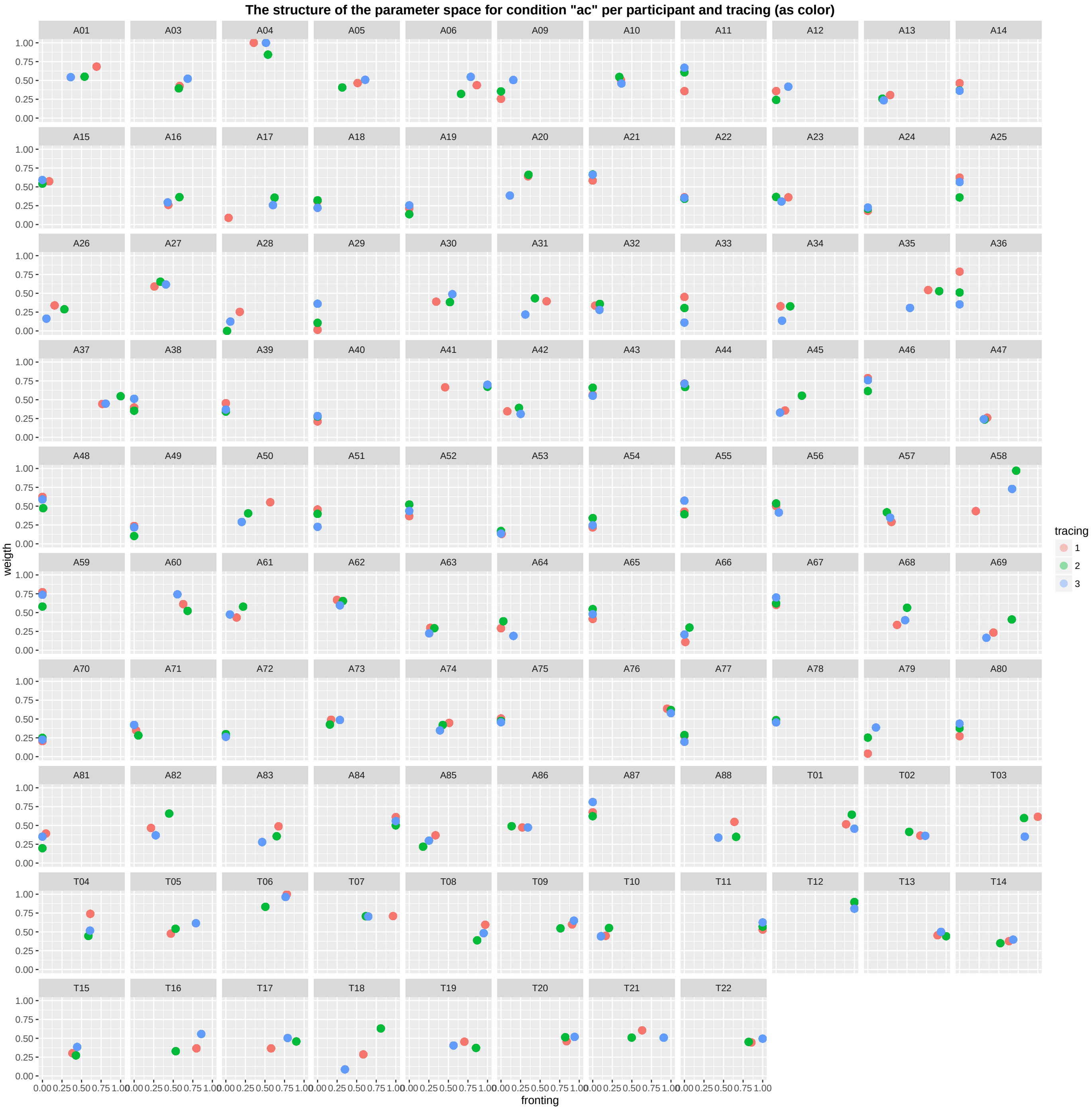

Figure 22: The parameter space across the 100 replications for each participant and tracing in condition "ac".

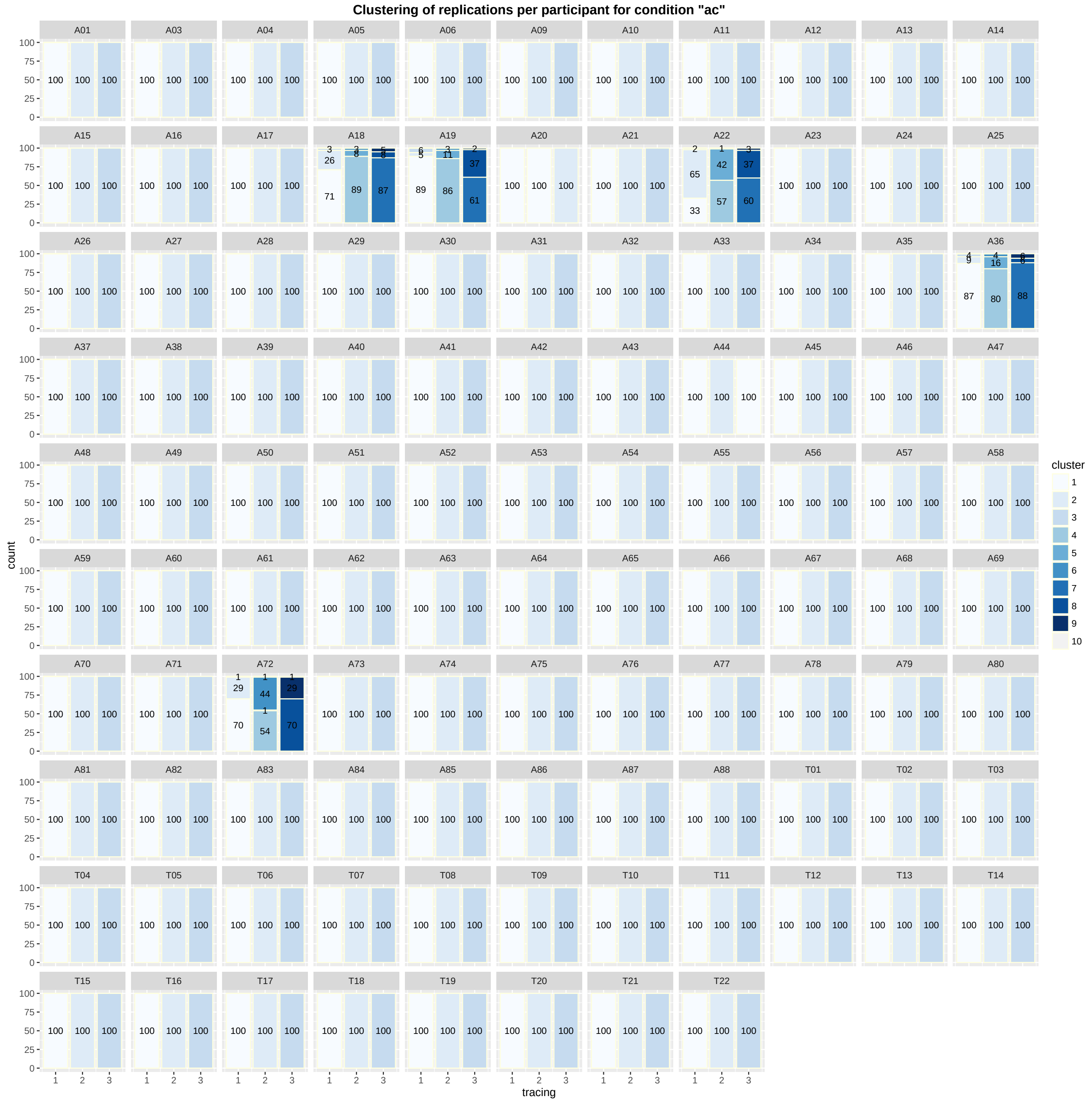

Figure 23: Clustering of replications for each participant and tracing in condition “ac” (some participants might be missing because there are no free parameters with non-constant estimates). Numbers give the number of replications in each cluster. Please note that the clusters are specific to each participant (i.e., cluster ”1” for participant ”X” is different than the cluster ”1” for participant ”Y”).

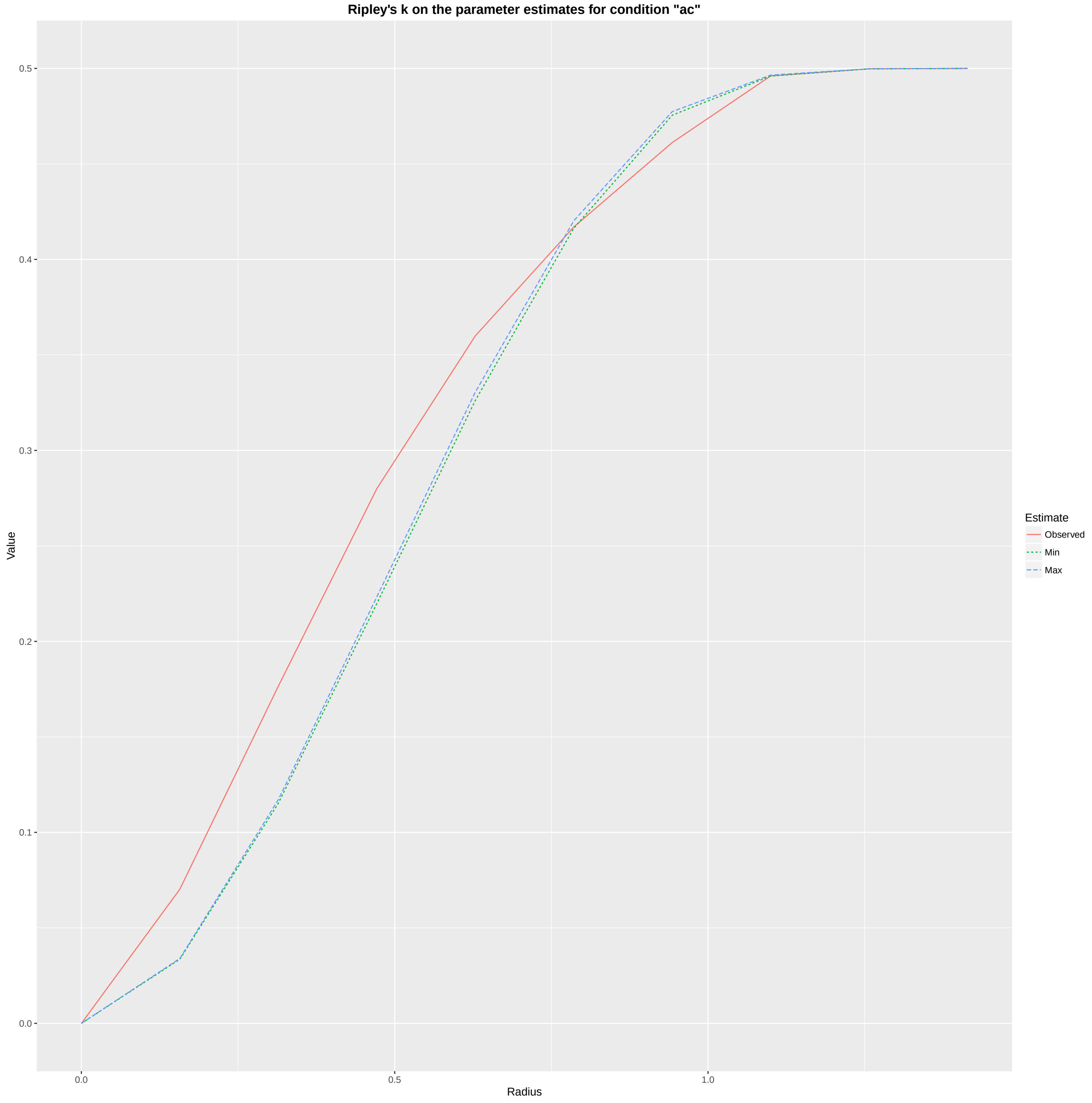

Figure 24: Generalized Ripley's $\hat{k}$ function for the parameter estimates (vertical axis) at 10 distance lags (horizontal axis), showing the actually observed values as well the minimum and maximum values for 100 randomly generated equivalent number of parameter estimates extracted from a Poisson distribution. If the observed $\hat{k}$ is higher than expected then there is significant clustering at that lag, while lower indicate dispersion; otherwise we cannot reject the hypothesis of spatial randomness.

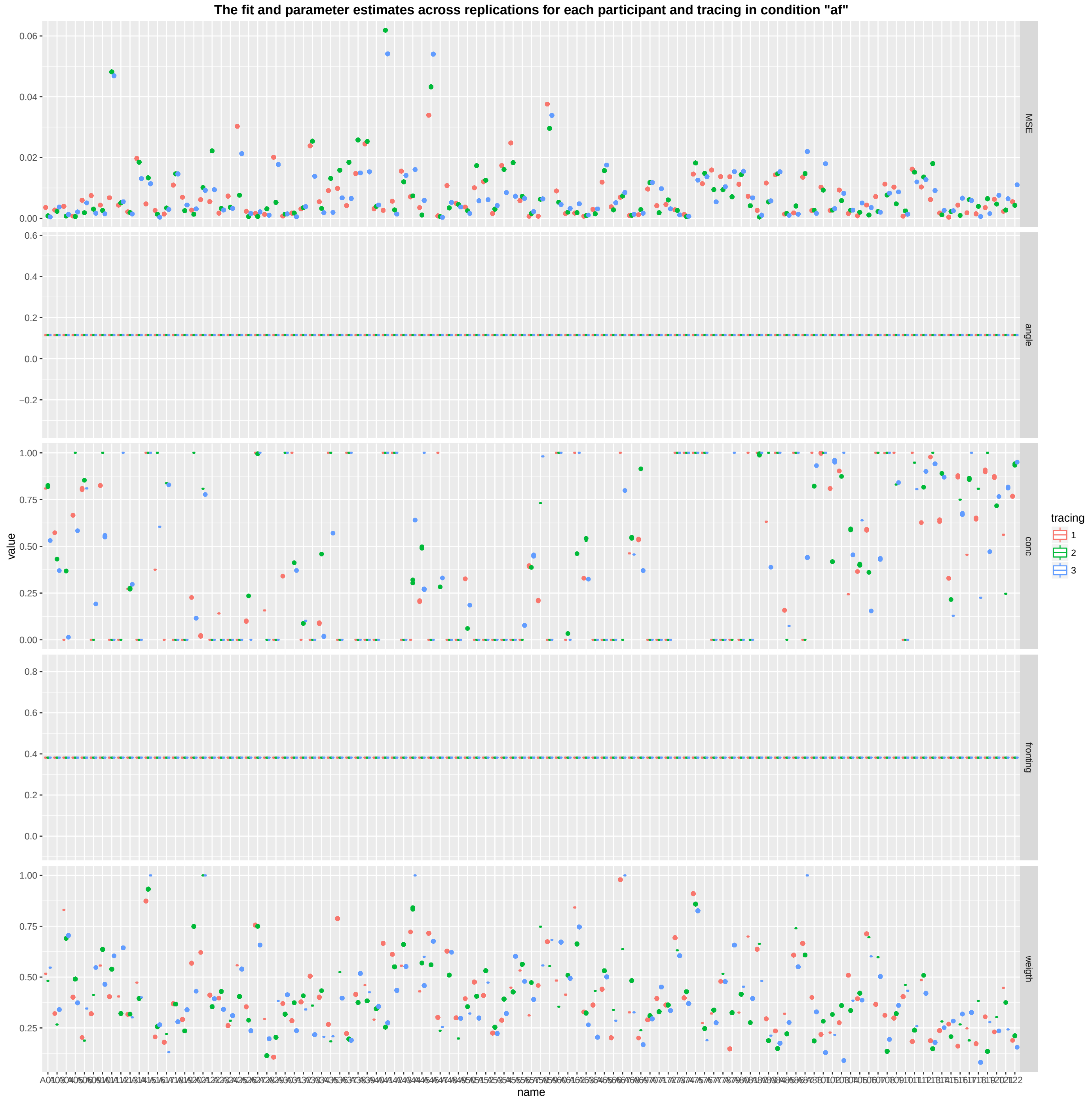

Figure 25: The fit and parameter estimates across the 100 replications for each participant and tracing in condition “af”.

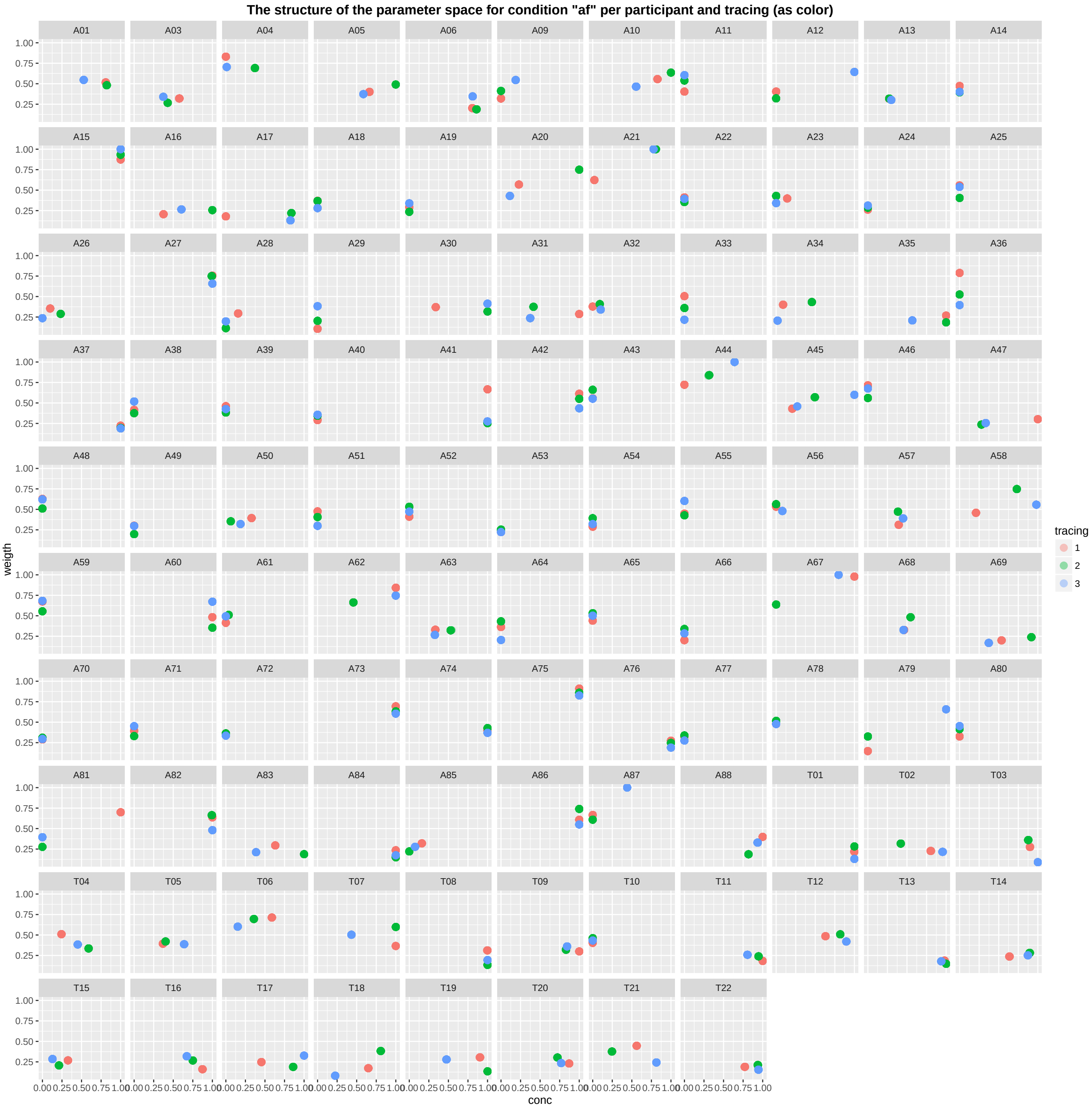

Figure 26: The parameter space across the 100 replications for each participant and tracing in condition “af”.

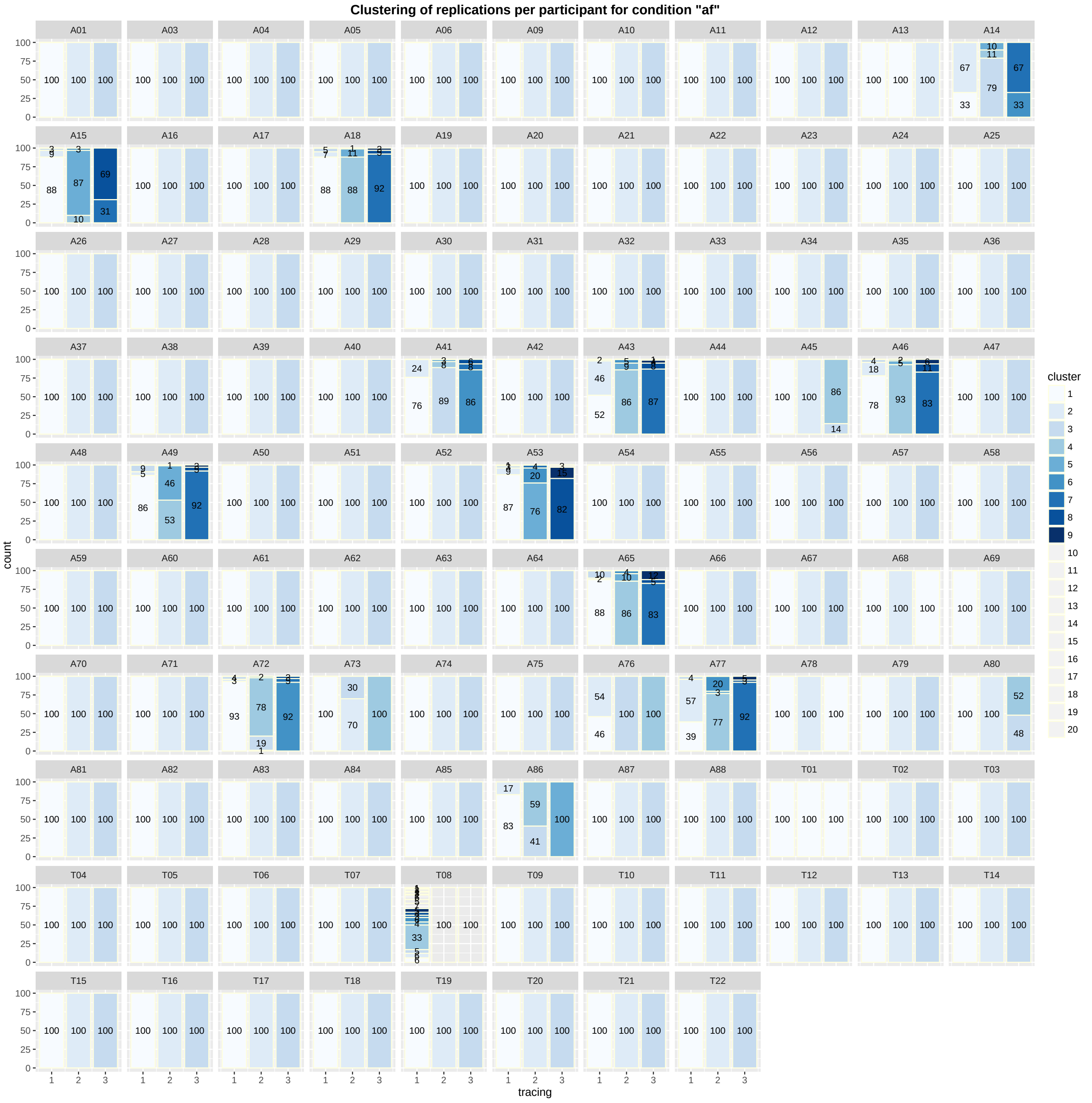

Figure 27: Clustering of replications for each participant and tracing in condition “af” (some participants might be missing because there are no free parameters with non-constant estimates). Numbers give the number of replications in each cluster. Please note that the clusters are specific to each participant (i.e., cluster ”1” for participant ”X” is different than the cluster ”1” for participant ”Y”).

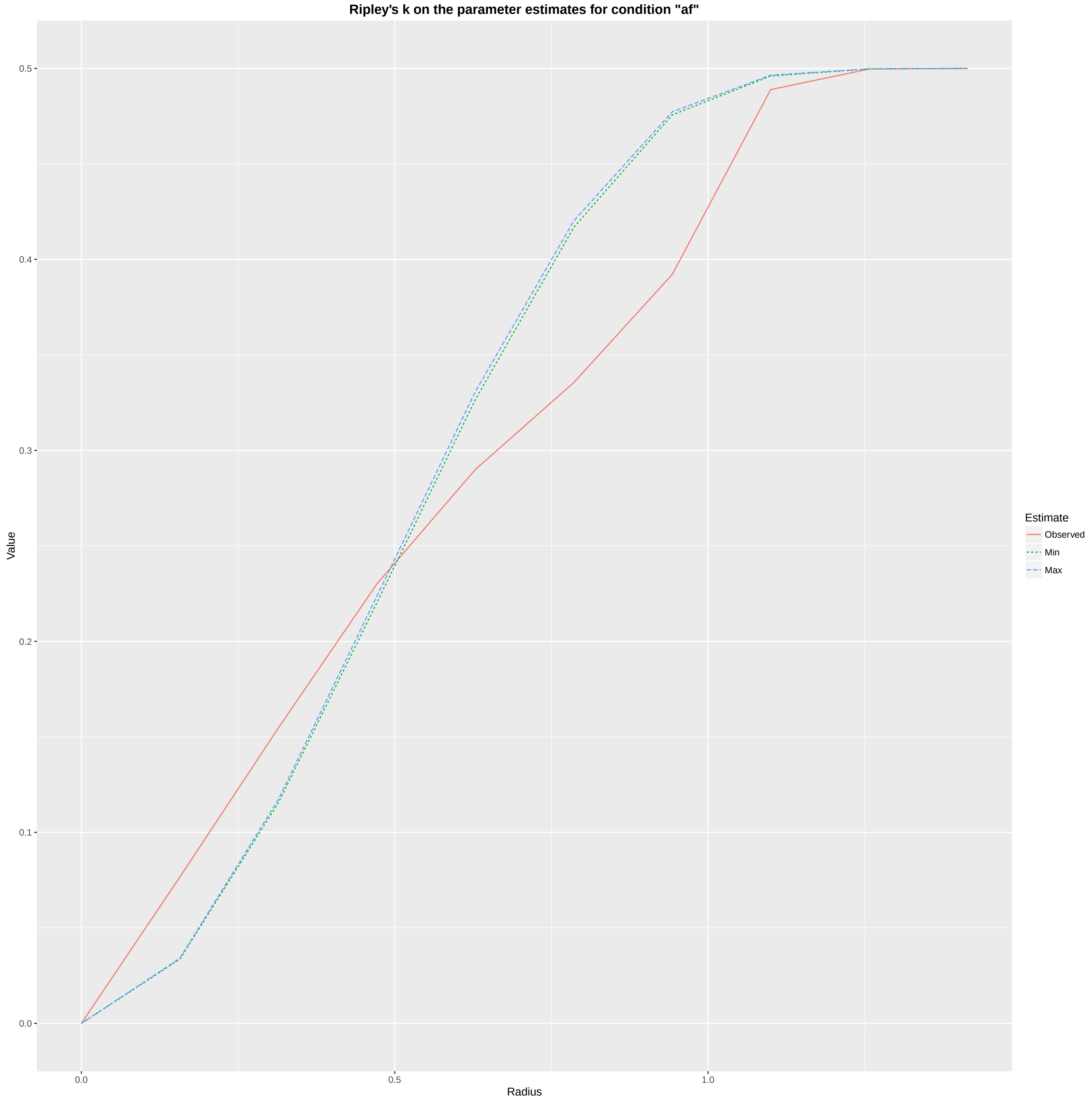

Figure 28: Generalized Ripley's $\hat{k}$ function for the parameter estimates (vertical axis) at 10 distance lags (horizontal axis), showing the actually observed values as well the minimum and maximum values for 100 randomly generated equivalent number of parameter estimates extracted from a Poisson distribution. If the observed $\hat{k}$ is higher than expected then there is significant clustering at that lag, while lower indicate dispersion; otherwise we cannot reject the hypothesis of spatial randomness.

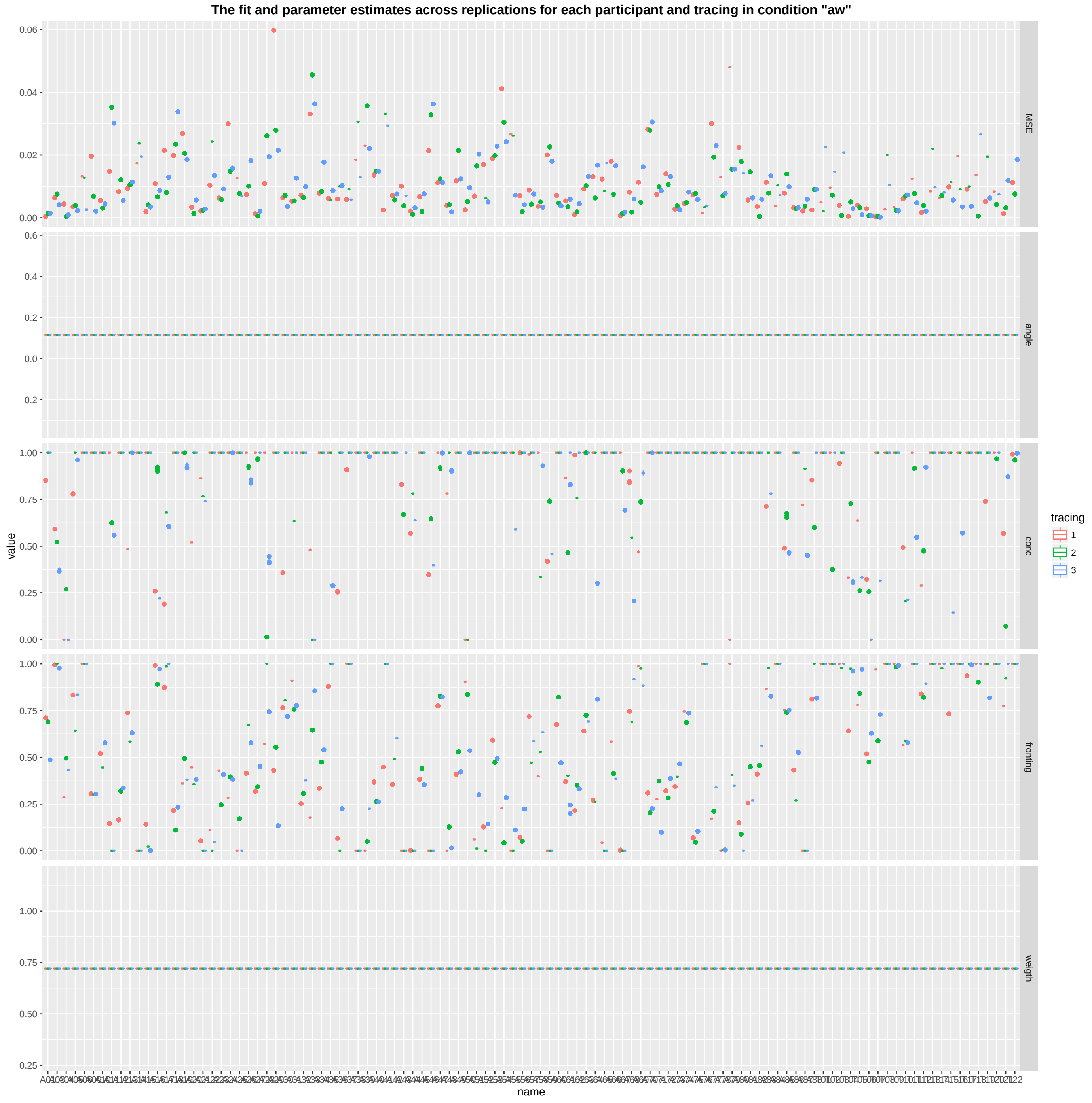

Figure 29: The fit and parameter estimates across the 100 replications for each participant and tracing in condition “aw”.

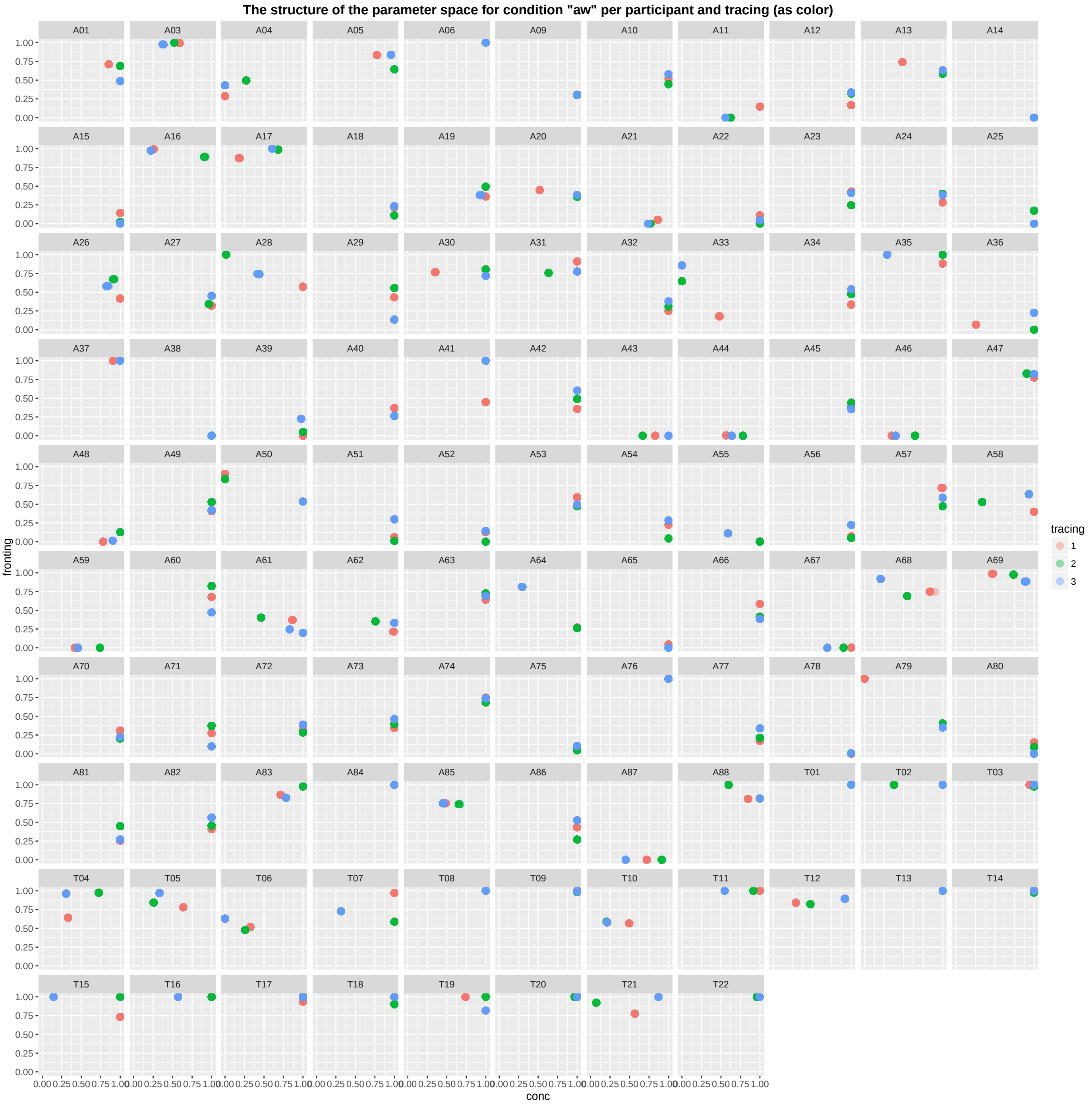

Figure 30: The parameter space across the 100 replications for each participant and tracing in condition "aw".

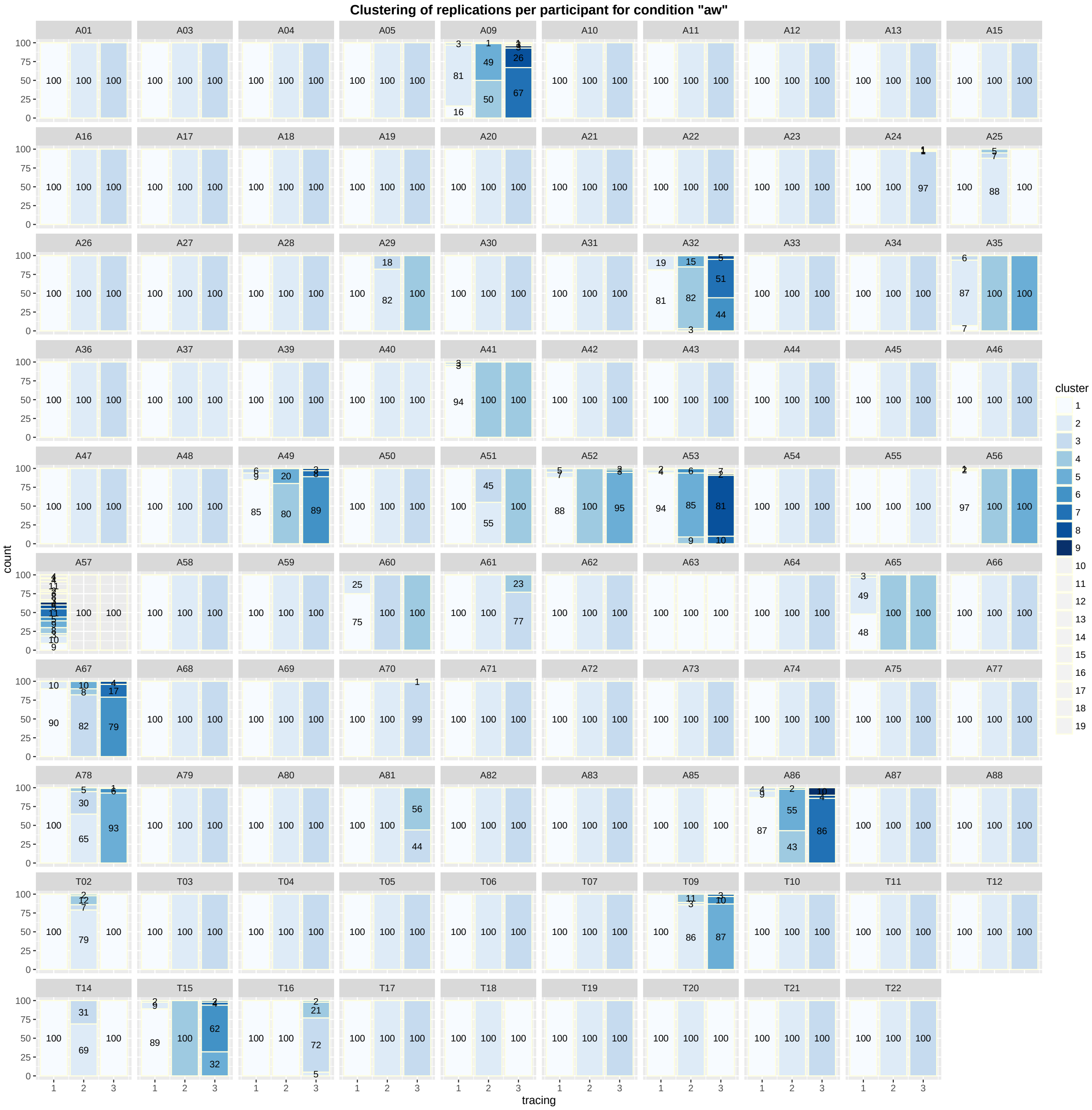

Figure 31: Clustering of replications for each participant and tracing in condition “aw” (some participants might be missing because there are no free parameters with non-constant estimates). Numbers give the number of replications in each cluster. Please note that the clusters are specific to each participant (i.e., cluster ”1” for participant ”X” is different than the cluster ”1” for participant ”Y”).

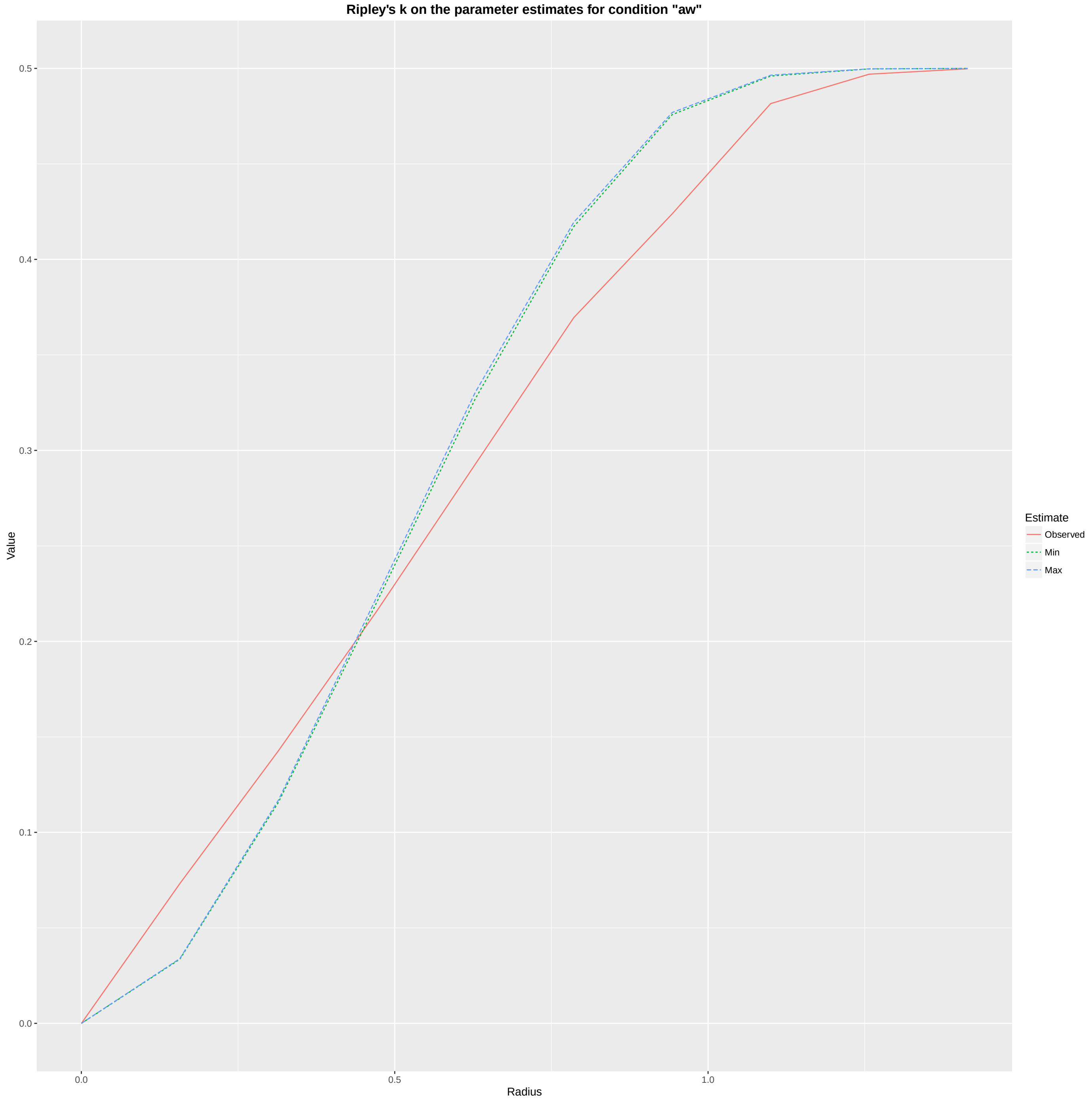

Figure 32: Generalized Ripley's $\hat{k}$ function for the parameter estimates (vertical axis) at 10 distance lags (horizontal axis), showing the actually observed values as well the minimum and maximum values for 100 randomly generated equivalent number of parameter estimates extracted from a Poisson distribution. If the observed $\hat{k}$ is higher than expected then there is significant clustering at that lag, while lower indicate dispersion; otherwise we cannot reject the hypothesis of spatial randomness.

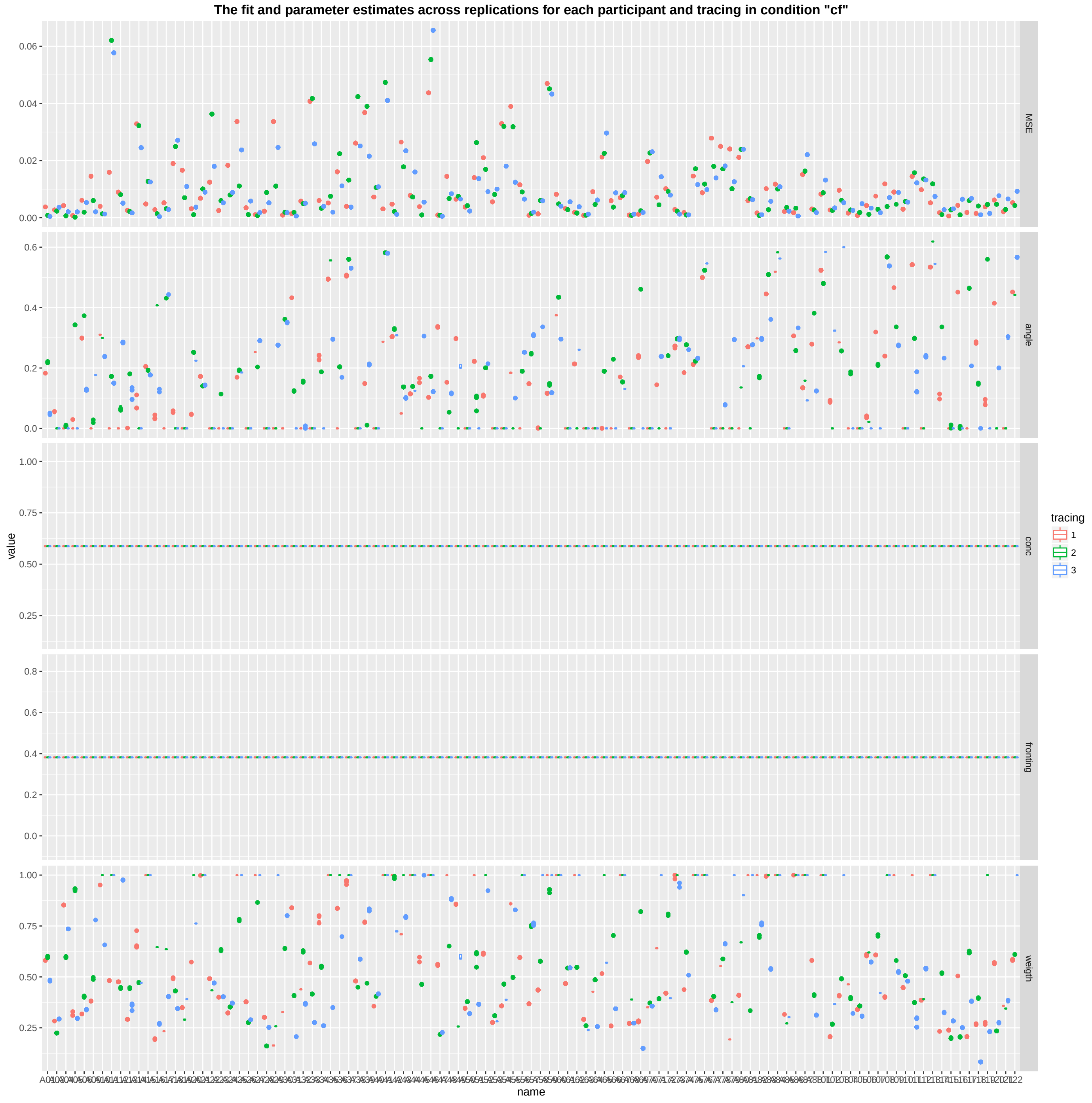

Figure 33: The fit and parameter estimates across the 100 replications for each participant and tracing in condition “cf”.

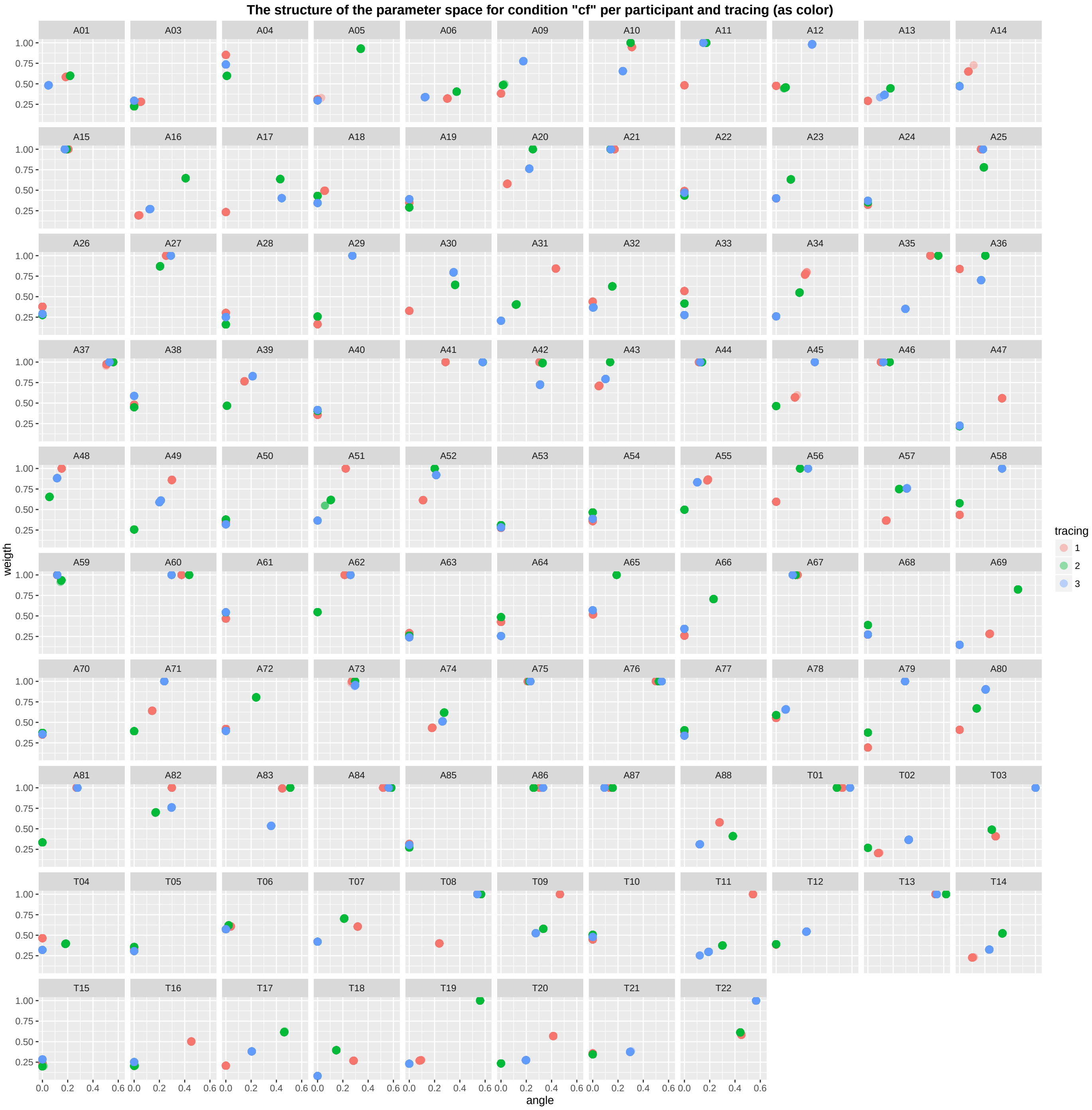

Figure 34: The parameter space across the 100 replications for each participant and tracing in condition “cf”.

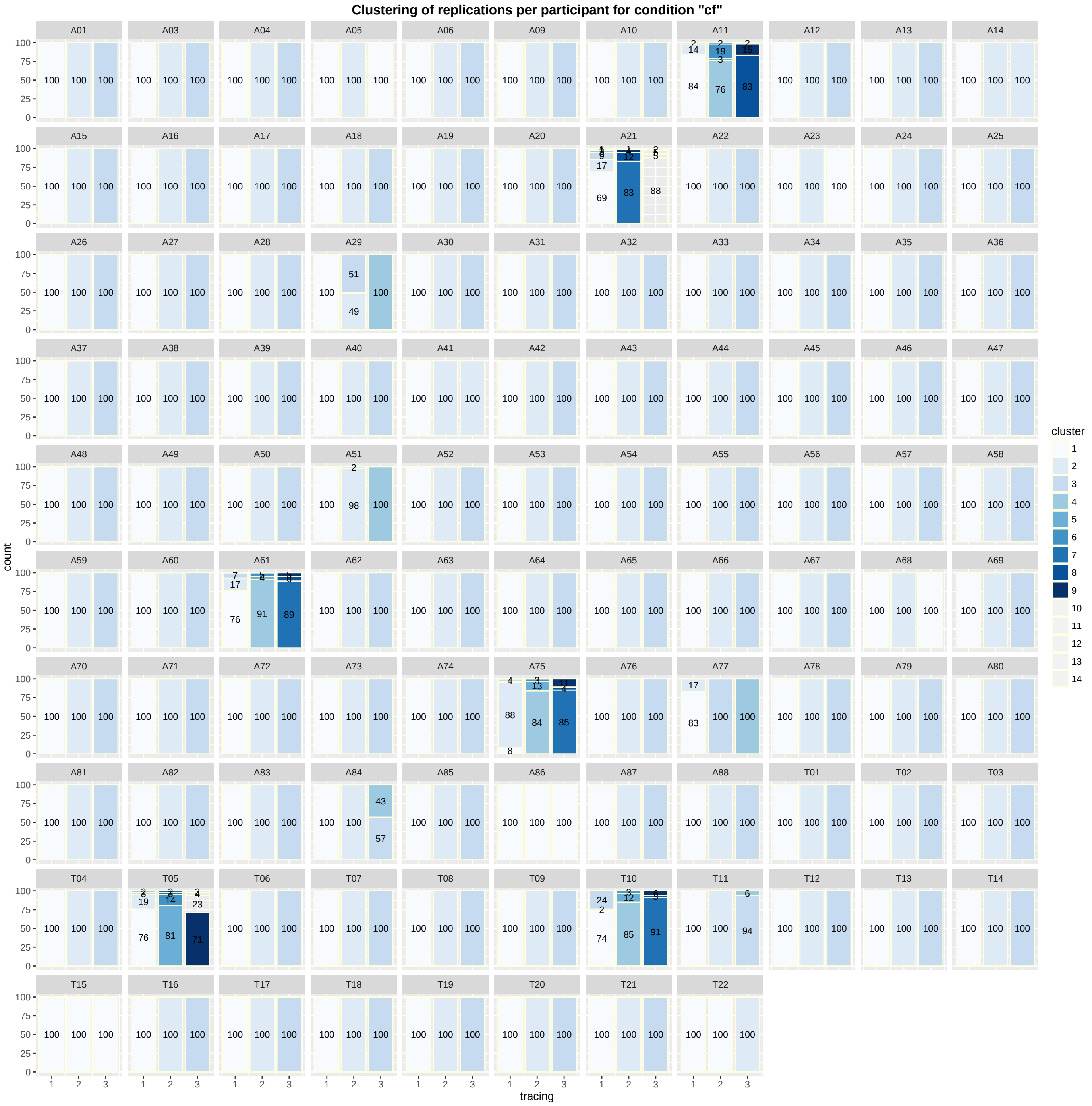

Figure 35: Clustering of replications for each participant and tracing in condition “cf” (some participants might be missing because there are no free parameters with non-constant estimates). Numbers give the number of replications in each cluster. Please note that the clusters are specific to each participant (i.e., cluster ”1” for participant ”X” is different than the cluster ”1” for participant ”Y”).

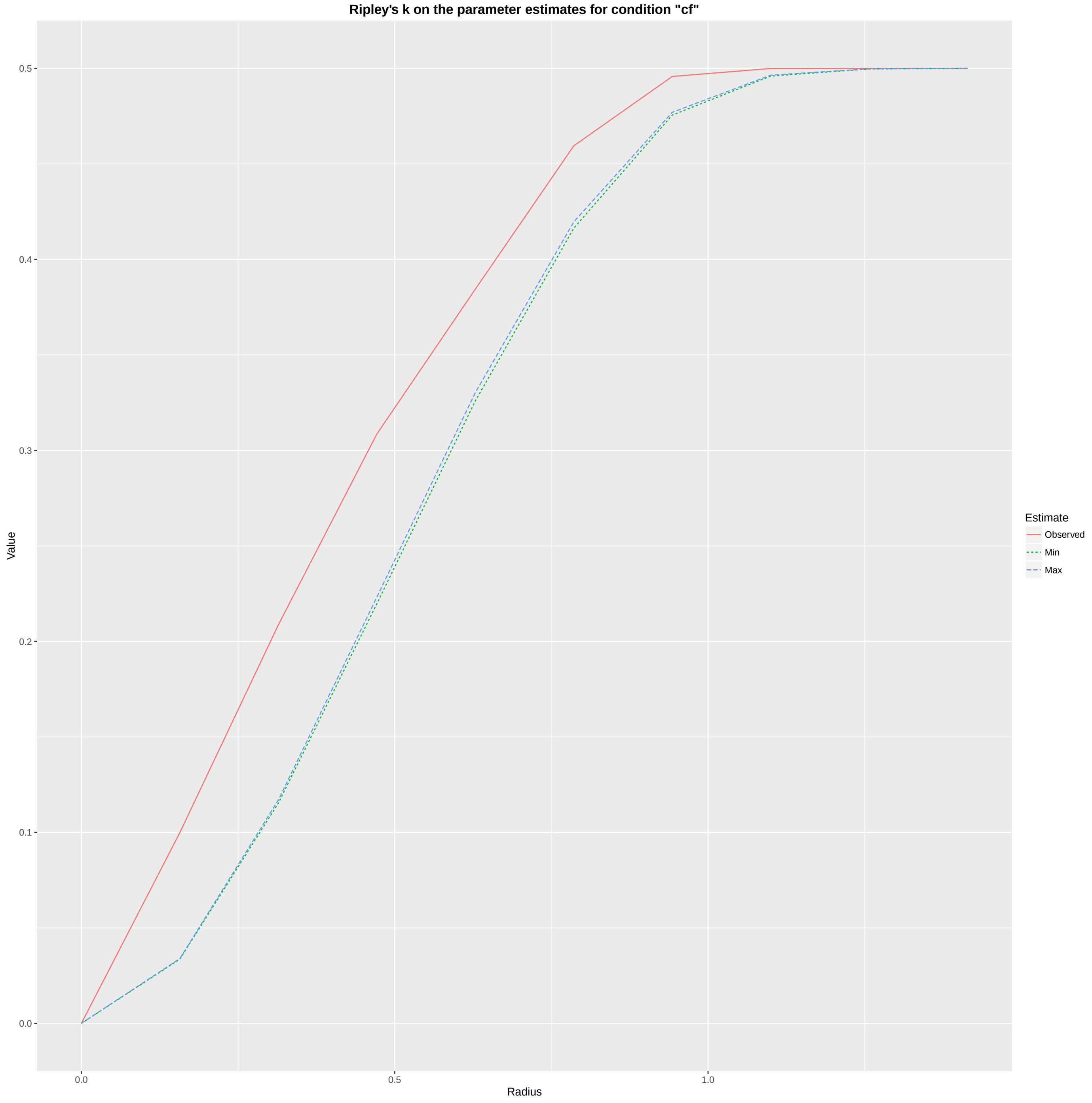

Figure 36: Generalized Ripley's $\hat{k}$ function for the parameter estimates (vertical axis) at 10 distance lags (horizontal axis), showing the actually observed values as well the minimum and maximum values for 100 randomly generated equivalent number of parameter estimates extracted from a Poisson distribution. If the observed $\hat{k}$ is higher than expected then there is significant clustering at that lag, while lower indicate dispersion; otherwise we cannot reject the hypothesis of spatial randomness.

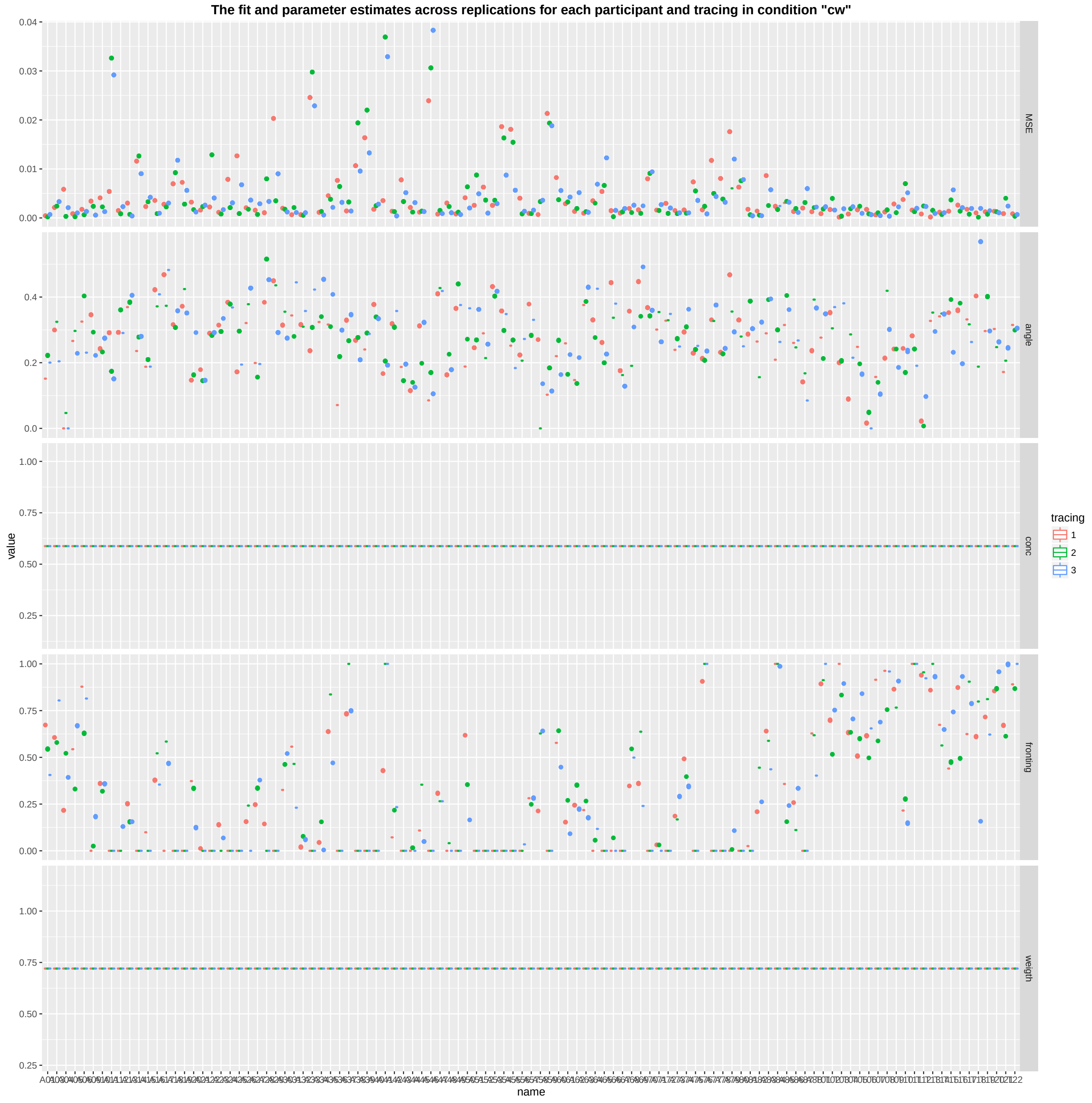

Figure 37: The fit and parameter estimates across the 100 replications for each participant and tracing in condition “cw”.

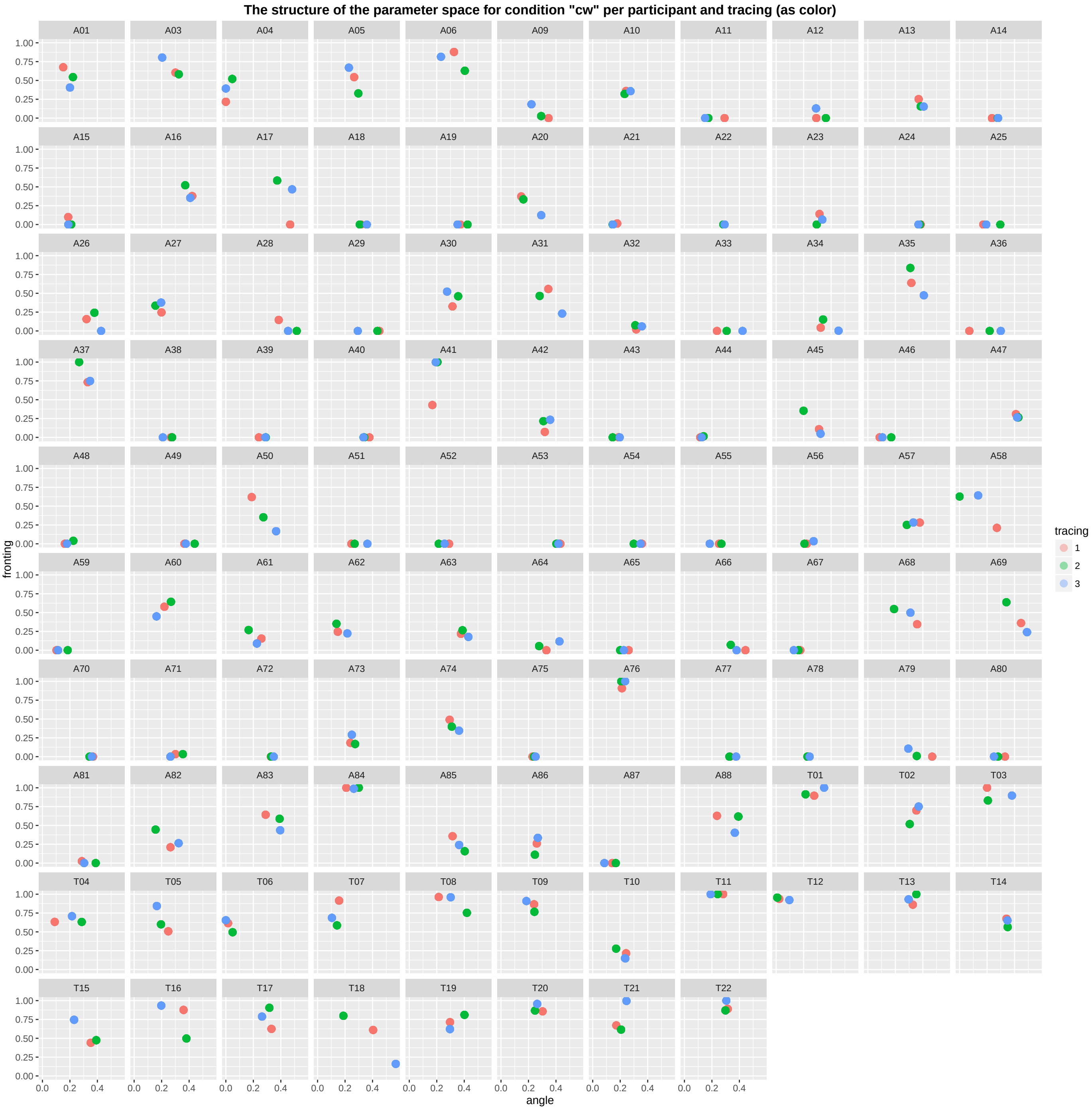

Figure 38: The parameter space across the 100 replications for each participant and tracing in condition "cw".

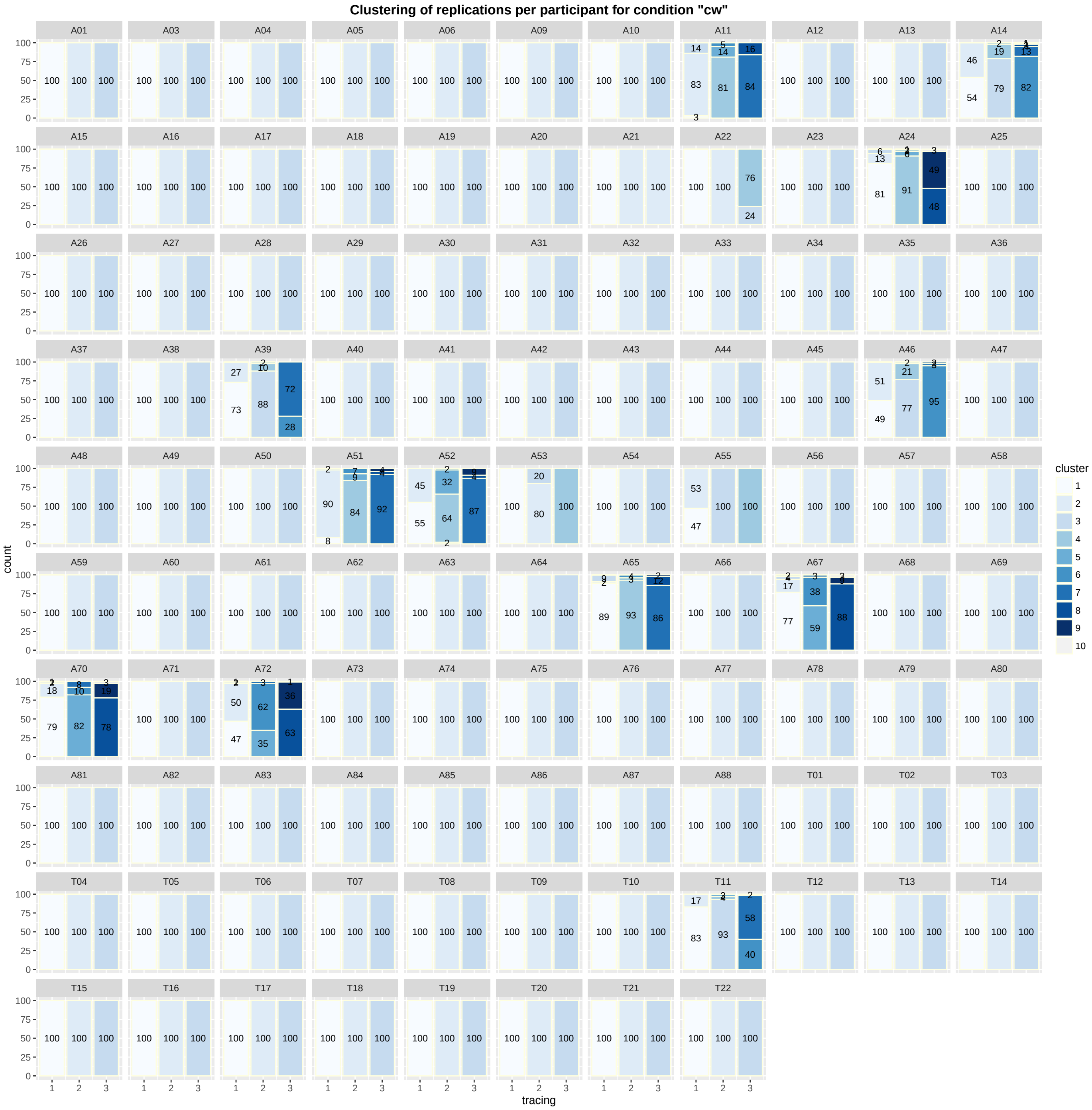

Figure 39: Clustering of replications for each participant and tracing in condition “cw” (some participants might be missing because there are no free parameters with non-constant estimates). Numbers give the number of replications in each cluster. Please note that the clusters are specific to each participant (i.e., cluster ”1” for participant ”X” is different than the cluster ”1” for participant ”Y”).

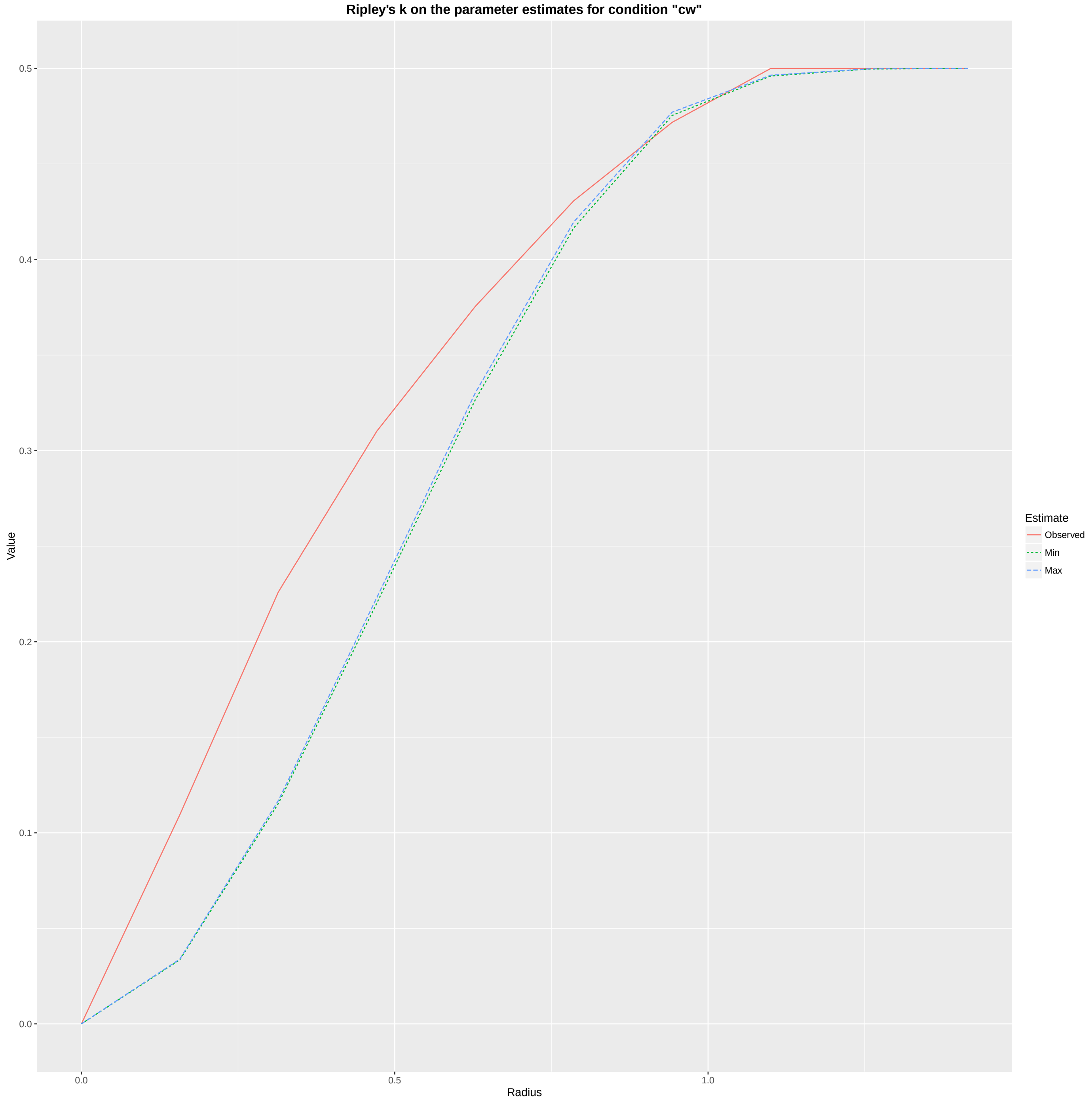

Figure 40: Generalized Ripley's $\hat{k}$ function for the parameter estimates (vertical axis) at 10 distance lags (horizontal axis), showing the actually observed values as well the minimum and maximum values for 100 randomly generated equivalent number of parameter estimates extracted from a Poisson distribution. If the observed $\hat{k}$ is higher than expected then there is significant clustering at that lag, while lower indicate dispersion; otherwise we cannot reject the hypothesis of spatial randomness.

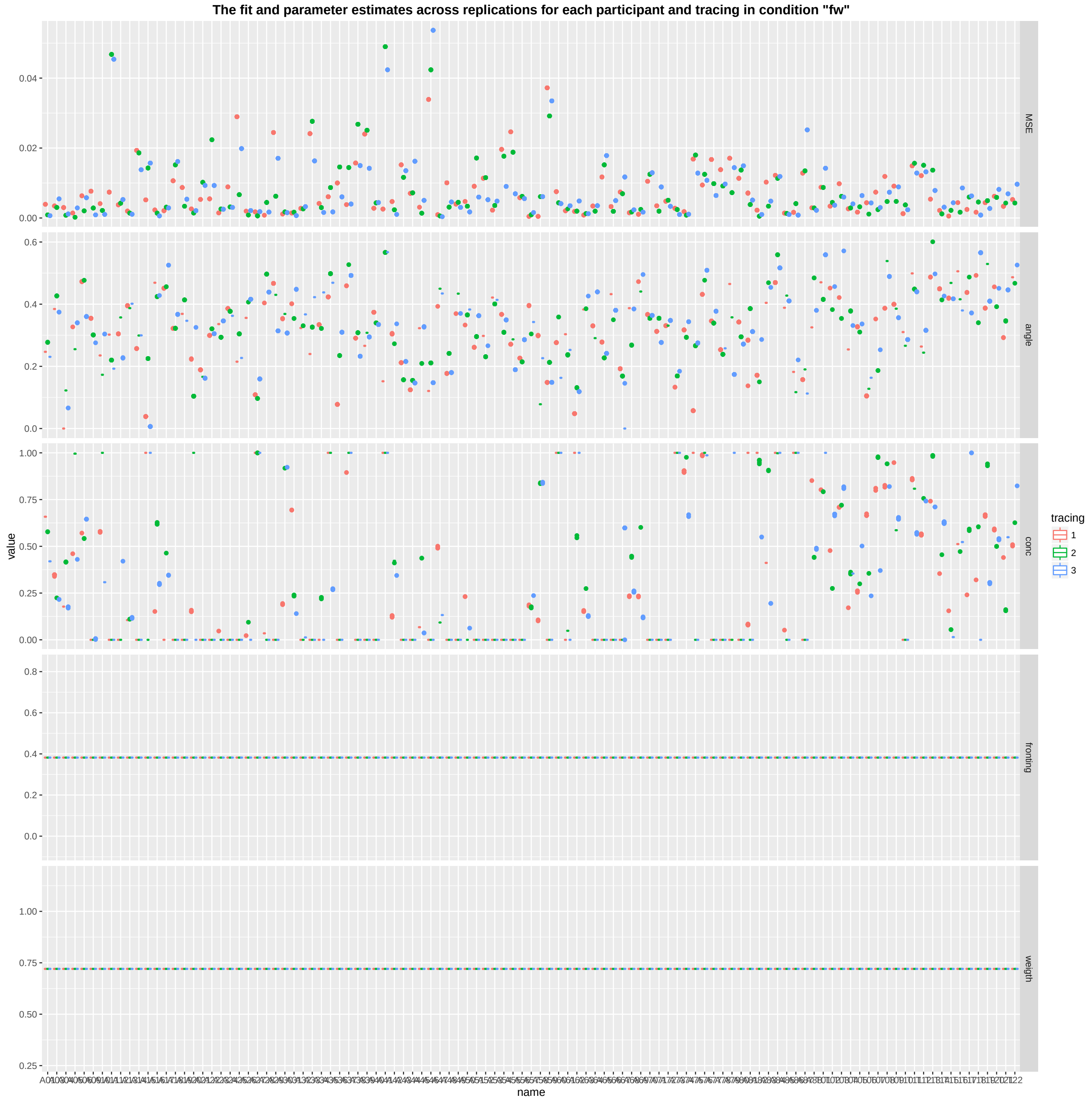

Figure 41: The fit and parameter estimates across the 100 replications for each participant and tracing in condition “fw”.

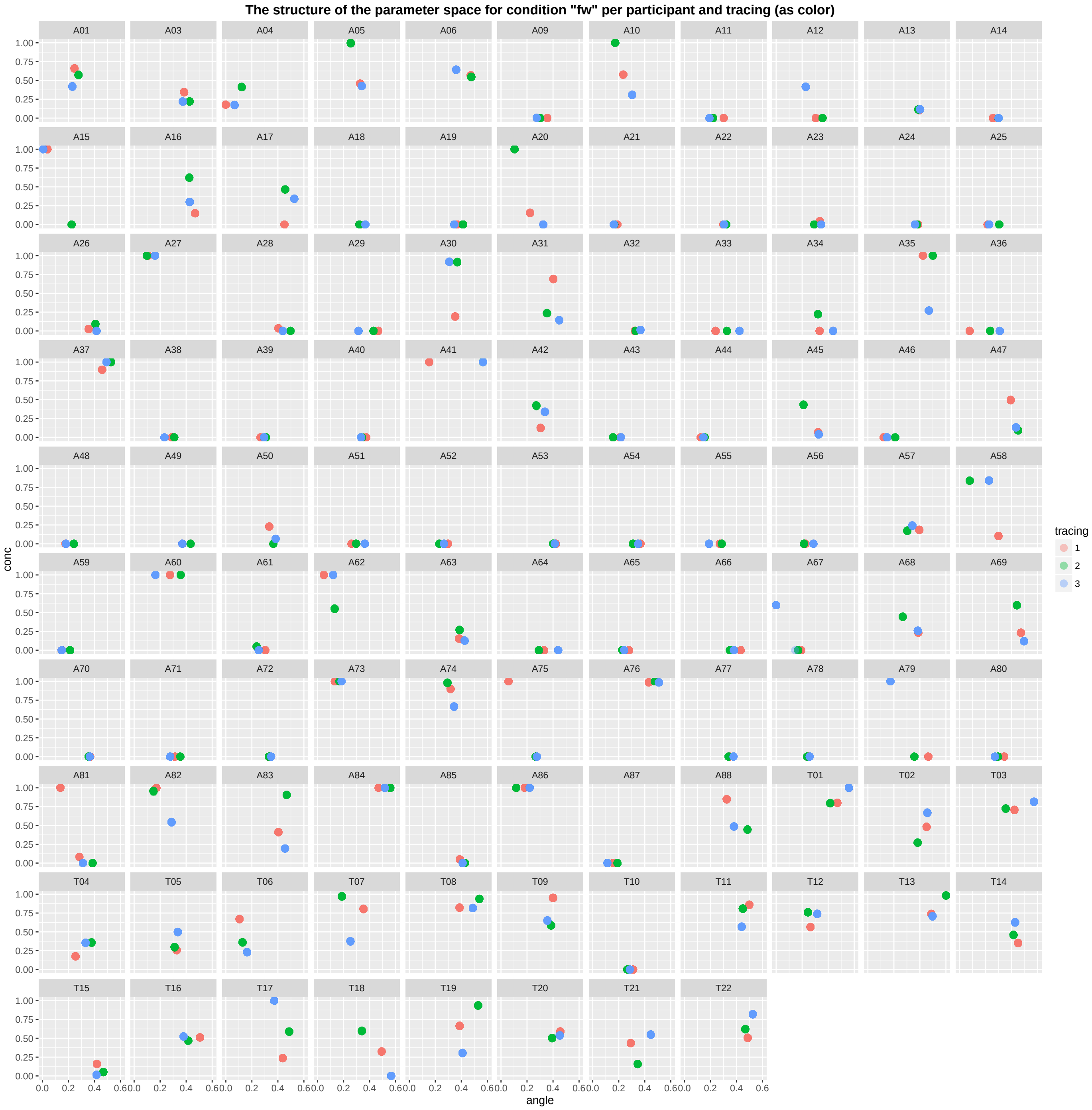

Figure 42: The parameter space across the 100 replications for each participant and tracing in condition “fw”.

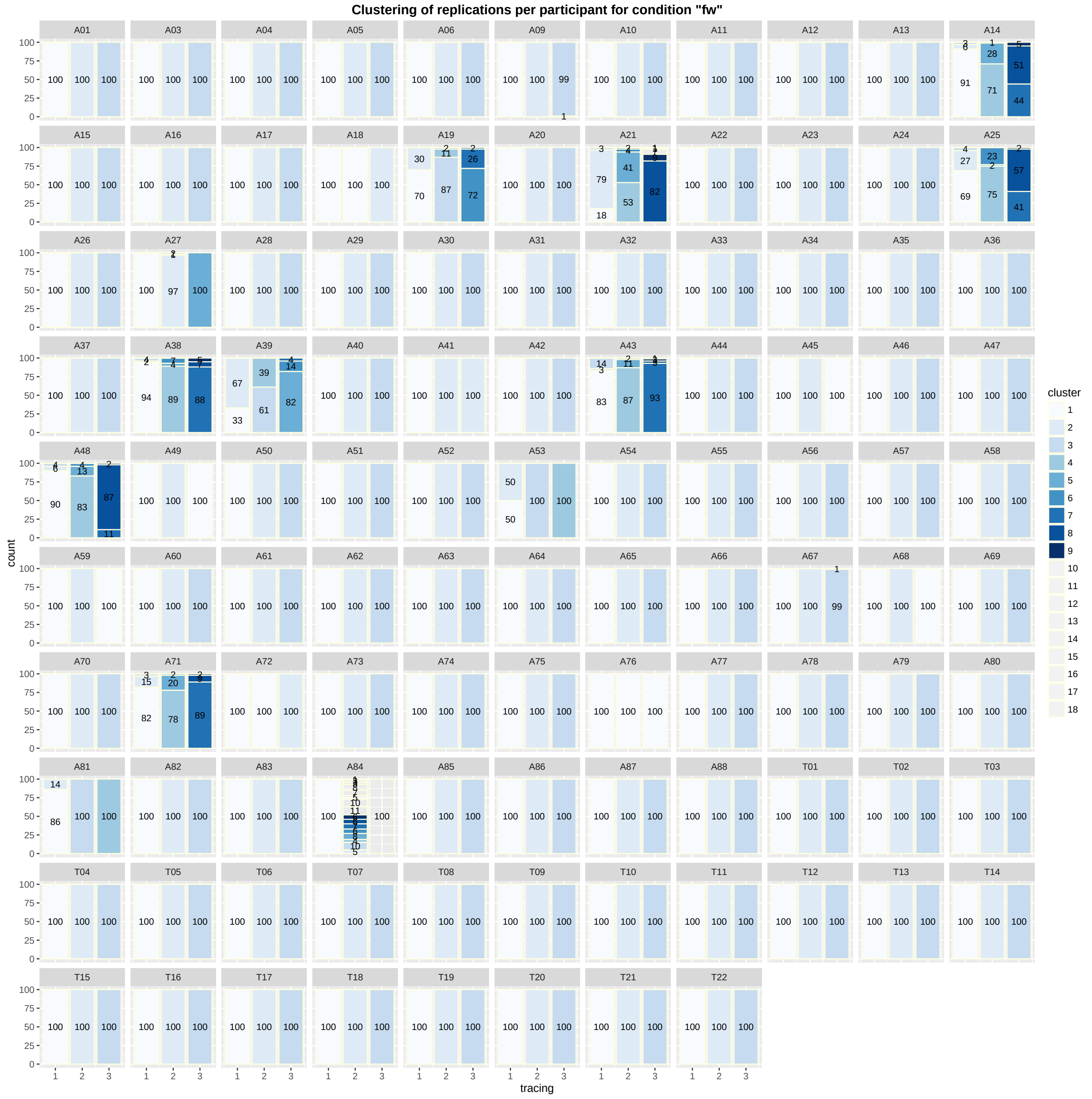

Figure 43: Clustering of replications for each participant and tracing in condition “fw” (some participants might be missing because there are no free parameters with non-constant estimates). Numbers give the number of replications in each cluster. Please note that the clusters are specific to each participant (i.e., cluster ”1” for participant ”X” is different than the cluster ”1” for participant ”Y”).

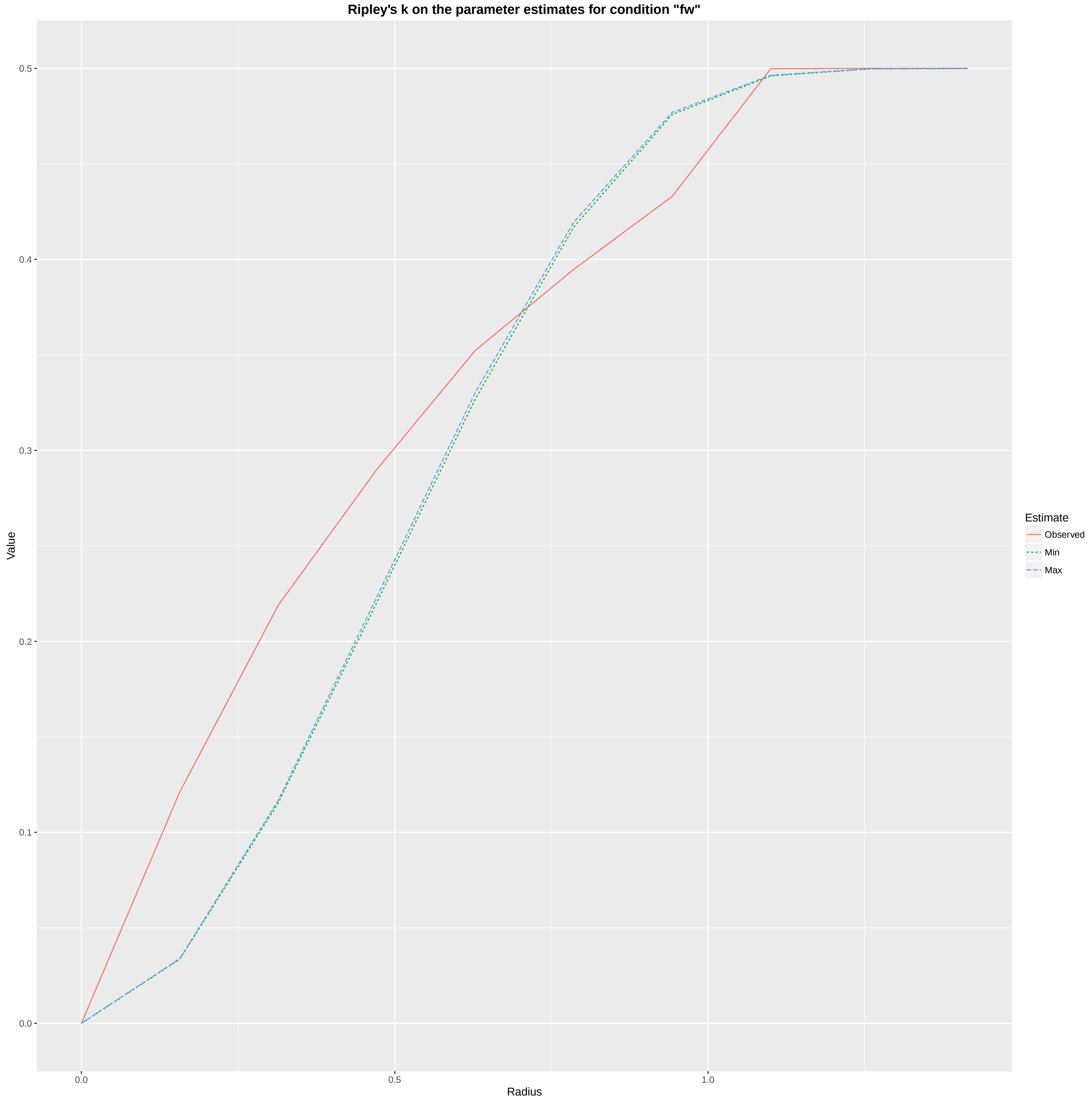

Figure 44: Generalized Ripley's $\hat{k}$ function for the parameter estimates (vertical axis) at 10 distance lags (horizontal axis), showing the actually observed values as well the minimum and maximum values for 100 randomly generated equivalent number of parameter estimates extracted from a Poisson distribution. If the observed $\hat{k}$ is higher than expected then there is significant clustering at that lag, while lower indicate dispersion; otherwise we cannot reject the hypothesis of spatial randomness.

4 Conditions with three fixed parameters (“acw”, ”afw” and ”cfw”)

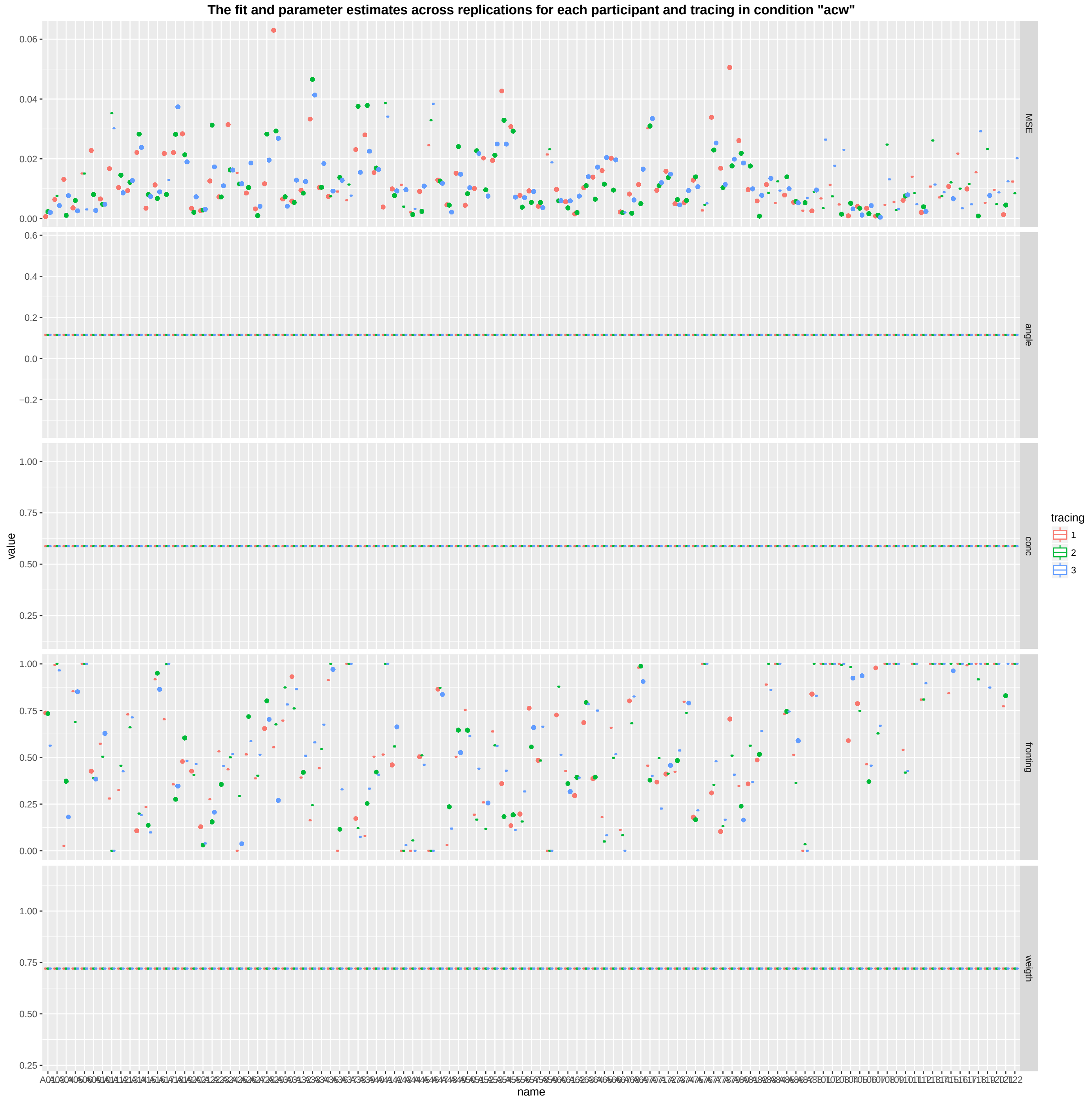

Figure 45: The fit and parameter estimates across the 100 replications for each participant and tracing in condition “acw”.

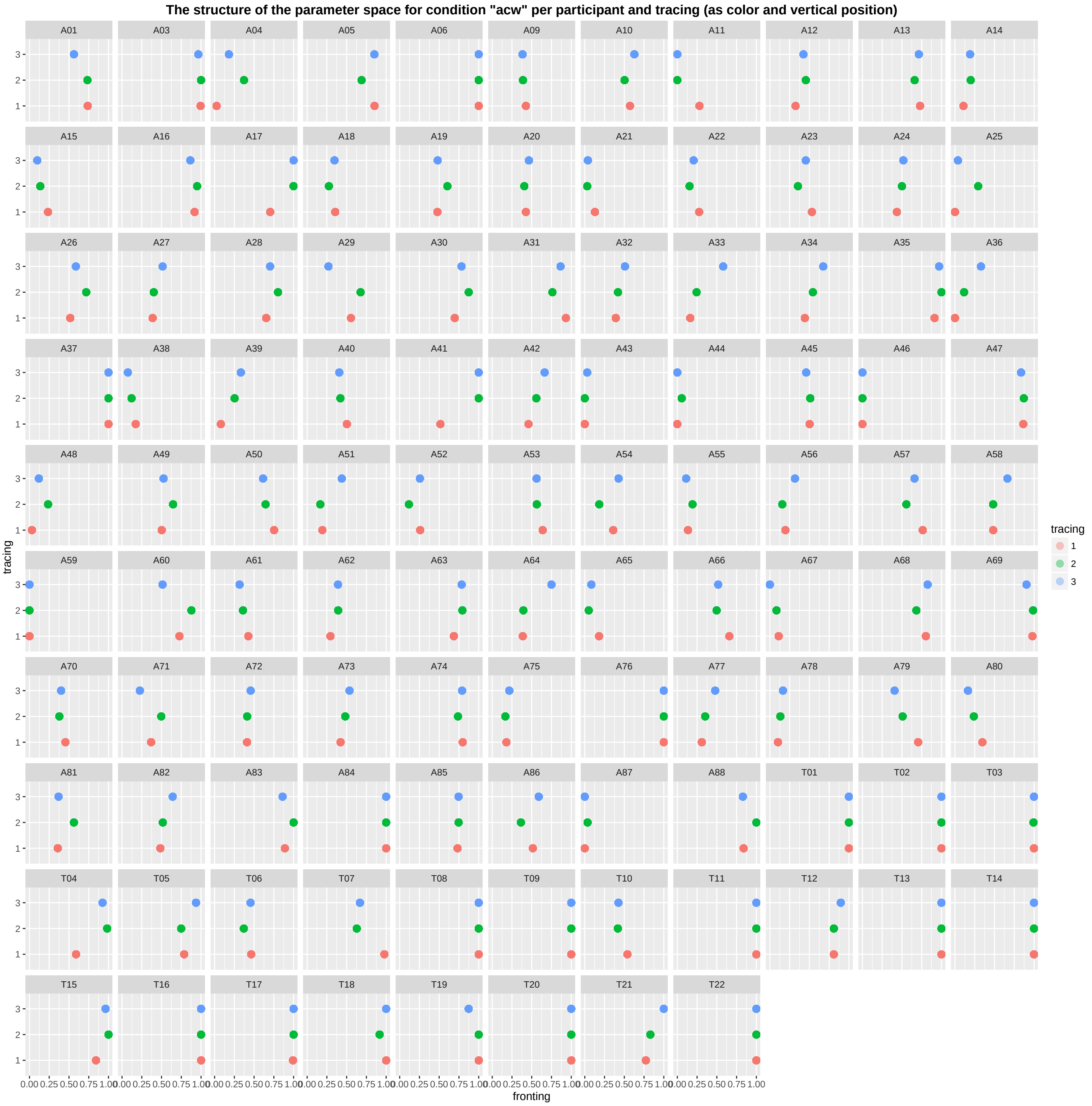

Figure 46: The parameter space across the 100 replications for each participant and tracing in condition “acw”.

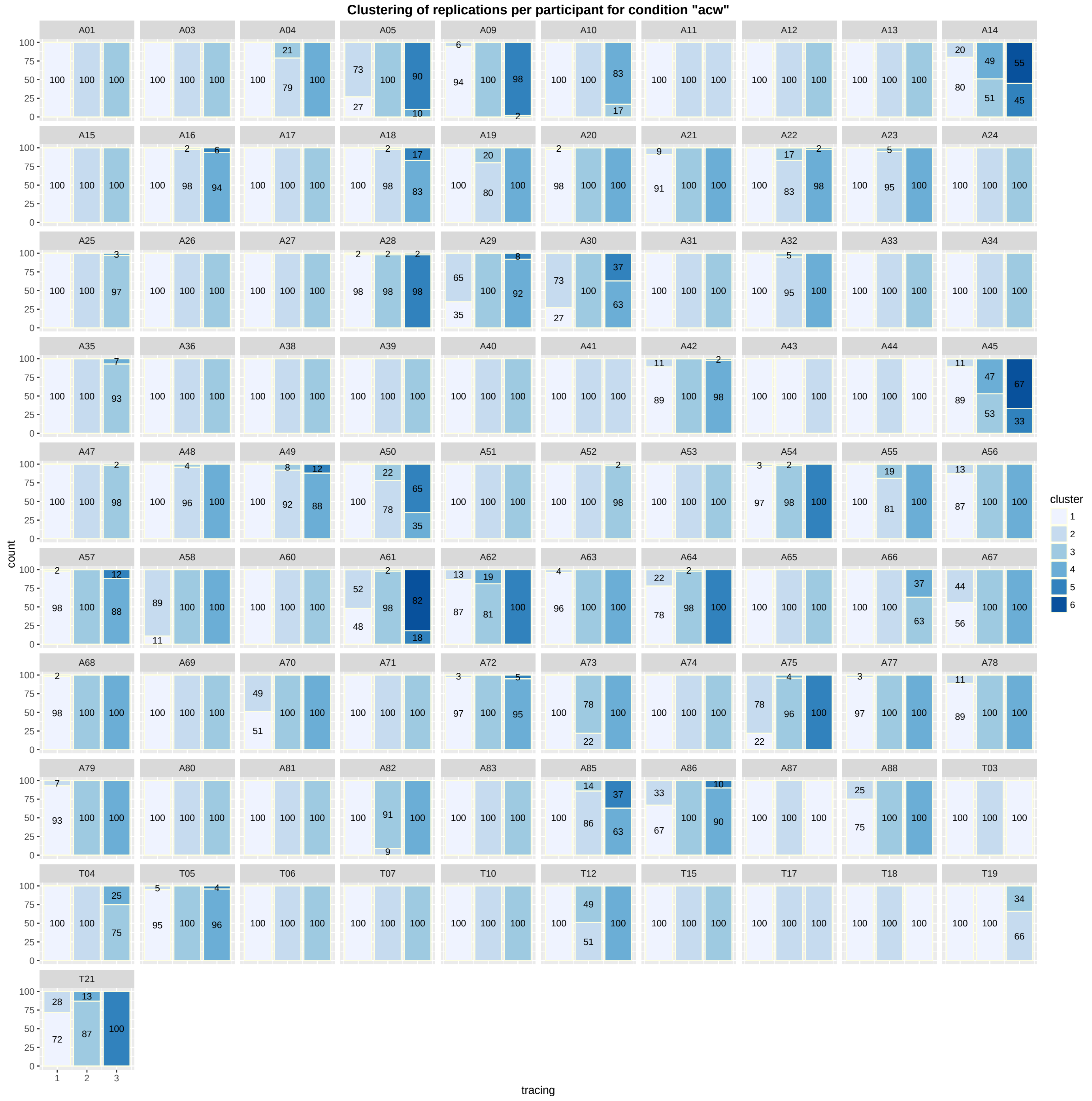

Figure 47: Clustering of replications for each participant and tracing in condition “acw” (some participants might be missing because there are no free parameters with non-constant estimates). Numbers give the number of replications in each cluster. Please note that the clusters are specific to each participant (i.e., cluster ”1” for participant ”X” is different than the cluster ”1” for participant ”Y”).

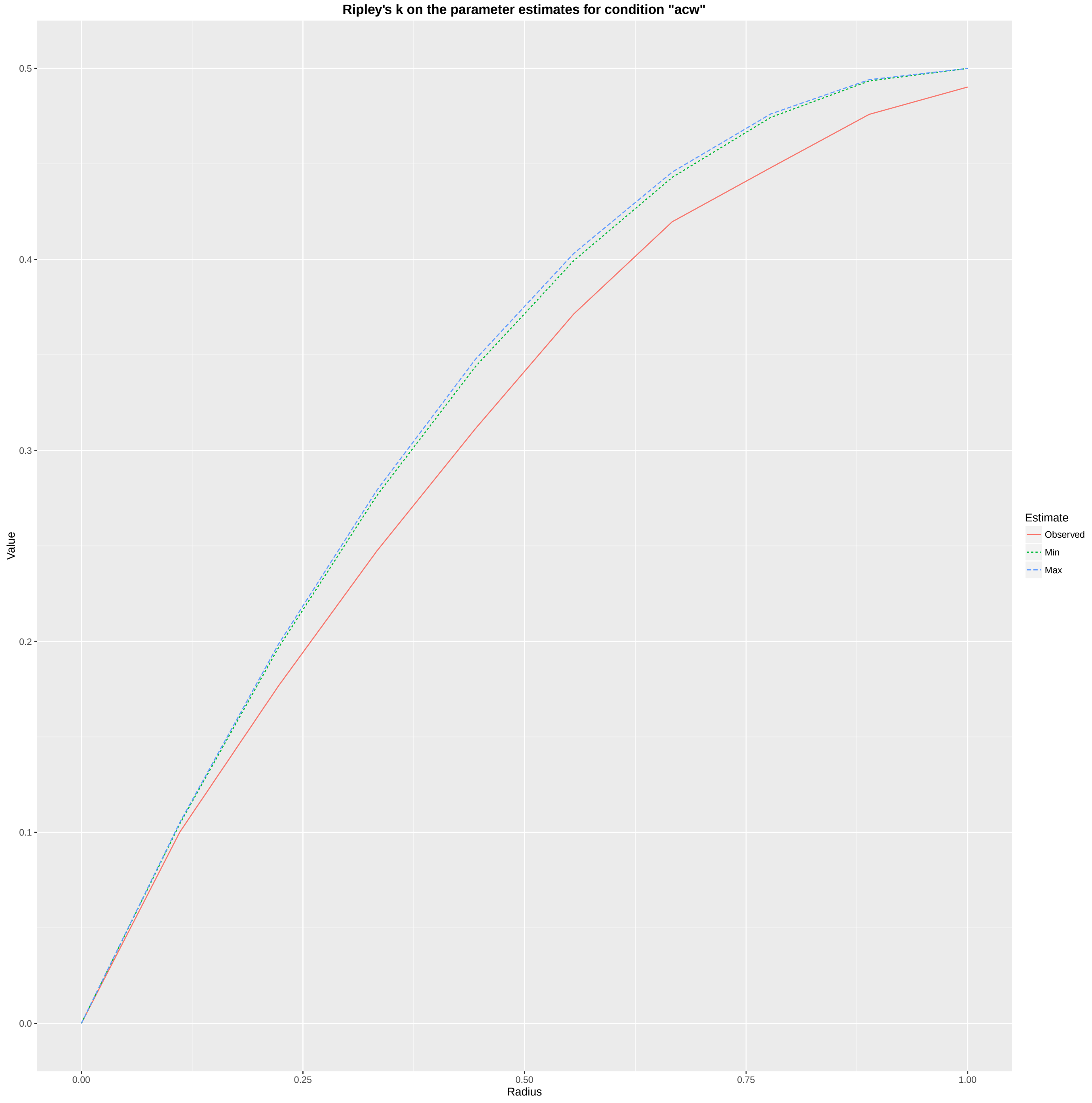

Figure 48: Generalized Ripley's $\hat{k}$ function for the parameter estimates (vertical axis) at 10 distance lags (horizontal axis), showing the actually observed values as well the minimum and maximum values for 100 randomly generated equivalent number of parameter estimates extracted from a Poisson distribution. If the observed $\hat{k}$ is higher than expected then there is significant clustering at that lag, while lower indicate dispersion; otherwise we cannot reject the hypothesis of spatial randomness.

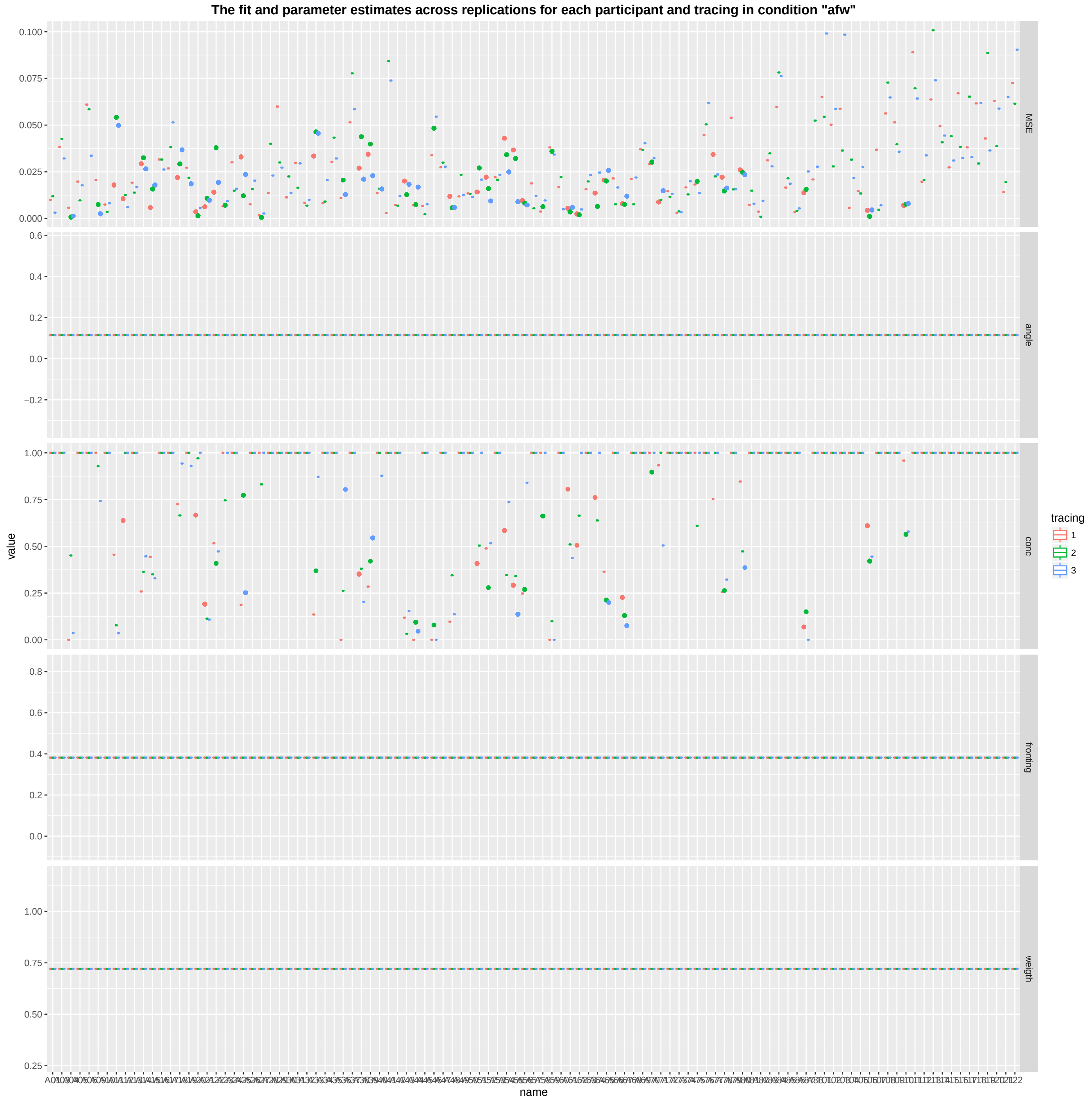

Figure 49: The fit and parameter estimates across the 100 replications for each participant and tracing in condition “afw”.

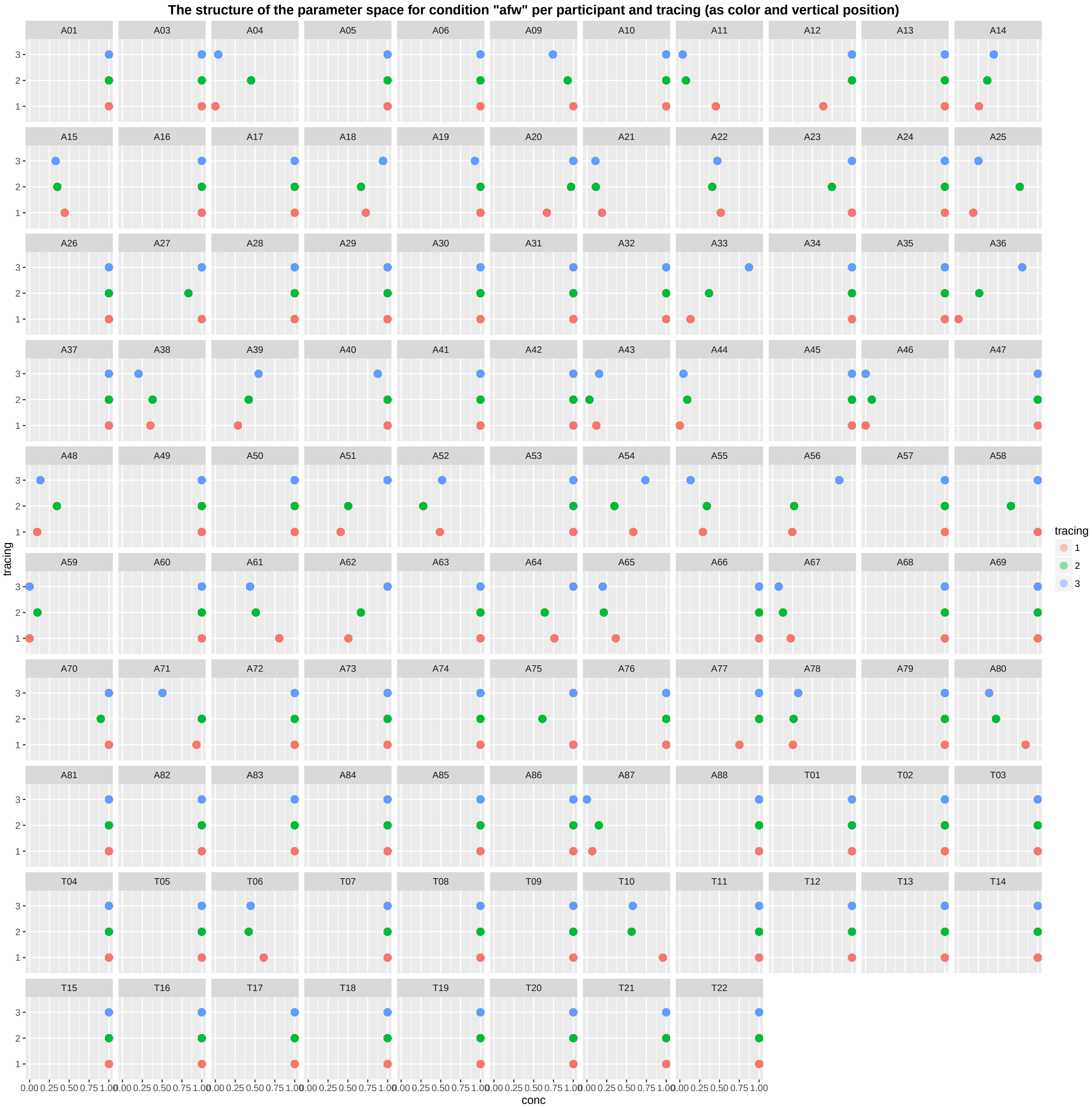

Figure 50: The parameter space across the 100 replications for each participant and tracing in condition “afw”.

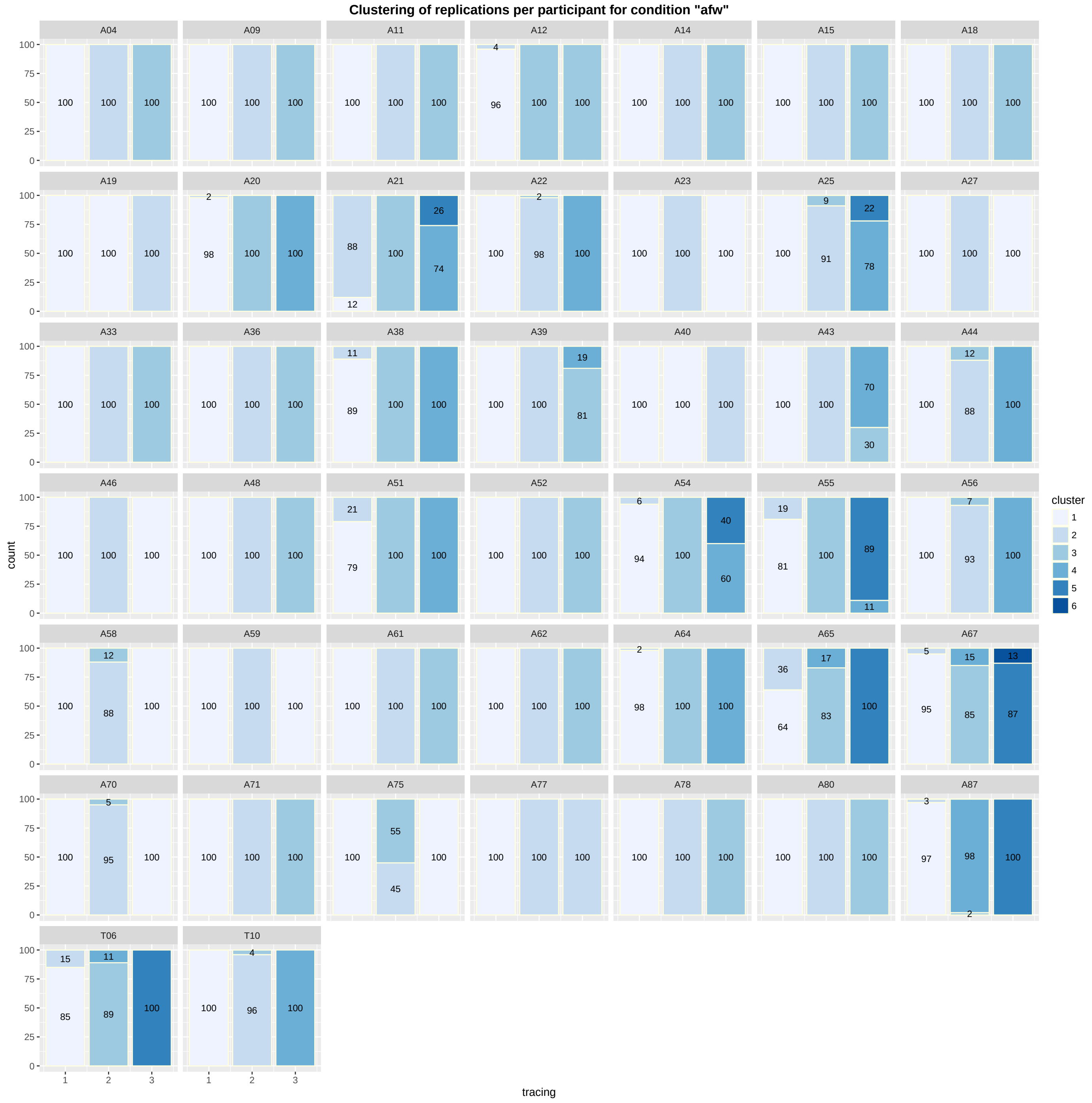

Figure 51: Clustering of replications for each participant and tracing in condition "afw" (some participants might be missing because there are no free parameters with non-constant estimates). Numbers give the number of replications in each cluster. Please note that the clusters are specific to each participant (i.e., cluster "1" for participant "X" is different than the cluster "1" for participant "Y").

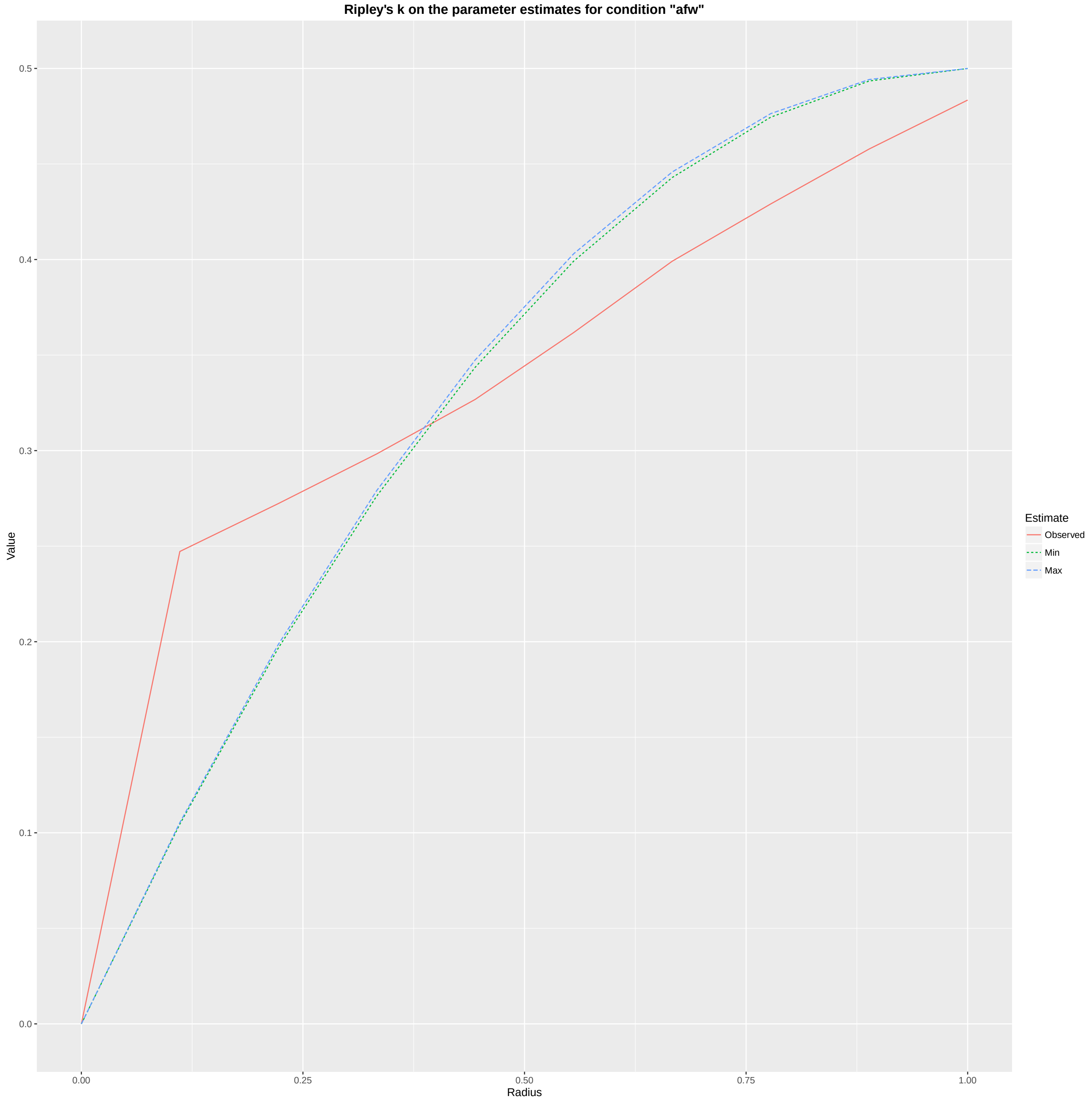

Figure 52: Generalized Ripley's $\hat{k}$ function for the parameter estimates (vertical axis) at 10 distance lags (horizontal axis), showing the actually observed values as well the minimum and maximum values for 100 randomly generated equivalent number of parameter estimates extracted from a Poisson distribution. If the observed $\hat{k}$ is higher than expected then there is significant clustering at that lag, while lower indicate dispersion; otherwise we cannot reject the hypothesis of spatial randomness.

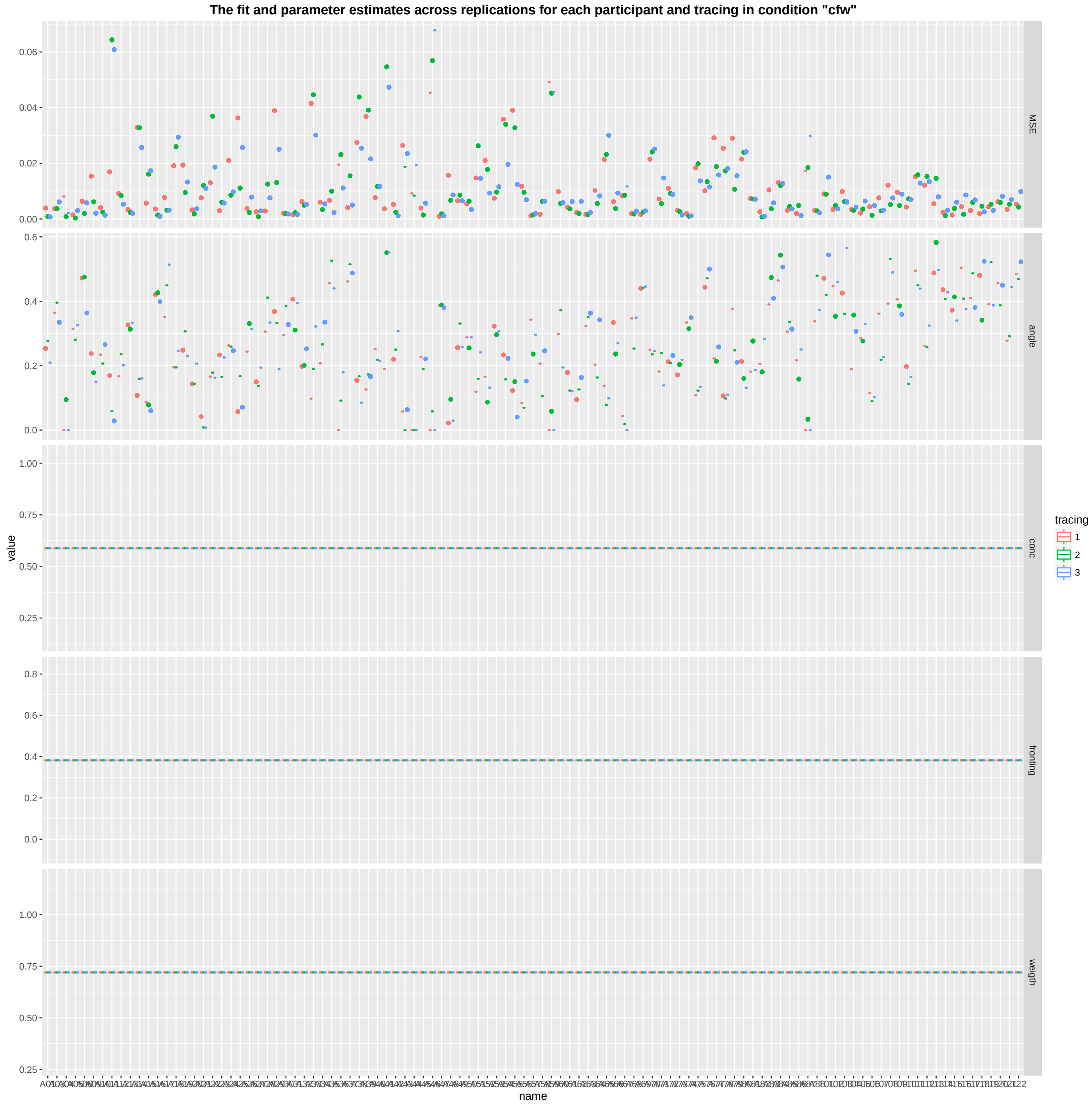

Figure 53: The fit and parameter estimates across the 100 replications for each participant and tracing in condition “cfw”.

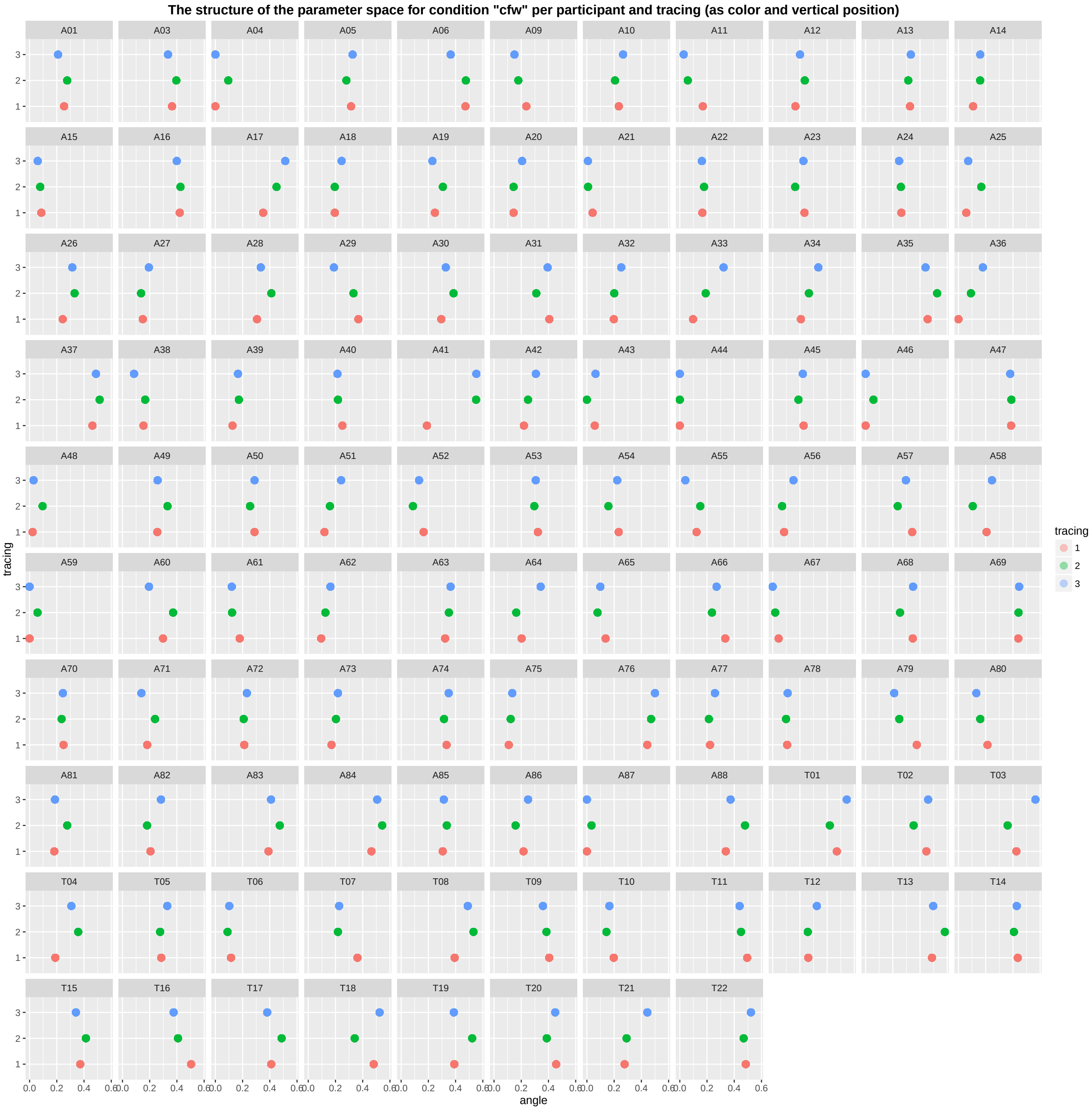

Figure 54: The parameter space across the 100 replications for each participant and tracing in condition “cfw”.

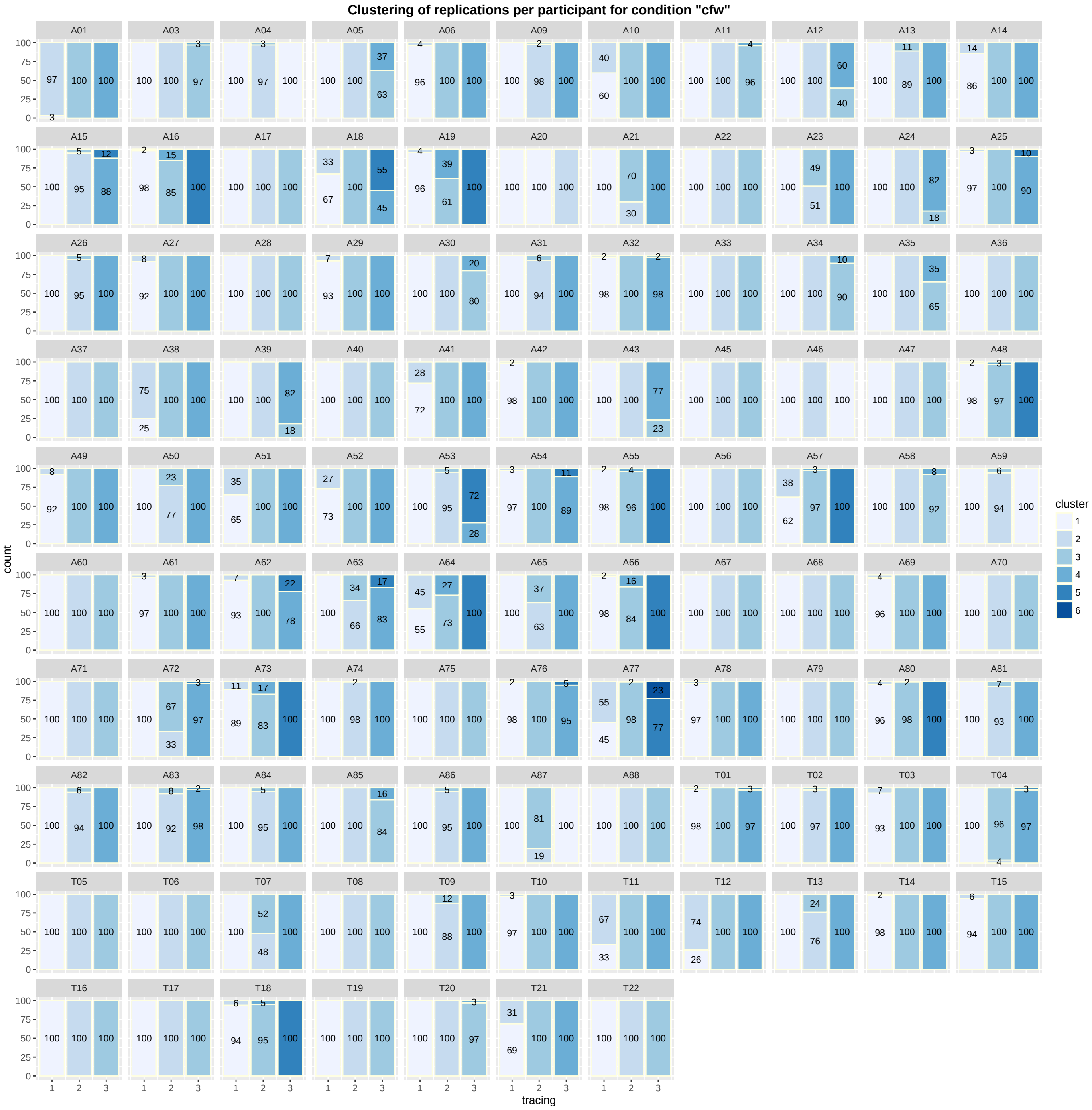

Figure 55: Clustering of replications for each participant and tracing in condition “cfw” (some participants might be missing because there are no free parameters with non-constant estimates). Numbers give the number of replications in each cluster. Please note that the clusters are specific to each participant (i.e., cluster ”1” for participant ”X” is different than the cluster ”1” for participant ”Y”).

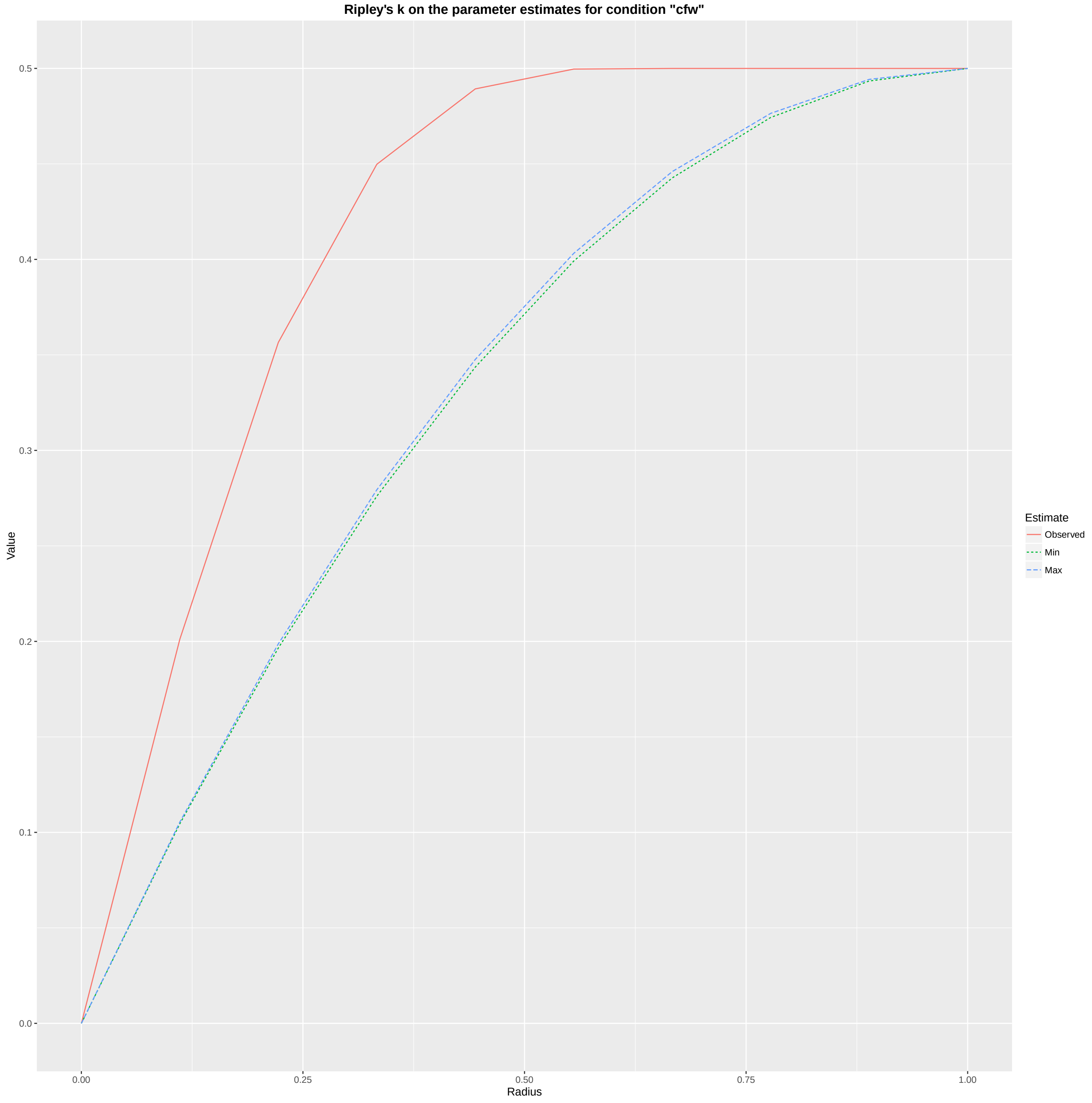

Figure 56: Generalized Ripley's $\hat{k}$ function for the parameter estimates (vertical axis) at 10 distance lags (horizontal axis), showing the actually observed values as well the minimum and maximum values for 100 randomly generated equivalent number of parameter estimates extracted from a Poisson distribution. If the observed $\hat{k}$ is higher than expected then there is significant clustering at that lag, while lower indicate dispersion; otherwise we cannot reject the hypothesis of spatial randomness.

5 The condition with all fixed parameters ("acfw")

Finally, when all parameters are fixed, the goodness-of-fit and parameter estimates per participant and tracing are – as expected – completely fixed (therefore, there no plots to show).
